# Molecular Characterization and Phylogenetic Analysis of MADS-Box Gene *VroAGL11* Associated with Stenospermocarpic Seedlessness in Muscadine Grapes

**DOI:** 10.3390/genes12020232

**Published:** 2021-02-05

**Authors:** M Atikur Rahman, Subramani P Balasubramani, Sheikh M Basha

**Affiliations:** Center for Viticulture & Small Fruit Research, Florida A&M University, Tallahassee, FL 32317, USA; balasubra.paranthama@famu.edu (S.P.B.); mehboob.sheikh@famu.edu (S.M.B.)

**Keywords:** seedlessness, stenospermocarpy, *VroAGL11* gene, qRT-PCR, muscadine grape

## Abstract

Reduced expression of MADS-box gene *AGAMOUS-LIKE11* (*VviAGL11*) is responsible for stenospermocarpic seedlessness in bunch grapes. This study is aimed to characterize the *VviAGL11* orthologous gene (*VroAGL11*) in native muscadine grapes (*Vitis rotundifolia*) at the molecular level and analyze its divergence from other plants. The *VroAGL11* transcripts were found in all muscadine cultivars tested and highly expressed in berries while barely detectable in leaves. RT-PCR and sequencing of predicted ORFs from diverse grape species showed that *AGL11* transcripts were conservatively spliced. The encoded VroAGL11 protein contains highly conserved MADS-MEF2-like domain, MADS domain, K box, putative phosphorylation site and two sumoylation motifs. The muscadine VroAGL11 proteins are almost identical (99%) to that of seeded bunch cultivar, Chardonnay, except in one amino acid (A79G), but differs from mutant protein of seedless bunch grape, Sultanina, in two amino acids, R197L and T210A. Phylogenetic analysis showed that *AGL11* gene of muscadine and other *Vitis* species formed a separate clade than that of other eudicots and monocots. Muscadine grape cultivar “Jane Bell” containing the highest percentage of seed content in berry (7.2% of berry weight) had the highest *VroAGL11* expression, but almost none to nominal expression in seedless cultivars Fry Seedless (muscadine) and Reliance Seedless (bunch). These findings suggest that *VroAGL11* gene controls the seed morphogenesis in muscadine grapes like in bunch grape and can be manipulated to induce stenospermocarpic seedlessness using gene editing technology.

## 1. Introduction

The native muscadine grapes (*Vitis rotundifolia*) are grown in the Southeastern USA mainly for fresh fruit and wine production, generating USD 1.6 billion total taxes in 2017 alone in Florida [1]. They have long been cherished for their distinct fruity flavor and a number of health benefits including antioxidant, anti-inflammatory, anti-cancer, and anti-aging potentiality [2,3,4]. Despite having high nutraceutical value and numerous health benefits, muscadine grapes are not preferred as table grape over European bunch grape due to presence of large seeds. To sustain the muscadine grape industry in the Southeastern USA and meet the ever-increasing demand for table grapes in the fresh fruit market, the muscadine grape industry strives to develop seedless muscadine grapes.

Seedlessness is the most prized trait for developing the seedless table grape cultivar, which can occur through two biological processes, namely, parthenocarpy and stenospermocarpy. Fertilization does not occur during fruit development in parthenocarpy, producing small berries without seeds. In stenospermocarpy, fertilization occurs but seed development is prematurely aborted due to cessation of seed coat and endosperm development [5,6], producing fruits with greatly reduced-size seeds called seed traces [7,8]. Unlike parthenocarpy, in stenospermocarpy the berry size is not, or less, compromised; therefore, stenospermocarpic seedlessness is more popularly used in the production of seedless table grape cultivars. However, stenospermocarpic seedlessness does not occur or has not been reported so far in muscadine grapes. The only available seedless muscadine genotype “Fry Seedless” is parthenocarpic and cannot be used as crossing parent in the breeding program due to male sterility [9].

Several studies have been conducted to investigate the genetic basis of seedlessness found in an ancient oriental cultivar, Sultanina, or Sultanina-derived grape cultivars (e.g., Thomson seedless, Crimson seedless) during the last two decades. Sultanina or Thomson seedless produces almost unnoticeable seed traces and has been used as the main donor of stenospermocarpic seedlessness in table grape breeding programs throughout the world [10,11,12,13,14]. The genetic inheritance of seedlessness in grapes has been reported to be controlled by a dominant allele present in the region named *SEED DEVELOPMENT INHIBITOR* (*SDI*) locus [7,15]. Later, different quantitative studies confirmed that a major QTL (quantitative trait locus) was co-localized with this SDI locus on linkage group 18, explaining 50–70% of the phenotypic variance of the trait [8,16,17,18,19]. A grape MADS-box transcription factor gene, *AGAMOUS-LIKE11* (*VviAGL11*, Vv18s0041g01880) was identified (mapped) in the SDI locus through in-silico analysis and proposed as the SDI candidate gene for seedlessness in grapes based on genetic linkage and putative homology with the Arabidopsis MADS-box transcription factor gene, *AtAGL11* (At4g09960) [8,17]. The *AtAGL11*, an ortholog of *VviAGL11*, controls the ovule and seed development in Arabidopsis which is reflected in its mutant *SEEDSTICK* phenotype showing reduced number and size of seeds [20,21,22,23,24]. 

The bunch grape gene *VviAGL11* consists of eight exons spanning ~7.6 kb with a coding region of 672 bp (NCBI: KM401845 cv. Chardonnay) [22]. The *VviAGL11* gene is highly expressed in floral and fruit tissues of bunch grapes but repressed in roots, branches, leaves, buds, and tendrils [20]. The expression of *VviAGL11* in pea-size berries (when seeds begin to develop) was 25 times higher in the seeded homozygous genotype compared to flower stage in seedless homozygous grape genotype [8]. In situ hybridization studies showed the accumulation of *VviAGL11* transcripts in the dual endotesta layer of seeds in seeded grape genotypes but could not detect the transcripts in the seedless grape tissues [22]. The wild-type phenotype of Arabidopsis was restored by the ectopic expression of *VviAGL11* in the *SEEDSTICK* mutant, showing its direct role in seed morphogenesis [22]. The reduced expression of the *VviAGL11* gene was reported as the source or origin of stenospermocarpic seedlessness in “Sultanina” and Sultanina-derived grape cultivars [8,22,25,26]. The overexpression of the *VviAGL11* gene in seedless grape cultivar “Linda” produced small seeds in the seedless berries, while silencing of *VviAGL11* gene in seeded bunch grape cultivars (Italia, Ruby) induced the seedlessness by decreasing the seed content [27]. Malabarba and her coworkers [27] thus confirmed that *VviAGL11* gene is the key master regulator of seed morphogenesis in bunch grapes and can be manipulated for inducing seedlessness in bunch grapes. 

However, the role of *AGL11* gene for stenospermocarpic seedlessness has not been characterized at the molecular level in muscadine grapes. This study is a part of our overall aim to develop seedless muscadine table grape, through molecular understanding and divergence of the *AGL11* orthologous gene in muscadine grapes (*VroAGL11*). The specific goals of the study were to (1) identify and quantify the *VroAGL11* transcript levels in different plant parts and berry developmental stages of muscadine grape, (2) determine the relationship of its expression level with the seed size, weight and number of selected seeded and seedless muscadine and hybrid bunch/bunch grape cultivars, and (3) sequence their *VroAGL11* transcripts to explore the sequence variation at transcriptomic levels and study/analyze the divergence of *VroAGL11* gene in muscadine grapes from other monocot and eudicot plants.

## 2. Materials and Methods 

### 2.1. Plant Materials and Growth Conditions 

Grape berries of muscadine, Florida hybrid bunch, and bunch grape cultivars grown at the Center for Viticulture and Small Fruit Research at Florida A&M University (FAMU), Tallahassee were used for this study. Bunch grape cultivars were grown and maintained in a screen cage (20 × 40 ft) at the FAMU Viticulture Center to protect them from glossy wing sharpshooter insects responsible for spreading deadly Pierce’s disease. Standard management practices including fertilizing, irrigation, pesticide application and pruning at regular intervals were followed to keep the grape plants healthy.

### 2.2. Berry and Seed Characteristics

A total of 20 harvest-ripe berries (EL 38) [28] of 42 muscadine (*Vitis rotundifolia*), 2 Florida hybrid bunch grape (*Vitis* spp.) and 1 bunch grape (*Vitis vinifera*) cultivars including seedless cultivars were randomly collected from at least 3 vines per cultivar in June/July (bunch and hybrid bunch) and September (Muscadine) during the 2020 season to study the berry and seed characteristics (Table 1). The mean berry weight (= BW/BN), seed number/berry (= SN/BN) and seed weight/berry (= SW/BN) and seed content of berry (=(SW/BW) * 100) were measured, where BW = berry weight, BN = berry number, SN = seed number, and SW = seed weight. The seed length and width of at least 12 seeds per cultivar were measured using electronic digital calipers (Thomas Scientific, Swedesboro, NJ, USA).

### 2.3. RNA Isolation and cDNA Synthesis

Samples were randomly collected from young leaf (EL 16), inflorescence (EL 23) and berries at different developmental stages viz. pea-size (~7 mm diameter, EL 31), green and mature (EL 33), veraison (EL 35) and harvest-ripe stage (EL 38) [28]) from three grapevines of selected seeded and seedless muscadine (cvs. Black Beauty, Black Fry, Dixie Red, Jane Bell, Noble, Fry, Fry Seedless), hybrid bunch grape (cv. Blanc du Bois, BDB), and bunch grape (cvs. Riesling (*Vitis vinifera*), Reliance Seedless (*Vitis labrusca*)) cultivars representing different berry sizes. All the samples were collected between 9 a.m. and 9:30 a.m. (Muscadine: July–September, bunch/hybrid bunch: April–June) and frozen immediately in liquid N_2_ and stored at −80 °C until further use. Total RNA was isolated from the frozen berries using the Spectrum Plant Total RNA Kit (Sigma-Aldrich) following the manufacturer’s instructions with some modification [29] and treated with DNase using the TURBO DNA-free Kit (Invitrogen) to remove the genomic DNA. The quantity and quality, and integrity number (RIN) of RNAs were measured using a NanoDrop 2000c (Thermo Scientific, Waltham, MA, USA) and Agilent Bioanalyzer 2100 (Agilent Technologies, Santa Clara, CA, USA), respectively. The total RNA with RIN >8.0 were converted to cDNA using SuperScript III First-Strand synthesis system (Life Technologies, Carlsbad, CA, USA) and oligo (dT) primer as per the manufacturer’s protocol.

### 2.4. Detection and Confirmation of Muscadine Grape AGL11 Transcripts

The muscadine grape *AGL11* transcripts were detected by performing qRT-PCR in CFX96 Touch real-time PCR system (Bio-Rad) using the SsoAdvanced Universal SYBR Green Supermix (Bio-Rad) in different plant parts and at different berry developmental stages of muscadine grape cultivar, Noble. The gene-specific qRT-PCR primers were designed (Table S1) based on the bunch grape (*Vitis vinifera*) genome available at NCBI using Primer-BLAST tool. The experiment was repeated thrice on three biological samples with three technical replicates per sample (details in Section 2.5).

To confirm the presence of the *AGL11* transcripts, the predicted full-length open reading frame (ORF) was amplified from cDNAs of selected muscadine grape (cvs. Jane Bell, Black Beauty, Fry, Fry Seedless), hybrid bunch grape (cv. BDB), bunch grape (cvs. Riesling, Reliance Seedless) cultivars by nested PCR with KOD Hot Start DNA polymerase (Novagen) using 2 pairs of gene-specific primers (Table S1) designed from *Vitis vinifera* genome available at NCBI. PCR products of the predicted open reading frame were purified from 1% agarose gels using the QIAquick gel extraction kit (Qiagen) and sequenced at the Eurofins Genomics service center.

### 2.5. Quantification of Relative Gene Expressions

The real-time PCR experiments were performed on cDNA (100 ng) from leaf, inflorescence, berry tissues of selected seeded and seedless muscadine and bunch grape cultivars to quantify the expression levels of *AGL11* orthologous gene. qRT-PCR assay was performed in CFX96 Touch real-time PCR system (Bio-Rad Laboratories, Berkeley, CA, USA) using the SsoAdvanced Universal SYBR Green Supermix (Bio-Rad) and same gene-specific qRT-PCR primers used for transcript detection (Table S1). The thermal cycling program used was: initial polymerase activation and DNA denaturation at 95 °C for 30 s, followed by 40 cycles of amplification consisting of denaturation at 95 °C for 10 s and annealing/extension at 60 °C for 30 s with a final melt-curve analysis at 65–95 °C @ 0.5 °C increment for 5 s/step. The experiment was conducted on three biological samples with three technical replicates per sample. Grape housekeeping genes viz., Actin and Glyceraldehyde-3-Phosphate Dehydrogenase (GAPDH) were used as reference genes. The “delta-delta CT” (2-∆∆CT) method was used for comparing relative expressions of genes associated with seedlessness between samples of selected grape cultivars [30], where ∆∆CT = ∆CT sample—∆CT control, and CT = threshold cycle.

### 2.6. DNA Isolation and Sequencing of Muscadine AGL11 Gene

The *AGL11* gene was identified in the bunch grape genome (*Vitis vinifera*) at the Genbank of NCBI (https://www.ncbi.nlm.nih.gov/) (last accessed on 23 November 2020) and at Grape Genome Browser (12×) of Genoscope (http://www.genoscope.cns.fr/externe/GenomeBrowser/Vitis/) (last accessed on 23 November 2020). Genomic DNA was extracted from frozen leaf tissues of selected seeded and seedless muscadine grape cultivars according to Kim et al. [31] with little modification. The PCR amplification of muscadine *AGL11* (*VroAGL11*) gene was accomplished with gene-specific primer pairs designed from bunch grape genome available at NCBI using KOD Hot Start Polymerase (Novagen). Due to the large size (6.8 kb) of the gene, 8 sets of primer pairs were used to amplify overlapping sequences of the *VroAGL11* genes (Table S1). The amplicon was isolated from 1% agarose gels using the QIAquick gel extraction kit (Qiagen), A-tailed, ligated into pGEM-T Easy vector (Promega) and transformed into *E. coli* DH5α. Colonies selected with appropriate antibiotics (50 µg/mL Amp) were verified by colony PCR. Plasmids containing inserts of putative interest were isolated and sent for sequencing at the Eurofins Genomics service center. The sequences determined from full-length ORFs amplified on isolated RNA were used to confirm proper annotation and reconstruction of the genomic sequences. 

### 2.7. Analysis of Deduced Protein Sequences

The encoded amino acid sequences of ORFs of *AGL11* gene from selected seeded and seedless muscadine grape, hybrid bunch and bunch grape cultivars were deduced by Six-Frame Translation (https://www.bioline.com/media/calculator/01_13.html) (accessed on 9 October 2020) and aligned by Clustal Omega (https://www.ebi.ac.uk/Tools/msa/clustalo) (accessed on 9 October 2020) along with *AGL11* ORFs of bunch grape cultivars Chardonnay (AKJ79177.1, AKJ79178.1) and Sultanina (AKJ79179.1, AKJ79180.1). Conserved domains and putative phosphorylation sites of the muscadine AGL11 protein were identified using “Identify conserved domains” at NCBI. The “epestfind” (http://emboss.bioinformatics.nl/cgi-bin/emboss/epestfind) (accessed on 2 November 2020) and SUMOplot™ Analysis Program (https://www.abcepta.com/sumoplot) (accessed on 2 November 2020) were used to identify the PEST motifs (as potential proteolytic cleavage site) and sumoylation sites (with score ≥ 0.5), respectively. “Isoelectric Point Calculator” (http://isoelectric.org/calculate.php) (accessed 2 November 2020) was used to calculate the isoelectric point (pI) and molecular weight (MW) of each putative protein. The putative nuclear localization signals (NLSs) specific to the importin αβ pathway were identified by the cNLS Mapper (http://nls-mapper.iab.keio. Ac.jp/cgi-bin/NLS_Mapper_ form.cgi) (accessed on 2 November 2020) calculating NLS scores but not by the conventional sequence similarity search [32].

### 2.8. Phylogenetic Tree Constructions

To construct the phylogenetic tree, 61 amino acid sequences of AGL11 or AGL11-like proteins of monocots and eudicot plants were obtained from NCBI database BLAST-ing AGL11 amino acid sequences of *Vitis vinifera* (cv Chardonnay), *Arabidopsis thaliana* and *Triticum aestivum* as query sequences and aligned along with 4 grape species in the PHYLIP format using MUSCLE 3.8.31 program (http://www.phylogeny.fr/one_task.cgi?tasktype=muscle) (accessed on 29 October 2020). The maximum-likelihood phylogenetic tree was constructed by the PhyML 3.0 [33] using the best-fit amino acid substitution model LG (Le and Gascuel) [34], starting tree BIONJ and 100 replicates of bootstrap analysis. The LG substitution model was selected as the best-fit model by SMS (Smart Model Selection) tool [35] based on Akaike Information Criterion (AIC). The phylogenetic tree was visualized using the PRESTO (Phylogenetic tree visualization) tool with displaying bootstrap values.

### 2.9. Statistical Analysis

The statistical significance of the differences between the means calculated for the various parameters was determined by one-way ANOVA and Tukey HSD post-hoc test using the statistical software package R [36]. The correlation between two parameters and the *p* values were calculated using MS excel.

### 2.10. Accession Numbers

The following sequences were deposited in GenBank: Muscadine grape cv. Jane Bell (*VroAGL11_JB*) genomic DNA [MW447147] and mRNA [MW151564], cv. Fry (*VroAGL11_F*) mRNA [MW151565], cv. Black Beauty (*VroAGL11_BB*) mRNA [MW151566], and cv. Fry Seedless (*VroAGL11_FS*) mRNA [MW151567], Florida hybrid bunch (*Vitis sp*) grape cv. Blanc du Bois (*VspAGL11_BDB*) mRNA [MW151568], bunch grape (*Vitis vinifera*) cv. Riesling (*VviAGL11_RL*) mRNA [MW1515649] and bunch grape (*Vitis labrusca*) cv. Reliance Seedless (*VlaAGL11_RS*) mRNA [MW151570].

## 3. Results

### 3.1. Berry and Seed Characteristics

Mean berry weight of muscadine grapes varied from 2.5 to 15.9 g depending on cultivar with the maximum weight of 15.9 g in Black Beauty and minimum weight (2.5 g) in Fry Seedless followed by muscadine cultivar Welder (3.3 g) (Table 1). However, the mean berry weight of bunch/hybrid bunch grapes were less compared to muscadine grapes ranging from 1.6 to 2.0 g. The mean number of seeds in muscadine grape berries ranged between 2.3 and 4.9 per berry with the highest number of seeds in Janet (5) and the lowest in Scuppernong (2). In bunch or hybrid bunch grapes, however, the mean seed number varied from 1.3 to 3.6 per berry. 

**Figure 1:**
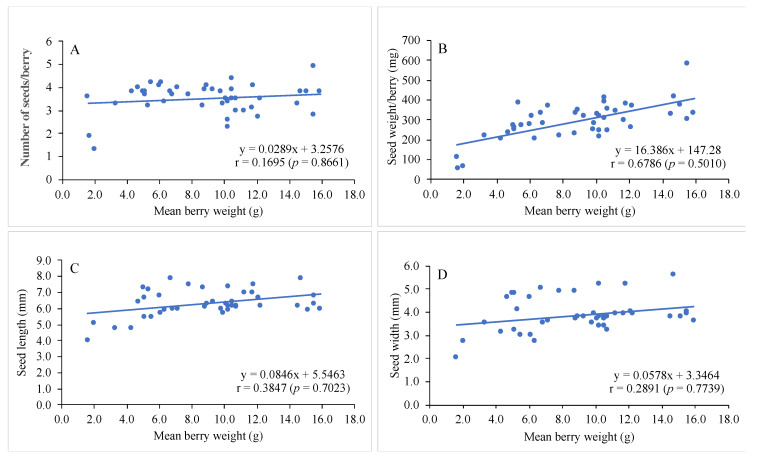
Scatter plots showing relationship between mean berry weight and (**A**) number of seeds/berry, (**B**) seed weight/berry; (**C**) seed length, and (**D**) seed width of muscadine and bunch grape cultivars. r = coefficient of correlation and *p* = significance level.

Muscadine grape berries contained seeds of different size and weight varying between 4.8 and 7.9 mm in length, 2.7 and 5.6 mm in width and 54 and 120 mg/seed depending on the cultivar (Table 1). The longest and widest seeds were found in muscadine cultivars “Ison” (7.9 mm) and “Late Fry” (5.6 mm), respectively, but the highest mean seed weight was found in muscadine cultivar “Jane Bell” (119.7 mg). On the other hand, the shortest and narrowest seeds were found in muscadine cultivar “Welder” (4.8 mm) and “Southern Home” (2.7 mm), respectively, and the lowest mean seed weight in cultivar, “Pride” (54.1 mg). The mean berry weight was found to be not corelated with number of seeds/berry (r = 0.1695, *p* = 0.8661), seed length (r = 0.3847, *p* = 0.7023) and seed width (r = 0.2891, *p* = 0.7739), but there was a moderate positive relationship between mean berry weight and seed weight (r = 0.6786) which was statistically nonsignificant (*p* = 0.5010) (Figure 1). However, the higher percentage of seed content (by weight) in berries was found in muscadine grape cultivars “Jane Bell” (7.2% of berry weight) and “Welder” (6.5%) and the lower in “Supreme” (1.9%) and “Black Beauty” (2.1%) cultivars (Table 1).

### 3.2. Identification and Analysis of Muscadine Grape AGL11 Gene

The *AGL11* gene (KM401845) containing 8 exons, was identified in the *Vitis vinifera* genome published at NCBI and Genoscope. The gene is localized on chromosome 18 in bunch grape (*Vitis vinifera*). The overlapping fragments of muscadine grape *AGL11* (*VroAGL11*) gene was amplified, cloned and sequenced from muscadine grape cultivar, Jane Bell. The alignment of *VroAGL11* gene sequence along with the complete coding sequence (mRNA) confirmed the exon-intron structure of the gene predicted at NCBI and ensured that only genomic sequences of the same allele were combined (Figure S1). The *AGL11* gene of the muscadine cultivar was compared with the sequences of bunch grape cultivars, Chardonnay (KM401845) and Sultanina (KM401847). The muscadine *AGL11* gene is 97–98% identical to that of bunch grape cultivars (Chardonnay, Sultanina). 

### 3.3. Detection and Confirmation of VroAGL11 Transcripts

**Figure 2:**
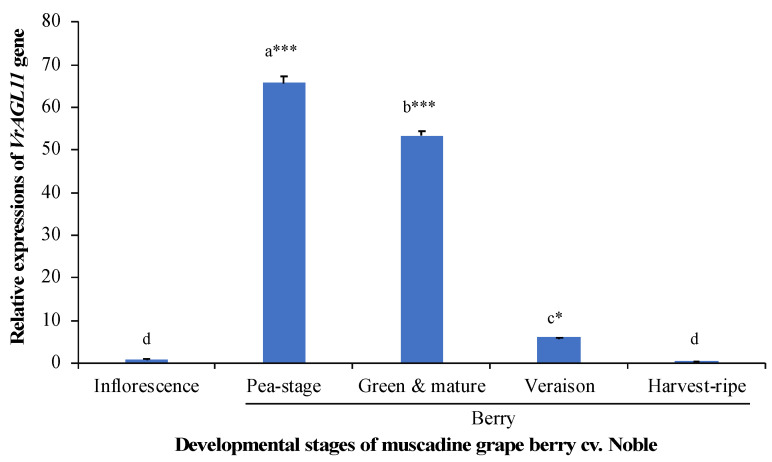
The relative expressions of muscadine grape *AGL11* (*VroAGL11*) gene in leaf, inflorescence and berry at different developmental stages (pea-size, green & mature, veraison and harvest-ripe) of muscadine grape cultivar, Noble. The vertical bars represent the standard error of three biological replicates. Bars with different letters differ significantly according to Tukey HSD post-hoc test (*** *p* < 0.001, * *p* < 0.05).

The *VroAGL11* transcripts were detected in inflorescence and berries at different developmental stages (pea-stage, green & mature, veraison) in muscadine cultivar Noble by qRT-PCR but were hardly detectable in leaves and harvest-ripe berry. However, the highest expressions of *VroAGL11* transcripts were found at pea-stage berry followed by green & mature berry stage which were ~66 and ~53-fold higher respectively compared to that of inflorescence (Figure 2). 

The *VroAGL11* transcripts were amplified from selected seeded and seedless muscadine cultivars (Black Beauty, Fry, Jane Bell, Noble, Fry seedless) with the primers spanning the ORFs, which resulted in amplicons of the expected size (Figure S2) suggesting that the transcripts are conservatively spliced. The absence of smaller as well as larger amplicons in the reactions than the spliced transcripts suggests that *VroAGL11* transcripts were not produced as a result of exon-skipping and intron-retention, respectively. Similar results were found in all seeded and seedless hybrid bunch (BDB) and bunch grape cultivars (Riesling, Reliance seedless) tested. The predicted ORFs were sequenced to confirm the detection of the spliced transcripts.

### 3.4. Analysis of Predicted AGL11 Protein Sequences

**Figure 3:**
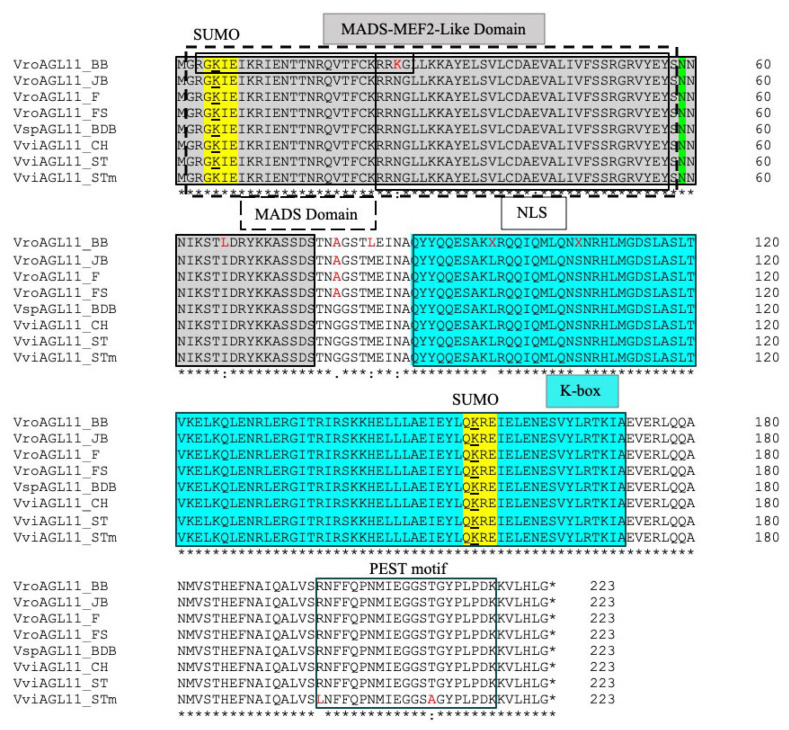
Alignment of the amino acid sequences of all AGL11 proteins of muscadine (cvs. Black Beauty, BB; Jane Bell, JB; Fry, F; Fry Seedless, FS) and hybrid bunch grape (cv. Blanc du Bois, BDB) cultivars with that of published bunch grape cultivars Chardonay (CH: AKJ79177.1) and Sultanina (ST: AKJ79179.1 and mutant allele, STm: AKJ79180.1). Conserved domains, MADS-MEF2-like domain (gray box), MADS domain (dashed line box) and K box (turquoise box), phosphorylation site (N59, highlighted in green), SUMO (sumoylation) and PEST (ubiquitination) motifs, and NLS (nuclear localization signal) sequences are indicated. Red colored amino acids show the amino acid differences between different grape species/cultivars.

The alignment of the amino acid sequences of AGL11 proteins of muscadine grape (cv. Black Beauty, Jane Bell, Fry, Fry Seedless) and hybrid bunch cv. Blanc du Bois (BDB) along with that of published bunch grape cultivars Chardonnay and Sultanina (seeded and seedless allele) showed that they have MADS-MEF2 (myocyte enhancer factor 2)-like domain containing highly conserved MADS domain and K box, characteristic of Type II subfamily of MADS box family of eukaryotic transcriptional regulators (Figure 3). All the AGL11 proteins have putative phosphorylation site (N at 59 position), two sumoylation motifs (GKIE and QKRE at K5 and K155 positions, respectively) and poor PEST motif with a score of −10.49 between the 197 and 217 position. However, the position of the phosphorylation site and sumoylation motifs are conserved like the AGL11 protein of Arabidopsis and other eudicot plants (Figure S3). All the muscadine, bunch and hybrid bunch AGL11 proteins except muscadine cv. Black Beauty have putative bipartite NLS (nuclear localization signal) sequences with a score of 3.9, suggesting that they are localized to both the nucleus and cytoplasm (Figure 3, Table S2). But the Black Beauty AGL11 protein having NLS score of 6.9 is partially localized to nucleus.

The AGL11 proteins predicted for muscadine and hybrid bunch cultivars are 223 amino acids long with a predicted molecular mass of 25.7 kD and isoelectric point (IP) of 8.86, like bunch grape cultivar Chardonnay, with the exception of muscadine cv. Black Beauty and bunch cv. Riesling having IP of 8.91 and 8.95, respectively (Table S2). The AGL11 proteins of muscadine cv. (Jane Bell, Fry and Fry Seedless) and bunch cv. (Chardonnay, Sultanina) differ in only one amino acid, A79G. However, the AGL11 protein of Black Beauty differs from bunch grape cultivars in multiple amino acids in addition to A79G, these are K26N, L66I, L83M, X97L and X107S (Figure 3). However, the AGL11 proteins of all muscadine and bunch grape cultivars differ from protein deduced from Sultanina mutant allele (STm), which is responsible for seedlessness, in two amino acids, R197L and T210A. The AGL11 proteins of muscadine grape cvs. Jane Bell, Fry and Fry Seedless are 99%, Black Beauty 97% and hybrid bunch cv. BDB 100% identical to that of bunch grape cultivars, Chardonnay and Sultanina (ST), but 98, 96 and 99% identical to Sultanina mutant (STm) protein (Table S3). However, muscadine grape cultivars (Black Beauty Jane Bell, Fry and Fry Seedless) are 75–77%, and hybrid bunch (BDB) and bunch grape cultivars (Chardonnay, Sultanina, Reliance Seedless) 77% identical to Arabidopsis AGL11 protein.

### 3.5. Relative Expressions of AGL11 Genes

**Figure 4:**
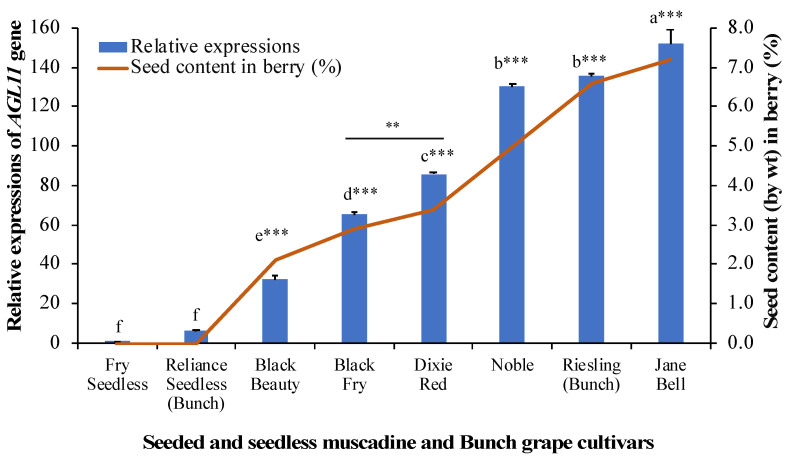
The relative expressions of grape *AGL11* gene in pea-size berries and percentage of seed content (by weight) in berry of selected seeded and seedless muscadine and bunch grape cultivars. The vertical bars represent the standard error of three biological replicates. Bars with different letters differ significantly according to Tukey’s post-hoc test (*** *p* < 0.001, ** *p* < 0.01).

Since the pea-size berries showed the highest expressions of *AGL11* gene, the relative expressions of *AGL11* gene were analyzed by real-time PCR at pea-size berry stage of selected seeded and seedless muscadine and bunch grape cultivars as the pea-size berries had the highest expressions of *AGL11* genes. Seeded muscadine grape cultivars, namely Jane Bell, Noble, Dixie Red, Black Fry and Black Beauty representing different berry sizes were selected along with seedless muscadine cultivar Fry Seedless for the study. Bunch grape cultivar Riesling (seeded) and Reliance seedless were also included in the study. The highest expression of *AGL11* gene at pea-size berry was found in seeded muscadine cultivar “Jane Bell” followed by “Noble” which was ~152- and ~130-fold higher and statistically highly significant (*p* < 0.001) than that of Fry seedless, respectively (Figure 4). However, the lowest expression of *AGL11* gene was found in seedless muscadine cultivar “Fry Seedless” as well as seedless bunch grape cultivar “Reliance Seedless”. 

### 3.6. Relationship between AGL11 Gene Expression Levels and Seed Characteristics

There was little negative but statistically non-significant correlation between the relative expressions of *AGL11* genes and mean berry weight (r = −0.2331, *p* = 0.4047), seed weight/berry (r = −0.3175, *p* = 0.5396), seed length (r = −0.0380) and seed width (r = −0.0349, *p* = 0.9475) of the seeded muscadine and bunch grape cultivars (Figure S4). However, the relative expressions of *AGL11* genes is highly significant and positively corelated (r = 0.9825, *p* < 0.001) with seed content (by weight) in berry (%) of muscadine cultivars irrespective of their berry size (Figure S4). The muscadine cultivar Jane Bell berry having the highest percentage of seed content (by weight) (7.2%) showed the highest *AGL11* gene expression (Figure 4). On the contrary, seedless muscadine (Fry Seedless) and bunch grape (Reliance Seedless) cultivars containing no seed had the lowest *AGL11* gene expression.

### 3.7. Phylogenetic Analysis

**Figure 5:**
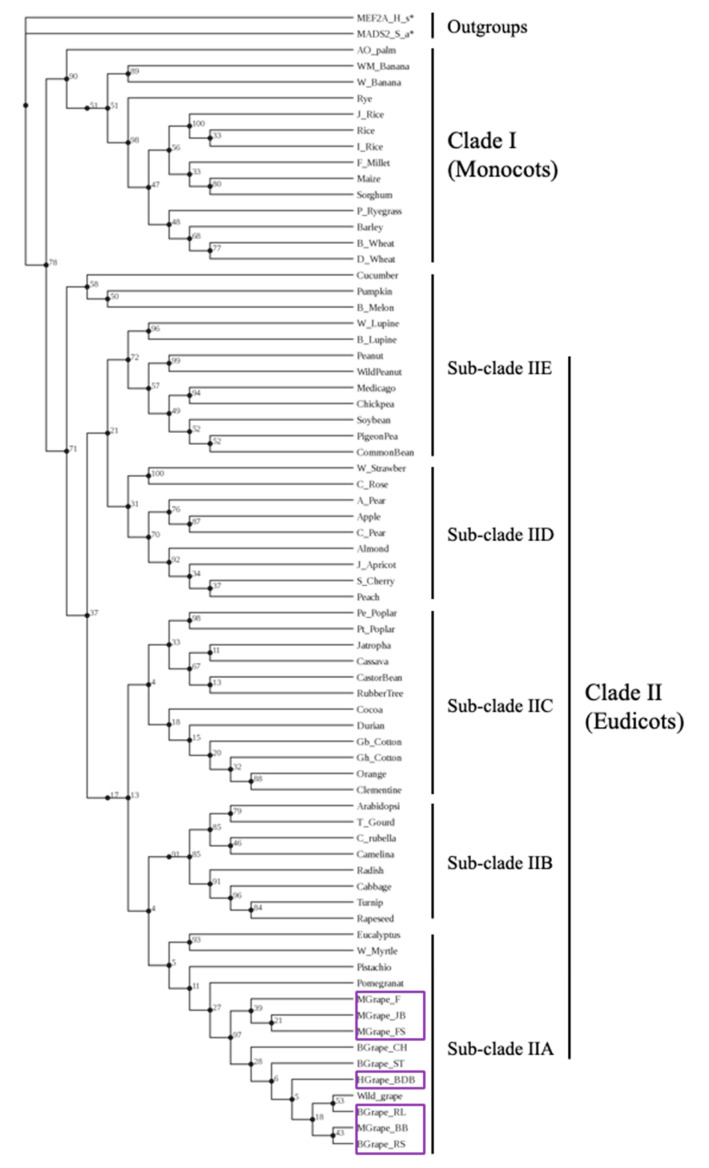
Dendrogram showing the phylogenetic relationship of *AGL11* gene of muscadine grape (*VroAGL11*) with that of other monocot and eudicot plants including bunch, hybrid bunch and wild grape species using MADS-MEF2 transcription factor of fungus *Scedosporium apiospermum* (XP_016645827.1, MADS-MEF2_S.a*) and myocyte-specific enhancer factor 2A of *Homo sapiens* (XP_016877691.1, MEF2A_H.s*) as outgroups. The amino acid sequences of *AGL11* gene of muscadine (BB = Black Beauty, JB = Jane Bell, F = Fry, and FS = Fry Seedless) and hybrid bunch grape (BDB = Blanc du Bois) and bunch (RL = Riesling, RS-Reliance Seedless) sequenced in this study are shown in violet box. The phylogenetic tree was constructed based on amino acid sequences of AGL11 or AGL11-like protein by the PhyML 3.0 [33] using maximum likelihood method with LG (Le and Gascuel) model [34] and bootstrap analysis. The accession numbers of the amino acid sequences of AGL11 or AGL11-like protein of monocots and eudicots are provided in Table S3.

To understand the evolution and divergence of *AGL11* gene, we obtained 61 amino acid sequences of AGL11 or AGL11-like proteins from monocot and eudicots plant species at NCBI database using VvAGL11, AtAGL11 and TaAGL11 protein sequences as query sequences. A maximum likelihood based phylogenetic tree was generated with best-fit model LG using AGL11 protein sequences of these angiospermic plants as well as of 4 grape species (muscadine, hybrid bunch and bunch grape species sequenced and reported in this study), and presented in Figure 5. MADS-MEF2 type transcription factor of fungus *Scedosporium apiospermum* (XP_016645827.1) and myocyte-specific enhancer factor 2A of *Homo sapiens* (XP_016877691.1) were used as outgroups in phylogenetic analysis. AGL11 proteins of all plants formed 2 major clades (clade 1 and clade II) based on the tree topology with a well-supported bootstrap value (78%) consisting of monocots (14 species) and eudicots (51 species), respectively. However, the clade II consisting of eudicot species was again subdivided into 5 sub-clades. All the grape species including muscadine, hybrid bunch, bunch and wild grape species along with Pomegranate, Wild Myrtle and Eucalyptus form a distinctly separate sub-clade (IIA). Fruit trees of the family Rosaceae including stone fruit, pomes, drupe and drupelet fruits formed a sub-clade (IID). The members of the Brassicaceae including Arabidopsis, and Leguminosae family formed clades IIB and IIE, respectively. Other tree plants including Orange, Cotton, Rubber and Poplar tree formed a sub-clade IIC suggesting the divergence of the *AGL11* gene among the eudicot species.

## 4. Discussion

Seedlessness is the most desirable trait in the muscadine breeding program. This study was undertaken to identify and characterize the *AGL11* gene for stenospermocarpic seedlessness in muscadine grape at the molecular level, determine the relationship of its expression levels with percentage of seed content (by weight) in berry of selected seeded and seedless muscadine and hybrid bunch/bunch grape cultivars, and analyze the divergence of muscadine *AGL11* gene from that of other eudicot and monocot plants with an aim to develop seedless muscadine table grape. 

Muscadine berries were of different weight depending on the cultivar, with the biggest berry in Black Beauty (15.9 g) and the smallest in Fry Seedless (2.5 g) cultivars (Table 1). The number of seeds in muscadine berries, however, varies from 2 to 5 per berry depending on the cultivar, and had little relationship with berry weight (r = 0.1695, *p* > 0.05) i.e., the berry seed number does not depend on their berry weight (Figure 1). Similarly, seed sizes of the muscadine berries varied from cultivar to cultivar ranging from 4.8 to 7.9 mm in length and 2.5 to 5.6 mm in width but had minimal relationship with their berry weight (seed length: r = 0.3847, width: r = 0.2891; *p* > 0.05). Interestingly the mean berry weight and seed weight/berry were moderately and positively correlated (r = 0.6786, *p* > 0.05) which was, however, statistically nonsignificant (Figure 1). As the muscadine berries and their seeds are of different sizes and weights depending on the cultivar, we have calculated the seed: berry ratio (by weight) in percentage to ascertain the percentage of berry weight belonging to the seed content among different grape cultivars. The highest percentage of seed content (by weight) in berry was found in muscadine cv. Jane Bell (7.2% of berry weight) and the lowest in Supreme (1.9%) and Black Beauty (2.1%) (Table 1).

The MADS-box transcription factor gene *AGL11* of bunch grape (*VviAGL11*) has been proposed as a strong candidate gene for seed morphogenesis in bunch grape and later reported as the source of origin of stenospermocarpic seedlessness in bunch grape cultivars [8,17,22,25,26]. Recently Malabarba and her colleagues [27] confirmed its role as master regulator of seed morphogenesis in bunch grapes and showed that silencing and overexpressing of the *VviAGL11* gene decrease and increase the seed content in seeded and seedless bunch grape cultivars, respectively. However, the *AGL11* gene has never been characterized in the muscadine grapes at the molecular level. 

As the muscadine grape genome is not sequenced yet, we detected the *AGL11* transcripts in muscadine grapes by qRT-PCR using gene-specific primers designed from the bunch grape *VviAGL11* gene and validated by amplifying and sequencing the coding sequence (mRNA) from seeded and seedless muscadine cultivars. However, the muscadine grape *AGL11* transcripts (*VroAGL11*) were highly expressed in berries especially in pea-size, and green and mature berries showing ~66 and ~53 times more expression respectively than inflorescence (Figure 2), but barely detectable in leaves and harvest-ripe berries of muscadine cultivar Noble. Díaz-Riquelme et al. [20] also reported higher expression of *AGL11* gene in floral and fruit tissues of bunch grapes concurring our observation. The expression of *VviAGL11* was 25-fold higher in pea-size berries compared to flowers in bunch grape [8].

The expected size of resulting amplicons of the *AGL11* transcripts from different muscadine, hybrid bunch and bunch grape cultivars using primers spanning the ORFs suggests that they were conservatively spliced and might not be produced by exon-skipping or intron-retention (Figure S2) and later confirmed by sequencing their ORFs. The alignment of *AGL11* genomic and coding (mRNA) sequences of muscadine grape cv. Jane Bell along with the published genomic and coding sequences of *AGL11* gene of bunch grape cvs. Chardonnay and Sultanina confirmed the exon-intron structure predicted at NCBI (Figure S1). The muscadine grape *VroAGL11* gene also contains 8 exons like published bunch grape *VviAGL11* gene (Chardonnay and Sultanina). All the predicted muscadine grape VroAGL11 proteins, like that of bunch grape and Arabidopsis, contain highly conserved MADS-MEF2-like domain containing MADS domain, and K box (Figure 3). They also have putative phosphorylation site (N59), two sumoylation motifs (GKIE and QKRE at K5 and K155) and a poor ubiquitination motif for posttranslational modification. The 223 amino acid long VroAGL11 proteins of muscadine grape cultivars, Jane Bell, Fry and Fry Seedless are almost identical (99%) to that of bunch grape (Chardonnay) except in one amino acid (A79G), but the Black Beauty VroAGL11 protein was 97% identical to bunch grape (Chardonnay) and differs in five more amino acids in addition to A79G (K26N, L66I, L83M, X97L and X107S). Interestingly the AGL11 protein of hybrid bunch grape cultivar, BDB was 100% identical to bunch grape cultivar Chardonnay. However, the AGL11 proteins of all muscadine and hybrid bunch grape cultivar differ from mutant protein (deduced from mutant allele) of Sultanina cultivar, which is used as the donor parent for introducing stenospermocarpic seedlessness in table grape breeding program, in two amino acids, R197L and T210A (Figure 3). Royo et al. [26] reported that the arginine-197-to-leucine substitution in VviAGL11 was linked with stenospermocarpy and major cause of seedlessness in bunch grapes.

We quantified relative expressions of the *AGL11* gene in pea size berries of the selected seeded and seedless muscadine, hybrid bunch and bunch grape cultivars as the pea-size berries had the highest expressions among the plant parts. All the seeded grape cultivars had the higher expression of *AGL11* gene compared to seedless cultivar irrespective of cultivars. However, the seeded muscadine cultivar Jane Bell and Noble had the higher expression of *AGL11* gene which was 152- and 130-fold higher than that of Fry Seedless (Figure 4). Interestingly, there was highly significant and near-perfect positive relationship (r = 0.9825, *p* < 0.001) between *AGL11* gene expressions and percentage of seed content (by weight) in berry, i.e., the higher the percentage of seed content (by weight) in berry, the higher the expression of *AGL11* gene (Figure 4). Muscadine cultivar Jane Bell showing the highest expression of *AGL11* gene had the highest percentage of seed content (7.2% of berry weight) (Figure 4, Table 1). On the other hand, Black Beauty and Fry Seedless showing little to almost no expression of *AGL11* gene had lower (2.1%) or no seed content, respectively. This finding suggest that *VroAGL11* gene also controls the stenospermocarpic seedlessness trait in muscadine grape like in bunch grape. Interestingly, it should be noted that muscadine cultivar Fry Seedless, which is parthenocarpic, showed lower expression of *AGL11* gene which is linked to stenospermocarpy. Even though the mechanism or process of producing seedless berry is different in Fry Seedless cultivar, it showed minimum or little expression of *VroAGL11* gene since *AGL11* transcripts are accumulated in the dual endotesta layer of the seeds [22] and Fry Seedless has only seed traces or no seeds.

Phylogenetic analysis of *AGL11* genes showed the divergence of the *AGL11* genes among the angiospermic plants forming two major clades, clade 1 and clade II consisting of monocot and eudicots, respectively with well-supported bootstrap values (Figure 5). The *AGL11* genes of 51 eudicot species composing clade II again formed five sub-clades. The sub-clades 2B, 2D and 2E predominately represent the members of Brassicaceae, Rosaceae and Leguminosae family, respectively. All the tree plants including Orange, Cotton, Rubber and Poplar tree formed a separate sub-clade 2C. These data suggest that Muscadine grape cultivars along with all other grape species including bunch, hybrid bunch, and wild grapes form a distinct separate sub-clade (2A) proving that the *AGL11* gene is phylogenetically similar and highly conserved in all grape species.

## 5. Conclusions

The current study identified and characterized the *VviAGL11* orthologous gene in muscadine grapes (*VroAGL11*) at the molecular level. The *AGL11* gene is conserved across all the grape species and phylogenetically they formed a separate clade than that of other eudicots and monocots. The highest expression of *VroAGL11* gene found in muscadine berry with the highest percentage of seed content (by weight) and almost no expression in seedless berry suggest that *VroAGL11* gene plays a major role in controlling seed morphogenesis in muscadine grapes. 

## Figures and Tables

**Table 1:** Berry and seed characteristics of muscadine, hybrid bunch and bunch grape cultivars grown in vineyard at Center for Viticulture and Small Fruit Research.

Serial Number	Name of Cultivars	Seeded/Seedless ^1^	Mean Berry Weight (=BW/BN) g	Mean Seed Number /Berry (=SN/BN)	Mean Seed Weight /Berry (=SW/BN) mg	Mean Seed Weight (=SW/SN) mg	Seed Length (mm) (Mean ± SE)	Seed Width (mm) (Mean ± SE)	Seed Content of Berry (%) =(SW/BW) * 100
	**Muscadine grapes**								
1	African Queen	S	9.9	3.3	280.6	85.0	5.7 ± 0.18	3.9 ± 0.16	2.8
2	Alachua	S	8.9	4.1	349.8	85.3	6.3 ± 0.12	3.8 ± 0.12	3.9
3	Albermale	S	6.1	4.2	318.2	76.4	5.7 ± 0.10	3.0 ± 0.10	5.2
4	Black Beauty	S	15.9	3.8	332.8	86.8	6.0 ± 0.11	3.6 ± 0.10	2.1
5	Black Fry	S	10.5	3.5	307.8	88.0	6.2 ± 0.20	3.7 ± 0.17	2.9
6	Carlos	S	5.0	3.8	272.3	71.0	7.3 ± 0.06	4.8 ± 0.06	5.4
7	Cowart	S	7.1	4.0	366.7	91.7	6.0 ± 0.29	3.6 ± 0.11	5.2
8	Darlene	S	15.1	3.8	373.9	99.7	5.9 ± 0.10	3.8 ± 0.08	2.5
9	Dixie	S	5.1	3.7	251.8	68.7	6.7 ± 0.06	4.8 ± 0.15	5.0
10	Dixie Red	S	9.3	3.9	315.8	81.0	6.4 ± 0.09	3.8 ± 0.08	3.4
11	Doreen	S	10.7	3.5	354.5	101.3	6.2 ± 0.09	3.8 ± 0.08	3.3
12	Farrer	S	11.2	3.0	344.0	114.7	7.0 ± 0.13	3.9 ± 0.08	3.1
13	Fry	S	10.2	2.6	244.6	94.7	5.9 ± 0.08	3.8 ± 0.10	2.4
14	Fry Seedless	SL	2.5						
15	Granny Val	S	11.7	3.1	298.3	96.8	7.0 ± 0.06	3.9 ± 0.18	2.6
16	Higgins	S	6.0	4.1	275.5	67.5	6.8 ± 0.18	4.6 ± 0.05	4.6
17	Hunt	S	5.5	4.2	268.5	63.9	5.5 ± 0.05	3.0 ± 0.06	4.9
18	Ison	S	6.7	3.8	332.0	86.6	7.9 ± 0.08	5.0 ± 0.07	5.0
19	Jane Bell	S	5.3	3.2	383.0	119.7	7.2 ± 0.08	4.1 ± 0.11	7.2
20	Janet	S	15.5	4.9	578.9	118.1	6.8 ± 0.06	3.9 ± 0.10	3.7
21	Jumbo	S	10.5	4.4	409.3	92.7	6.4 ± 0.07	3.4 ± 0.08	3.9
22	Late Fry	S	14.7	3.8	415.9	108.5	7.9 ± 0.06	5.6 ± 0.14	2.8
23	Loomis	S	10.2	3.4	319.1	93.4	7.4 ± 0.07	5.2 ± 0.05	3.1
24	Magnolia	S	7.8	3.7	219.8	59.9	7.5 ± 0.11	4.9 ± 0.09	2.8
25	Nesbitt	S	10.1	3.5	327.2	93.5	6.3 ± 0.08	3.7 ± 0.12	3.2
26	Noble	S	4.7	4.0	235.3	58.8	6.4 ± 0.05	4.6 ± 0.04	5.0
27	Pam	S	14.5	3.3	326.6	98.0	6.2 ± 0.16	3.8 ± 0.11	2.3
28	Pineapple	S	12.2	3.5	369.2	105.5	6.2 ± 0.08	3.9 ± 0.12	3.0
29	Pride	S	4.3	3.8	202.9	54.1	4.8 ± 0.04	3.1 ± 0.03	4.7
30	Regale	S	5.1	3.8	259.8	69.3	5.5 ± 0.05	3.2 ± 0.07	5.1
31	Scarlet	S	12.1	2.7	261.2	96.7	6.7 ± 0.14	4.0 ± 0.15	2.2
32	Scuppernong	S	10.2	2.3	215.7	92.4	6.2 ± 0.08	3.4 ± 0.15	2.1
33	Southern Home	S	6.3	3.4	204.2	59.8	5.9 ± 0.12	2.7 ± 0.04	3.3
34	Southern Land	S	6.8	3.7	280.8	76.6	6.0 ± 0.07	3.5 ± 0.11	4.2
35	Sterling	S	8.8	3.9	333.2	85.1	6.1 ± 0.11	3.7 ± 0.08	3.8
36	Sugar Pop	S	10.5	3.9	391.3	99.9	6.2 ± 0.11	3.8 ± 0.06	3.7
37	Summit	S	10.7	3.0	242.8	80.9	6.1 ± 0.06	3.2 ± 0.08	2.3
38	Supreme	S	15.5	2.8	300.0	105.9	6.3 ± 0.07	4.0 ± 0.09	1.9
39	Sweet Jenny	S	11.8	4.1	379.8	93.0	7.5 ± 0.10	5.2 ± 0.13	3.2
40	Triumph	S	9.8	3.8	249.3	66.5	6.0 ± 0.07	3.5 ± 0.09	2.5
41	Watergate	S	8.7	3.2	229.9	72.6	7.3 ± 0.15	4.9 ± 0.10	2.6
42	Welder	S	3.3	3.3	217.2	65.2	4.8 ± 0.04	3.5 ± 0.10	6.5
**Florida hybrid bunch**									
43	Blanc du Bois	S	1.7	1.9	55.1	28.3			3.2
44	Stover	S	2.0	1.3	61.9	48.5	5.1 ± 0.12	2.7 ± 0.06	3.1
**Bunch grape**									
45	Riesling	S	1.6	3.6	107.9	30.1	4.0 ± 0.13	2.0 ± 0.10	6.6

^1^ Note: S = seeded, SL = seedless, BW = berry weight, BN = berry number, SN = seed number, SW = seed. weight and SE = standard error.

## Data Availability

All relevant data are included in the manuscript and supplementary files.

## Supplementary Materials

The following are available online at https://www.mdpi.com/2073-4425/12/2/232/s1, Table S1: List of primers used for detection, amplification and sequencing of Muscadine, bunch and hybrid bunch grape *AGL11* gene and transcripts. Table S2: Properties of AGL11 protein of muscadine, hybrid bunch and bunch grape cultivars. Table S3: Percent identity matrix of AGL11 or AGL11-like proteins of monocot and Eudicot plants. Figure S1: Multiple alignment of the genomic sequences of AGL11 gene of muscadine (cv. Jane Bell) and bunch grape (cvs. Chardonnay, Sultanina) cultivars along with their corresponding mRNAs. Figure S2: Gel picture showing the size of conservatively spliced transcripts (740 bp) of *AGL11* gene in muscadine cultivars Jane Bell, Noble, Black Beauty and Fry Seedless. Figure S3: Multiple alignment of the amino acid sequences of AGL11 or AGL11-like proteins of monocot and eudicot plants including muscadine, hybrid bunch and bunch grape cultivars sequenced and reported in this study. Figure S4: Scatter plots showing relationship between relative expression of *AGL11* gene of selected muscadine and bunch grape cultivars and (a) mean berry weight; (b) seed weight/Berry; (c) seed length; (d) seed width.

## References

[1] FWGGA Florida Wine and Grape Growers Association. https://www.fgga.org/.

[2] Balasubramani S.P., Rahman M.A., Basha S.M. (2019). Synergistic Action of Stilbenes in Muscadine Grape Berry Extract Shows Better Cytotoxic Potential Against Cancer Cells Than Resveratrol Alone. Biomedicines.

[3] Li R., Kim M.H., Sandhu A.K., Gao C., Gu L. (2017). Muscadine Grape (*Vitis rotundifolia*) or Wine Phyto-chemicals Reduce Intestinal Inflammation in Mice with Dextran Sulfate Sodium-Induced Colitis. J. Agric. Food Chem..

[4] Mendonca P., Darwish A.G., Tsolova V., El-Sharkawy I., Soliman K.F.A. (2019). The Anticancer and Antioxidant Effects of Muscadine Grape Extracts on Racially Different Triple-negative Breast Cancer Cells. Anticancer Res..

[5] Kovaleva L.V., Smirnova N.K., Milyaeva E.L. (1997). Seedlessness: Structure and Metabolic activity of *Vitis vinifera* L. female gametophyte (cv. Kishmish Chernyi). Russ. J. Plant Physiol..

[6] Ledbetter C.A., Ramming D.W. (1989). Seedlessness in Grapes. Horticultural Reviews.

[7] Bouquet A., Danglot Y. (1996). Inheritance of seedlessness in grapevine (*Vitis vinifera* L.). Vitis.

[8] Mejía N., Soto B., Guerrero M., Casanueva X., Houel C., de los Ángeles Miccono M., Ramos R., Le Cunff L., Boursiquot J.-M., Hinrichsen P. (2011). Molecular, genetic and transcriptional evidence for a role of VvAGL11 in stenospermocarpic seedlessness in grapevine. BMC Plant Biol..

[9] Basiouny F.M., Himelrick D.G. (2001). Muscadine Grapes.

[10] Adam-Blondon A.F., Lahogue-Esnault F., Bouquet A., Boursiquot J.M., This P. (2001). Usefulness of two SCAR markers for marker-assisted selection of seedless grapevine cultivars. Vitis.

[11] Dangl G.S., Mendum M.L., Prins B.H., Walker M.A., Meredith C.P., Simon C.J. (2001). Simple sequence repeat analysis of a clonally propagated species: A tool for managing a grape germplasm collection. Genome.

[12] Ibáñez J., Vargas A.M., Palancar M., Borrego J., de Andrés M.T. (2009). Genetic relationships among table-grape varieties. Am. J. Enol. Vitic..

[13] Ibáñez J., Carreño J., Yuste J., Martínez-Zapater J.M., Reynolds A.G. (2015). Grapevine breeding and clonal selection programmes in Spain. Grapevine Breeding Programs for the Wine Industry.

[14] Di Genova A., Almeida A.M., Munoz-Espinoza C., Vizoso P., Travisany D., Moraga C., Pinto M., Hinrichsen P., Orellana A., Maass A. (2014). Whole genome comparison between table and wine grapes reveals a comprehensive catalog of structural variants. BMC Plant Biol..

[15] Lahogue F., This P., Bouquet A. (1998). Identification of a codominant scar marker linked to the seedlessness character in grapevine. Theor. Appl. Genet..

[16] Cabezas J.A., Cervera M.T., Ruiz-García L., Carreño J., Martínez-Zapater J.M. (2006). A genetic analysis of seed and berry weight in grapevine. Genome.

[17] Costantini L., Battilana J., Lamaj F., Fanizza G., Grando M.S. (2008). Berry and phenology-related traits in grapevine (*Vitis vinifera* L.): From Quantitative Trait Loci to underlying genes. BMC Plant Biol..

[18] Doligez A., Bouquet A., Danglot Y., Lahogue F., Riaz S., Meredith C., Edwards K., This P. (2002). Genetic mapping of grapevine (*Vitis vinifera* L.) applied to the detection of QTLs for seedlessness and berry weight. Theor. Appl. Genet..

[19] Mejía N., Gebauer M., Muñoz L., Hewstone N., Muñoz C., Hinrichsen P. (2007). Identification of QTLs for Seedlessness, Berry Size, and Ripening Date in a Seedless × Seedless Table Grape Progeny. Am. J. Enol. Vitic..

[20] Diaz-Riquelme J., Grimplet J., Martínez-Zapater J.M., Carmona M.J. (2012). Transcriptome variation along bud development in grapevine (*Vitis vinifera* L.). BMC Plant Biol..

[21] Favaro R., Pinyopich A., Battaglia R., Kooiker M., Borghi L., Ditta G., Yanofsky M., Kater M., Colobo L. (2003). MADS-Box Protein Complexes Control Carpel and Ovule Development in Arabidopsis. Plant Cell.

[22] Malabarba J., Buffon V., Mariath J.E.A., Gaeta M.L., Dornelas M.C., Margis-Pinheiro M., Pasquali G., Revers L.F. (2017). The MADS-box gene Agamous-like 11 is essential for seed morphogenesis in grapevine. J. Exp. Bot..

[23] Mizzotti C., Ezquer I., Paolo D., Rueda-Romero P., Guerra R.F., Battaglia R., Rogachev I., Aharoni A., Kater M.M., Caporali E. (2014). SEEDSTICK is a Master Regulator of Development and Metabolism in the Arabidopsis Seed Coat. PLoS Genet..

[24] Pinyopich A., Ditta G.S., Savidge B., Liljegren S.J., Baumann E., Wisman E., Yanofsky M.F. (2003). Assessing the redundancy of MADS-box genes during carpel and ovule development. Nature.

[25] Ocarez N., Mejía N. (2016). Suppression of the D-class MADS-box AGL11 gene triggers seedlessness in fleshy fruits. Plant Cell Rep..

[26] Royo C., Torres-Pérez R., Mauri N., Diestro N., Cabezas J.A., Marchal C., Lacombe T., Ibáñez J., Tornel M., Carreño J. (2018). The Major Origin of Seedless Grapes Is Associated with a Missense Mutation in the MADS-Box Gene *VviAGL11*. Plant Physiol..

[27] Malabarba J., Buffon V., Mariath J.E.A., Maraschin F.S., Margis-Pinheiro M., Pasquali G., Revers L.F. (2018). Manipulation of *VviAGL11* Expression Changes the Seed Content in Grapevine (*Vitis vinifera* L.). Plant Sci..

[28] Coombe B.G. (1995). Growth Stages of the Grapevine: Adoption of a System for Identifying Grapevine Growth Stages. Aust. J. Grape Wine Res..

[29] Rahman M.A., Moody M.A., Nassuth A. (2014). Grape contains 4 ICE genes whose expression includes alternative polyadenylation, leading to transcripts encoding at least 7 different ICE proteins. Environ. Exp. Bot..

[30] Livak K.J., Schmittgen T.D. (2001). Analysis of Relative Gene Expression Data Using Real-Time Quantitative PCR and the 2−ΔΔCT Method. Methods.

[31] Kim C.S., Lee C.H., Shin J.S., Chung Y.S., Hyung N.I. (1997). A Simple and Rapid Method for Isolation of High Quality Genomic DNA from Fruit Trees and Conifers Using PVP. Nucleic Acids Res..

[32] Kosugi S., Hasebe M., Tomita M., Yanagawa H. (2009). Systematic Identification of Cell Cycle-Dependent Yeast Nucleocytoplasmic Shuttling Proteins by Prediction of Composite Motifs. Proc. Natl. Acad. Sci. USA.

[33] Guindon S., Dufayard J.-F., Lefort V., Anisimova M., Hordijk W., Gascuel O. (2010). New Algorithms and Methods to Estimate Maximum-Likelihood Phylogenies: Assessing the Performance of PhyML 3.0. Syst. Biol..

[34] Le S.Q., Gascuel O. (2008). An Improved General Amino Acid Replacement Matrix. Mol. Biol. Evol..

[35] Lefort V., Longueville J.-E., Gascuel O. (2017). SMS: Smart Model Selection in PhyML. Mol. Biol. Evol..

[36] R Core Team (2019). R: A Language and Environment for Statistical Computing.

