# Supplementary material for: Molecular Characterization and Phylogenetic Analysis of MADS-Box Gene VroAGL11 Associated with Stenospermocarpic Seedlessness in Muscadine Grapes
Source: Genes (Basel). 2021 Feb 5;12(2):232. doi: 10.3390/genes12020232 (PMC7915462; doi:10.3390/genes12020232)
Supplement: Supplementary file 1 [file genes-12-00232-s001.pdf]

Table S1: List of primers used for detection, amplification and sequencing of Muscadine, Bunch and hybrid Bunch grape *AGL11* gene and transcripts.

Primer pair#	Primer name	Primer Sequence (5' – 3')*	Purpose of use
1	VvAGL11-F	GCATGAGTTGCTGTTGGCTG	To detect *AGL11* transcripts
	VvAGL11-R	TGCTGAAGCCTCTCCACTTC	
2	VvActin-F	TGGTATTGTGCTGGATTCTGGTG	Reference gene, grape *Actin* for normalization (Shi et al., 2014)*
	VvActin-R	AGATGGCTGGAAGAGGACTTCTG	
3	AGL11-ORF1-1179F	TTGGCTTCATTGGGATACGC	To amplify and sequence *AGL11* coding region (nested PCR Ist pair)
	AGL11-ORF1-1180R	AACAGTTTGGATAGCATGGGA	
4	AGL11-ORFn-1083F	GTCGCCGGATTTTGGAAACA	To amplify and sequence *AGL11* coding region (nested PCR 2nd pair)
	AGL11-ORFn-1083R	AAGGAGATGAAGTTGGCGGA	
5	AGL11-ORF1-1179F	TCACCCTCTACCAAAAACACACA	To amplify and sequence *AGL11* gene (Ist pair)
	VviAGL11- R1	AGATTCATCAGCAGATGCCAGA	
6	VviAGL11- F2	AAGGGATTACATGGGTACCTGC	To amplify and sequence *AGL11* gene (2nd pair)
	VviAGL11- R2	ACAAGGGAGAAATCAAGGAGGAA	
7	VviAGL11- F3	GCACCCATTACATCTTTGTGTGG	To amplify and sequence *AGL11* gene (3rd pair)
	VviAGL11- R3	TCATTGGAGCATCGTCTTAGAGT	
8	VviAGL11- F4	GCAACCCCTACACTTTACACC	To amplify and sequence *AGL11* gene (4th pair)
	VviAGL11- R4	TTGAGCTTGAAGGGGGTTCG	
9	VviAGL11- F5	ATGGAAACCCAAATCAAATGTTGC	To amplify and sequence *AGL11* gene (5th pair)
	VviAGL11- R5	TGGTTTCTCCTCTCCAGCAC	
11	VviAGL11- F6	TCTGAACCATCTGGCAGTGTC	To amplify and sequence *AGL11* gene (6th pair)
	VviAGL11- R6	AGACACAAGCAGAAGGAAAATGC	
13	VviAGL11- F7	ACCAGCAAGAATCAGCAAAGC	To amplify and sequence *AGL11* gene (7th pair)
	VvAGL11-R	TGCTGAAGCCTCTCCACTTC	
14	VviAGL11- F8	GCATGAGTTGCTGTTGGCTG	To amplify and sequence *AGL11* gene (8th pair)
	AGL11-ORFn-1083R	AAGGAGATGAAGTTGGCGGA	

*Shi, J.; He, M.; Cao, J.; Wang, H.; Ding, J.; Jiao, Y.; Li, R.; He, J.; Wang, D.; Wang, Y. The Comparative Analysis of the Potential Relationship between Resveratrol and Stilbene Synthase Gene Family in the Development Stages of Grapes (*Vitis quinquangularis* and *Vitis vinifera*). *Plant Physiology and Biochemistry* **2014**, *74*, 24–32. https://doi.org/10.1016/j.plaphy.2013.10.021.

Table S2: P Properties of AGL11 protein of muscadine, hybrid Bunch and Bunch grape cultivars

AGL11 protein*	Protein Mass (kD)	#Amino acids	Isoelectric point	PEST motif		SUMOplot score		Predicted bipartite NLS (Nuclear localization signal)	
				Sequence (position: 197-217)	Score	K5 (GKIE)	K155 (QKRE)	Sequence (Position: 24-57)	Score
Sequenced and reported in this study									
VroAGL11_JB	25.7	223	8.86	RNFFQPNMIEGGSTGYPLPDK	-10.49	0.67	0.5	RRNGLLKKAYELSVLCDAEVALIVFSSRGRVYEY	3.9
VroAGL11_F	25.7	223	8.86	RNFFQPNMIEGGSTGYPLPDK	-10.49	0.67	0.5	RRNGLLKKAYELSVLCDAEVALIVFSSRGRVYEY	3.9
VroAGL11_BB	25.7	223	8.91	RNFFQPNMIEGGSTGYPLPDK	-10.49	0.67	0.5	3 RGKIEIKRIENTTNRQVTFCKRRKG<br>24 RRKGLLKKAYELSVLCDAEVALIVFSSRGRVYEY	6.9<br>4.1
VroAGL11_FS	25.7	223	8.86	RNFFQPNMIEGGSTGYPLPDK	-10.49	0.67	0.5	24 RRNGLLKKAYELSVLCDAEVALIVFSSRGRVYEY	3.9
VspAGL11_BDB	25.7	223	8.86	RNFFQPNMIEGGSTGYPLPDK	-10.49	0.67	0.5	RRNGLLKKAYELSVLCDAEVALIVFSSRGRVYEY	3.9
VviAGL11_RL	25.7	223	8.95	RNFFQPNMIEGGSTGYPLPDK	-10.49	0.67	0.5	RRNGLLKKAYELSVLCDAQVALIVFSSRGRVYEY	3.9
VlaAGL11_RS	25.7	223	8.86	RNFFQPNMIEGGSTGYPLPDK	-10.49	0.67	0.5	RRNGLLKKAYELSVLCDAEVALIVFSSRGRVYEY	3.7
Published at NCBI									
VviAGL11_CH	25.7	223	8.86	RNFFQPNMIEGGSTGYPLPDK	-10.49	0.67	0.5	RRNGLLKKAYELSVLCDAEVALIVFSSRGRVYEY	3.9
VviAGL11_ST	25.7	223	8.86	RNFFQPNMIEGGSTGYPLPDK	-10.49	0.67	0.5	RRNGLLKKAYELSVLCDAEVALIVFSSRGRVYEY	3.9
VviAGL11_STm	25.6	223	8.76	186 HEFNAIQALVSLNFFQPN<br>MIEGGSAGYPLPDK 217	-14.76	0.67	0.5	RRNGLLKKAYELSVLCDAEVALIVFSSRGRVYEY	3.9

* JB= Jane Bell, F=Fry, BB=Black Beauty, FS= Fry Seedless, BDB=Blanc du Bois, RL= Riesling, RS= Reliance Seedless, CH= Chardonnay, ST= Sultanina and STm= Sultanina (mutant)

Table S3: Percent identity matrix of AGL11 or AGL11-like proteins of monocot and eudicot plants (created by Clustal 2.1)

		1	2	3	4	5	6	7	8	9	10	11	12	13	14	15	16	17	18	19	20	21	22	23
**1**	W_Strawberry (XP_011461176.1)	100.0	96.9	85.5	84.6	83.3	83.3	84.2	84.6	83.7	83.7	84.2	86.4	87.3	86.8	86.8	82.8	81.9	81.5	81.9	79.6	84.1	83.8	84.2
**2**	C_Rose (XP_024169047.1)	96.9	100.0	84.6	83.7	82.0	82.0	82.8	83.3	82.4	81.9	82.4	85.5	86.4	85.9	85.9	82.8	81.9	81.5	81.4	78.7	83.2	82.9	82.9
**3**	W_Lupine (KAF1898823.1)	85.5	84.6	100.0	96.1	91.4	90.6	90.9	91.4	91.4	91.8	91.0	88.2	88.7	88.2	88.7	83.3	84.2	82.9	82.4	80.2	86.8	82.4	86.9
**4**	B_Lupine (XP_019465480.1)	84.6	83.7	96.1	100.0	91.4	91.4	90.9	90.9	91.4	91.4	90.5	87.8	88.2	87.8	88.2	83.8	83.8	82.4	81.8	79.1	86.4	81.9	87.8
**5**	WildPeanut (XP_016204599.1)	83.3	82.0	91.4	91.4	100.0	100.0	94.6	93.7	94.6	92.3	91.5	88.7	89.1	88.7	89.1	84.3	85.7	84.3	81.0	81.2	84.7	83.4	84.8
**6**	Peanut (XP_025657296.1)	83.3	82.0	90.6	91.4	100.0	100.0	94.6	93.7	94.6	92.3	91.5	88.7	89.1	88.7	89.1	84.3	85.7	84.3	77.8	81.2	84.7	83.4	84.8
**7**	Soybean (NP_001236130.1)	84.2	82.8	90.9	90.9	94.6	94.6	100.0	97.3	98.2	95.1	94.6	89.0	89.0	89.0	89.5	84.6	85.9	84.6	83.6	83.6	88.1	83.3	86.4
**8**	PigeonPea (XP_020240314.1)	84.6	83.3	91.4	90.9	93.7	93.7	97.3	100.0	99.1	96.0	95.5	90.0	90.0	90.0	90.4	85.0	86.4	85.0	84.1	84.6	88.1	83.3	87.3
**9**	CommonBean (XP_007148609.1)	83.7	82.4	91.4	91.4	94.6	94.6	98.2	99.1	100.0	96.0	95.5	90.0	90.0	90.0	90.4	85.0	86.4	85.0	83.6	83.6	87.2	82.8	87.8
**10**	Chickpea (XP_004499566.1)	83.7	81.9	91.8	91.4	92.3	92.3	95.1	96.0	96.0	100.0	98.7	88.1	88.1	88.1	88.6	83.6	85.0	83.6	83.2	83.2	86.8	82.4	87.3
**11**	Medicago (XP_003598035.1)	84.2	82.4	91.0	90.5	91.5	91.5	94.6	95.5	95.5	98.7	100.0	88.2	88.2	88.6	88.6	82.8	84.2	83.7	84.1	83.7	86.8	81.9	86.9
**12**	Peach (XP_007223946.1)	86.4	85.5	88.2	87.8	88.7	88.7	89.0	90.0	90.0	88.1	88.2	100.0	99.1	99.1	99.6	91.0	92.3	93.2	85.5	83.2	87.7	81.8	87.8
**13**	J_Apricot (XP_008219059.1)	87.3	86.4	88.7	88.2	89.1	89.1	89.0	90.0	90.0	88.1	88.2	99.1	100.0	99.1	99.6	91.0	91.4	92.3	85.5	83.6	88.1	82.7	86.9
**14**	Almond (XP_034222093.1)	86.8	85.9	88.2	87.8	88.7	88.7	89.0	90.0	90.0	88.1	88.6	99.1	99.1	100.0	99.6	91.0	91.4	92.3	85.9	83.6	88.1	82.3	87.3
**15**	S_Cherry (XP_021831176.1)	86.8	85.9	88.7	88.2	89.1	89.1	89.5	90.4	90.4	88.6	88.6	99.6	99.6	99.6	100.0	91.4	91.9	92.8	85.9	83.6	88.1	82.3	87.3
**16**	A_Pear (AJW29026.1)	82.8	82.8	83.3	83.8	84.3	84.3	84.6	85.0	85.0	83.6	82.8	91.0	91.0	91.0	91.4	100.0	90.6	89.2	81.4	80.2	83.3	77.9	83.4
**17**	Apple (NP_001280931.1)	81.9	81.9	84.2	83.8	85.7	85.7	85.9	86.4	86.4	85.0	84.2	92.3	91.4	91.4	91.9	90.6	100.0	96.9	82.3	78.8	83.3	80.2	84.8
**18**	C_Pear (XP_009379167.1)	81.5	81.5	82.9	82.4	84.3	84.3	84.6	85.0	85.0	83.6	83.7	93.2	92.3	92.3	92.8	89.2	96.9	100.0	83.7	79.3	83.3	79.7	83.4
**19**	Pomegranate (OWM67504.1)	81.9	81.4	82.4	81.8	81.0	77.8	83.6	84.1	83.6	83.2	84.1	85.5	85.5	85.9	85.9	81.4	82.3	83.7	100.0	81.0	85.1	81.9	87.0
**20**	Pt_Poplar (RQO99258.1)	79.6	78.7	80.2	79.1	81.2	81.2	83.6	84.6	83.6	83.2	83.7	83.2	83.6	83.6	83.6	80.2	78.8	79.3	81.0	100.0	93.7	78.4	79.9
**21**	Pe_Poplar (XP_011031538.1)	84.1	83.2	86.8	86.4	84.7	84.7	88.1	88.1	87.2	86.8	86.8	87.7	88.1	88.1	88.1	83.3	83.3	83.3	85.1	93.7	100.0	81.0	84.3
**22**	Pistachio (XP_031270102.1)	83.8	82.9	82.4	81.9	83.4	83.4	83.3	83.3	82.8	82.4	81.9	81.8	82.7	82.3	82.3	77.9	80.2	79.7	81.9	78.4	81.0	100.0	81.6
**23**	Cucumber (NP_001267506.1)	84.2	82.9	86.9	87.8	84.8	84.8	86.4	87.3	87.8	87.3	86.9	87.8	86.9	87.3	87.3	83.4	84.8	83.4	87.0	79.9	84.3	81.6	100.0
**24**	Pumpkin (XP_023525464.1)	82.7	81.3	87.4	87.4	83.3	83.3	85.5	86.9	86.9	87.8	87.4	87.3	86.4	86.9	86.9	84.7	85.1	83.7	85.7	81.4	86.0	79.5	96.3
**25**	B_Melon (XP_022149992.1)	84.6	83.3	87.8	86.9	85.2	85.2	87.3	88.6	88.6	88.2	87.8	88.6	87.7	88.2	88.2	84.2	86.5	85.1	87.4	82.0	86.4	82.0	96.9
**26**	MGrape_BB (MW151566	80.6	80.6	82.8	81.9	81.2	81.2	84.2	84.2	83.3	83.3	83.3	82.3	82.3	82.3	82.7	80.6	82.0	81.1	84.7	81.1	85.5	81.2	82.1
**27**	BGrape_RL (MW1515649)	81.1	81.1	83.7	82.8	82.1	82.1	84.6	85.1	84.2	84.2	84.2	83.2	83.2	83.2	83.6	81.5	82.9	82.0	85.2	81.5	86.0	82.1	83.0
**28**	MGrape_JB (MW151564)	82.4	82.4	84.6	83.7	83.0	83.0	86.0	86.0	85.1	85.1	85.1	84.1	84.1	84.1	84.6	82.4	83.8	82.9	86.1	82.4	87.3	83.0	83.9
**29**	MGrape_F (MW151565)	82.4	82.4	84.6	83.7	83.0	83.0	86.0	86.0	85.1	85.1	85.1	84.1	84.1	84.1	84.6	82.4	83.8	82.9	86.1	82.4	87.3	83.0	83.9
**30**	MGrape_FS (MW151567)	82.4	82.4	84.6	83.7	83.0	83.0	86.0	86.0	85.1	85.1	85.1	84.1	84.1	84.1	84.6	82.4	83.8	82.9	86.1	82.4	87.3	83.0	83.9
**31**	BGrape_RS (MW151570)	81.5	81.5	84.2	83.3	82.5	82.5	85.1	85.5	84.6	84.6	84.6	83.6	83.6	83.6	84.1	82.0	83.3	82.4	85.7	82.0	86.4	82.5	83.4
**32**	HGrape_BDB (MW151568)	82.0	82.0	84.6	83.7	83.0	83.0	85.5	86.0	85.1	85.1	85.1	84.1	84.1	84.1	84.6	82.4	83.8	82.9	86.1	82.4	86.9	83.0	83.9
**33**	BGrape_CH (AKJ79177.1)	82.0	82.0	84.6	83.7	83.0	83.0	85.5	86.0	85.1	85.1	85.1	84.1	84.1	84.1	84.6	82.4	83.8	82.9	86.1	82.4	86.9	83.0	83.9
**34**	BGrape_ST (AKJ79179.1)	82.0	82.0	84.6	83.7	83.0	83.0	85.5	86.0	85.1	85.1	85.1	84.1	84.1	84.1	84.6	82.4	83.8	82.9	86.1	82.4	86.9	83.0	83.9
**35**	Wild_grape (XP_034677021.1)	82.0	82.0	82.8	81.5	83.0	83.0	85.5	86.0	85.1	85.1	85.1	84.1	84.1	84.1	84.6	82.4	83.8	82.9	86.1	80.3	86.9	83.0	83.9
**36**	Eucalyptus (BAH56659.1)	81.0	81.0	82.7	82.3	84.2	84.2	85.5	85.5	85.9	83.2	83.2	85.8	84.9	85.4	85.4	80.1	81.9	81.9	86.1	80.2	84.6	81.1	84.7
**37**	W_Myrtle (XP_030520885.1)	82.8	82.4	85.0	84.6	86.9	86.9	87.7	87.7	88.2	85.5	85.5	86.3	86.3	86.8	86.8	81.0	81.9	81.9	87.0	82.0	86.4	83.3	86.0
**38**	Gb_Cotton (KAB2031197.1)	81.5	81.1	83.3	83.3	84.4	84.4	86.4	85.5	86.0	86.0	85.6	82.4	82.4	82.8	82.8	78.5	80.3	80.3	84.7	80.7	84.7	82.1	84.8
**39**	Clementine (XP_024044079.1)	83.8	82.4	88.2	88.2	85.7	85.7	86.9	86.0	86.4	87.3	86.9	85.5	85.9	85.9	85.9	81.1	81.5	81.1	85.2	82.9	87.3	87.4	86.6
**40**	Orange (XP_024953929.1)	83.8	82.4	85.9	85.5	85.7	85.7	86.9	86.0	86.4	87.3	86.9	85.5	85.9	85.9	85.9	81.5	82.0	81.5	85.2	80.4	87.3	87.4	87.0
**41**	CastorBean (XP_015572387.1)	83.7	83.7	84.5	82.5	86.9	86.9	86.8	87.3	87.3	85.9	86.4	87.7	88.1	88.1	88.1	83.7	84.6	83.7	86.1	84.3	91.8	81.1	86.5
**42**	RubberTree (XP_021686554.1)	82.8	82.8	84.1	79.9	85.1	85.1	86.4	86.8	86.8	85.5	85.9	87.7	87.2	87.2	87.2	83.3	84.7	83.8	85.6	84.8	92.3	80.6	86.9
**43**	Cassava (XP_021614256.1)	83.7	83.7	87.3	85.9	86.0	86.0	87.3	87.7	87.7	86.4	86.8	87.7	88.1	88.1	88.1	84.2	84.6	83.7	86.5	88.2	92.7	81.5	86.9
**44**	Jatropha (XP_012073507.1)	83.3	83.3	86.8	85.5	85.1	85.1	86.8	86.4	86.4	85.9	86.4	86.8	87.2	87.2	87.2	84.2	84.6	83.7	85.6	86.9	92.3	81.5	86.0
**45**	Gh_Cotton (NP_001314025.1)	82.0	81.5	87.8	86.9	86.6	86.6	86.9	87.3	87.3	86.9	87.8	86.8	86.8	87.3	87.3	82.9	83.8	83.3	84.3	84.7	88.7	81.6	86.1
**46**	Durian (XP_022718367.1)	83.3	83.3	87.3	86.4	86.9	86.9	88.6	89.1	89.1	87.3	88.2	87.7	87.7	88.1	88.1	84.6	84.6	83.7	85.6	86.0	88.6	82.4	86.9
**47**	Cocoa (XP_007020164.1)	83.3	82.8	87.3	86.4	86.5	86.5	88.2	88.6	88.6	86.8	87.7	89.0	89.0	89.5	89.5	85.5	85.1	84.6	87.9	87.3	90.9	83.8	87.8
**48**	Radish (XP_018452529.1)	75.3	74.4	77.6	78.1	76.6	76.6	77.2	78.1	78.1	77.2	77.3	77.5	78.0	78.0	78.0	74.6	77.3	77.3	80.3	76.8	79.6	76.0	78.3

Table S3: Percent identity matrix of AGL11

	24	25	26	27	28	29	30	31	32	33	34	35	36	37	38	39	40	41	42	43	44	45	46	47
**1** W_Strawberry (XP_011461176.1)	82.7	84.6	80.6	81.1	82.4	82.4	82.4	81.5	82.0	82.0	82.0	82.0	81.0	82.8	81.5	83.8	83.8	83.7	82.8	83.7	83.3	82.0	83.3	83.3
**2** C_Rose (XP_024169047.1)	81.3	83.3	80.6	81.1	82.4	82.4	82.4	81.5	82.0	82.0	82.0	82.0	81.0	82.4	81.1	82.4	82.4	83.7	82.8	83.7	83.3	81.5	83.3	82.8
**3** W_Lupine (KAF1898823.1)	87.4	87.8	82.8	83.7	84.6	84.6	84.6	84.2	84.6	84.6	84.6	82.8	82.7	85.0	83.3	88.2	85.9	84.5	84.1	87.3	86.8	87.8	87.3	87.3
**4** B_Lupine (XP_019465480.1)	87.4	86.9	81.9	82.8	83.7	83.7	83.7	83.3	83.7	83.7	83.7	81.5	82.3	84.6	83.3	88.2	85.5	82.5	79.9	85.9	85.5	86.9	86.4	86.4
**5** WildPeanut (XP_016204599.1)	83.3	85.2	81.2	82.1	83.0	83.0	83.0	82.5	83.0	83.0	83.0	83.0	84.2	86.9	84.4	85.7	85.7	86.9	85.1	86.0	85.1	86.6	86.9	86.5
**6** Peanut (XP_025657296.1)	83.3	85.2	81.2	82.1	83.0	83.0	83.0	82.5	83.0	83.0	83.0	83.0	84.2	86.9	84.4	85.7	85.7	86.9	85.1	86.0	85.1	86.6	86.9	86.5
**7** Soybean (NP_001236130.1)	85.5	87.3	84.2	84.6	86.0	86.0	86.0	85.1	85.5	85.5	85.5	85.5	85.5	87.7	86.4	86.9	86.9	86.8	86.4	87.3	86.8	86.9	88.6	88.2
**8** PigeonPea (XP_020240314.1)	86.9	88.6	84.2	85.1	86.0	86.0	86.0	85.5	86.0	86.0	86.0	86.0	85.5	87.7	85.5	86.0	86.0	87.3	86.8	87.7	86.4	87.3	89.1	88.6
**9** CommonBean (XP_007148609.1)	86.9	88.6	83.3	84.2	85.1	85.1	85.1	84.6	85.1	85.1	85.1	85.1	85.9	88.2	86.0	86.4	86.4	87.3	86.8	87.7	86.4	87.3	89.1	88.6
**10** Chickpea (XP_004499566.1)	87.8	88.2	83.3	84.2	85.1	85.1	85.1	84.6	85.1	85.1	85.1	85.1	83.2	85.5	86.0	87.3	87.3	85.9	85.5	86.4	85.9	86.9	87.3	86.8
**11** Medicago (XP_003598035.1)	87.4	87.8	83.3	84.2	85.1	85.1	85.1	84.6	85.1	85.1	85.1	85.1	83.2	85.5	85.6	86.9	86.9	86.4	85.9	86.8	86.4	87.8	88.2	87.7
**12** Peach (XP_007223946.1)	87.3	88.6	82.3	83.2	84.1	84.1	84.1	83.6	84.1	84.1	84.1	84.1	85.8	86.3	82.4	85.5	85.5	87.7	87.7	87.7	86.8	86.8	87.7	89.0
**13** J_Apricot (XP_008219059.1)	86.4	87.7	82.3	83.2	84.1	84.1	84.1	83.6	84.1	84.1	84.1	84.1	84.9	86.3	82.4	85.9	85.9	88.1	87.2	88.1	87.2	86.8	87.7	89.0
**14** Almond (XP_034222093.1)	86.9	88.2	82.3	83.2	84.1	84.1	84.1	83.6	84.1	84.1	84.1	84.1	85.4	86.8	82.8	85.9	85.9	88.1	87.2	88.1	87.2	87.3	88.1	89.5
**15** S_Cherry (XP_021831176.1)	86.9	88.2	82.7	83.6	84.6	84.6	84.6	84.1	84.6	84.6	84.6	84.6	85.4	86.8	82.8	85.9	85.9	88.1	87.2	88.1	87.2	87.3	88.1	89.5
**16** A_Pear (AJW29026.1)	84.7	84.2	80.6	81.5	82.4	82.4	82.4	82.0	82.4	82.4	82.4	82.4	80.1	81.0	78.5	81.1	81.5	83.7	83.3	84.2	84.2	82.9	84.6	85.5
**17** Apple (NP_001280931.1)	85.1	86.5	82.0	82.9	83.8	83.8	83.8	83.3	83.8	83.8	83.8	83.8	81.9	81.9	80.3	81.5	82.0	84.6	84.7	84.6	84.6	83.8	84.6	85.1
**18** C_Pear (XP_009379167.1)	83.7	85.1	81.1	82.0	82.9	82.9	82.9	82.4	82.9	82.9	82.9	82.9	81.9	81.9	80.3	81.1	81.5	83.7	83.8	83.7	83.7	83.3	83.7	84.6
**19** Pomegranate (OWM67504.1)	85.7	87.4	84.7	85.2	86.1	86.1	86.1	85.7	86.1	86.1	86.1	86.1	86.1	87.0	84.7	85.2	85.2	86.1	85.6	86.5	85.6	84.3	85.6	87.9
**20** Pt_Poplar (RQO99258.1)	81.4	82.0	81.1	81.5	82.4	82.4	82.4	82.0	82.4	82.4	82.4	80.3	80.2	82.0	80.7	82.9	80.4	84.3	84.8	88.2	86.9	84.7	86.0	87.3
**21** Pe_Poplar (XP_011031538.1)	86.0	86.4	85.5	86.0	87.3	87.3	87.3	86.4	86.9	86.9	86.9	86.9	84.6	86.4	84.7	87.3	87.3	91.8	92.3	92.7	92.3	88.7	88.6	90.9
**22** Pistachio (XP_031270102.1)	79.5	82.0	81.2	82.1	83.0	83.0	83.0	82.5	83.0	83.0	83.0	83.0	81.1	83.3	82.1	87.4	87.4	81.1	80.6	81.5	81.5	81.6	82.4	83.8
**23** Cucumber (NP_001267506.1)	96.3	96.9	82.1	83.0	83.9	83.9	83.9	83.4	83.9	83.9	83.9	83.9	84.7	86.0	84.8	86.6	87.0	86.5	86.9	86.9	86.0	86.1	86.9	87.8
**24** Pumpkin (XP_023525464.1)	100.0	97.7	82.3	83.3	84.2	84.2	84.2	83.7	84.2	84.2	84.2	84.2	83.6	85.1	83.3	86.5	87.0	86.5	86.9	86.9	86.0	86.5	86.9	87.9
**25** B_Melon (XP_022149992.1)	97.7	100.0	83.3	84.2	85.1	85.1	85.1	84.7	85.1	85.1	85.1	85.1	85.5	86.9	84.8	86.0	86.5	88.2	88.7	88.7	87.8	86.5	87.8	88.7
**26** MGrape_BB (MW151566	82.3	83.3	100.0	96.4	97.8	97.8	97.8	97.8	97.3	97.3	97.3	97.3	81.5	83.3	80.7	84.3	84.3	86.0	85.6	86.5	86.9	83.9	84.7	85.6
**27** BGrape_RL (MW1515649)	83.3	84.2	96.4	100.0	98.7	98.7	98.7	98.7	99.1	99.1	99.1	99.1	82.4	84.2	81.6	85.2	85.2	86.9	86.5	87.4	87.4	84.8	85.6	86.5
**28** MGrape_JB (MW151564)	84.2	85.1	97.8	98.7	100.0	100.0	100.0	99.1	99.6	99.6	99.6	99.6	83.3	85.1	82.5	86.1	86.1	87.8	87.4	88.3	88.7	85.7	86.5	87.4
**29** MGrape_F (MW151565)	84.2	85.1	97.8	98.7	100.0	100.0	100.0	99.1	99.6	99.6	99.6	99.6	83.3	85.1	82.5	86.1	86.1	87.8	87.4	88.3	88.7	85.7	86.5	87.4
**30** MGrape_FS (MW151567)	84.2	85.1	97.8	98.7	100.0	100.0	100.0	99.1	99.6	99.6	99.6	99.6	83.3	85.1	82.5	86.1	86.1	87.8	87.4	88.3	88.7	85.7	86.5	87.4
**31** BGrape_RS (MW151570)	83.7	84.7	97.8	98.7	99.1	99.1	99.1	100.0	99.6	99.6	99.6	99.6	82.9	84.7	82.1	85.7	85.7	87.4	86.9	87.8	87.8	85.2	86.0	86.9
**32** HGrape_BDB (MW151568)	84.2	85.1	97.3	99.1	99.6	99.6	99.6	99.6	100.0	100.0	100.0	100.0	83.3	85.1	82.5	86.1	86.1	87.8	87.4	88.3	88.3	85.7	86.5	87.4
**33** BGrape_CH (AKJ79177.1)	84.2	85.1	97.3	99.1	99.6	99.6	99.6	99.6	100.0	100.0	100.0	100.0	83.3	85.1	82.5	86.1	86.1	87.8	87.4	88.3	88.3	85.7	86.5	87.4
**34** BGrape_ST (AKJ79179.1)	84.2	85.1	97.3	99.1	99.6	99.6	99.6	99.6	100.0	100.0	100.0	100.0	83.3	85.1	82.5	86.1	86.1	87.8	87.4	88.3	88.3	85.7	86.5	87.4
**35** Wild_grape (XP_034677021.1)	84.2	85.1	97.3	99.1	99.6	99.6	99.6	99.6	100.0	100.0	100.0	100.0	83.3	85.1	82.5	86.1	83.8	86.0	85.5	88.3	88.3	85.7	86.5	87.4
**36** Eucalyptus (BAH56659.1)	83.6	85.5	81.5	82.4	83.3	83.3	83.3	82.9	83.3	83.3	83.3	83.3	100.0	96.9	83.8	85.6	85.6	86.0	85.5	85.5	84.2	85.1	86.0	86.9
**37** W_Myrtle (XP_030520885.1)	85.1	86.9	83.3	84.2	85.1	85.1	85.1	84.7	85.1	85.1	85.1	85.1	96.9	100.0	85.6	87.8	87.8	88.2	86.4	87.3	86.0	87.4	88.2	88.7
**38** Gb_Cotton (KAB2031197.1)	83.3	84.8	80.7	81.6	82.5	82.5	82.5	82.1	82.5	82.5	82.5	82.5	83.8	85.6	100.0	88.3	88.3	86.9	86.9	87.8	86.9	89.2	89.6	90.1
**39** Clementine (XP_024044079.1)	86.5	86.0	84.3	85.2	86.1	86.1	86.1	85.7	86.1	86.1	86.1	86.1	85.6	87.8	88.3	100.0	99.6	87.8	87.4	88.3	87.4	90.6	89.2	90.1
**40** Orange (XP_024953929.1)	87.0	86.5	84.3	85.2	86.1	86.1	86.1	85.7	86.1	86.1	86.1	83.8	85.6	87.8	88.3	99.6	100.0	85.2	85.2	88.3	87.4	90.6	89.2	90.1
**41** CastorBean (XP_015572387.1)	86.5	88.2	86.0	86.9	87.8	87.8	87.8	87.4	87.8	87.8	87.8	86.0	86.0	88.2	86.9	87.8	85.2	100.0	95.6	98.6	97.3	91.9	93.2	94.1
**42** RubberTree (XP_021686554.1)	86.9	88.7	85.6	86.5	87.4	87.4	87.4	86.9	87.4	87.4	87.4	85.5	85.5	86.4	86.9	87.4	85.2	95.6	100.0	99.1	97.8	91.0	93.2	94.6
**43** Cassava (XP_021614256.1)	86.9	88.7	86.5	87.4	88.3	88.3	88.3	87.8	88.3	88.3	88.3	88.3	85.5	87.3	87.8	88.3	88.3	98.6	99.1	100.0	98.7	91.9	94.1	95.5
**44** Jatropha (XP_012073507.1)	86.0	87.8	86.9	87.4	88.7	88.7	88.7	87.8	88.3	88.3	88.3	88.3	84.2	86.0	86.9	87.4	87.4	97.3	97.8	98.7	100.0	91.0	92.8	94.1
**45** Gh_Cotton (NP_001314025.1)	86.5	86.5	83.9	84.8	85.7	85.7	85.7	85.2	85.7	85.7	85.7	85.7	85.1	87.4	89.2	90.6	90.6	91.9	91.0	91.9	91.0	100.0	94.6	95.5
**46** Durian (XP_022718367.1)	86.9	87.8	84.7	85.6	86.5	86.5	86.5	86.0	86.5	86.5	86.5	86.5	86.0	88.2	89.6	89.2	89.2	93.2	93.2	94.1	92.8	94.6	100.0	96.9
**47** Cocoa (XP_007020164.1)	87.9	88.7	85.6	86.5	87.4	87.4	87.4	86.9	87.4	87.4	87.4	87.4	86.9	88.7	90.1	90.1	90.1	94.1	94.6	95.5	94.1	95.5	96.9	100.0
**48** Radish (XP_018452529.1)	76.6	77.7	76.8	77.7	78.6	78.6	78.6	78.2	78.6	78.6	78.6	78.6	78.1	79.9	76.5	79.6	79.6	78.5	78.1	78.5	78.5	79.1	78.1	79.5

Table S3: Percent identity matrix of AGL11

		48	49	50	51	52	53	54	55	56	57	58	59	60	61	62	63	64	65	66	67	68	69
**1**	W_Strawberry (XP_011461176.1)	75.3	71.4	73.6	73.6	74.2	75.1	74.7	75.9	33.2	34.4	34.6	34.6	35.2	36.2	35.7	36.2	34.3	34.3	34.7	35.2	34.9	38.0
**2**	C_Rose (XP_024169047.1)	74.4	70.5	72.7	72.7	73.8	74.2	73.8	74.6	32.7	34.4	34.6	34.6	35.2	36.2	35.7	36.2	34.3	34.3	34.7	35.7	34.9	38.5
**3**	W_Lupine (KAF1898823.1)	77.6	71.5	74.6	73.3	75.3	76.2	75.8	80.0	33.2	34.4	34.6	34.6	35.8	36.3	35.8	36.3	34.9	34.9	35.1	35.8	35.1	36.7
**4**	B_Lupine (XP_019465480.1)	78.1	72.0	75.0	74.2	72.5	75.2	72.8	80.5	33.7	34.7	34.9	34.9	36.2	36.6	36.2	36.6	35.7	35.7	35.2	36.2	34.9	37.6
**5**	WildPeanut (XP_016204599.1)	76.6	71.3	74.0	74.0	77.7	78.6	78.1	81.5	32.0	34.1	34.3	34.3	36.0	36.0	35.5	36.0	35.1	34.6	35.1	36.0	34.7	38.3
**6**	Peanut (XP_025657296.1)	76.6	71.3	74.0	74.0	77.7	78.6	78.1	81.5	32.0	33.0	33.2	33.2	34.8	34.8	34.4	35.3	33.9	33.5	34.4	35.3	34.4	37.6
**7**	Soybean (NP_001236130.1)	77.2	72.3	75.0	75.0	77.8	78.7	78.3	81.8	31.4	34.6	34.8	34.8	34.9	35.9	35.4	36.8	34.9	34.4	34.9	35.9	34.1	37.7
**8**	PigeonPea (XP_020240314.1)	78.1	73.6	76.4	76.4	78.3	79.2	78.7	81.8	32.4	35.1	35.2	35.2	36.3	36.3	35.9	37.3	36.3	35.9	36.3	36.3	35.1	38.2
**9**	CommonBean (XP_007148609.1)	78.1	73.2	75.9	75.9	78.7	79.6	79.2	82.7	31.9	34.6	34.8	34.8	35.9	35.9	35.4	36.8	35.9	35.4	35.9	35.9	34.1	37.7
**10**	Chickpea (XP_004499566.1)	77.2	72.3	75.0	75.0	77.8	78.7	78.3	80.9	32.4	35.1	35.2	35.2	36.3	36.3	35.9	37.3	36.3	35.9	36.3	36.3	35.1	37.3
**11**	Medicago (XP_003598035.1)	77.3	72.4	75.1	75.1	77.5	78.4	77.9	80.5	32.4	34.9	35.1	35.1	36.3	36.3	35.9	37.3	36.3	35.9	36.3	36.3	35.1	37.3
**12**	Peach (XP_007223946.1)	77.5	72.6	75.3	75.3	76.8	77.7	77.3	78.5	34.8	36.3	36.5	36.5	37.3	38.2	37.7	37.3	36.3	35.9	36.3	36.8	36.0	40.1
**13**	J_Apricot (XP_008219059.1)	78.0	72.6	75.3	75.3	77.3	78.2	77.7	79.0	34.3	35.9	36.0	36.0	36.8	37.7	37.3	36.8	35.9	35.4	35.9	36.3	35.6	39.6
**14**	Almond (XP_034222093.1)	78.0	73.1	75.8	75.8	77.3	78.2	77.7	79.0	34.3	35.9	36.0	36.0	36.8	37.7	37.3	36.8	35.9	35.4	35.9	36.3	35.6	39.6
**15**	S_Cherry (XP_021831176.1)	78.0	73.1	75.8	75.8	77.3	78.2	77.7	79.0	34.3	35.9	36.0	36.0	36.8	37.7	37.3	36.8	35.9	35.4	35.9	36.3	35.6	39.6
**16**	A_Pear (AJW29026.1)	74.6	69.7	72.4	72.4	73.9	74.8	74.3	75.5	32.5	34.6	34.7	34.7	35.5	36.0	35.5	36.0	34.1	33.6	34.1	36.0	35.2	38.3
**17**	Apple (NP_001280931.1)	77.3	72.9	75.6	75.6	78.4	79.3	78.8	78.2	32.5	34.1	34.3	34.3	35.5	36.0	35.5	36.0	34.1	33.6	34.1	36.0	34.7	37.9
**18**	C_Pear (XP_009379167.1)	77.3	72.9	75.1	75.1	77.0	77.9	77.5	76.8	33.5	34.6	34.7	34.7	35.5	36.5	36.0	35.5	34.6	34.1	34.6	35.5	34.7	38.3
**19**	Pomegranate (OWM67504.1)	80.3	75.7	77.1	78.0	76.7	77.2	77.2	78.4	33.2	35.2	35.4	35.4	36.0	36.5	36.0	35.5	35.5	35.1	35.6	36.5	35.1	38.3
**20**	Pt_Poplar (RQO99258.1)	76.8	71.6	72.9	72.6	72.3	75.1	72.7	75.9	32.5	34.1	34.3	34.3	35.5	36.0	35.5	35.1	35.1	34.6	35.5	34.6	33.8	35.5
**21**	Pe_Poplar (XP_011031538.1)	79.6	74.2	75.6	76.5	77.5	78.9	78.4	78.6	34.2	35.7	35.9	35.9	36.2	37.6	37.1	36.6	35.7	35.2	36.2	36.2	35.4	36.6
**22**	Pistachio (XP_031270102.1)	76.0	72.1	73.0	73.9	73.5	74.4	73.5	76.9	33.0	34.7	34.9	34.9	36.0	36.5	36.0	36.5	35.1	34.6	35.5	36.5	35.2	39.3
**23**	Cucumber (NP_001267506.1)	78.3	74.3	76.6	76.6	78.0	78.1	78.0	80.1	32.4	34.9	35.1	35.1	36.7	36.7	36.3	36.7	35.8	35.8	36.3	36.7	35.5	39.1
**24**	Pumpkin (XP_023525464.1)	76.6	72.6	74.9	74.9	76.3	76.7	76.3	77.9	33.2	35.0	35.1	35.1	36.9	36.9	36.4	36.9	35.9	35.9	36.9	37.4	36.6	37.9
**25**	B_Melon (XP_022149992.1)	77.7	73.8	76.0	76.0	77.5	77.9	77.5	79.1	32.7	35.2	35.4	35.4	37.1	37.1	36.6	37.1	36.2	36.2	36.6	37.1	36.3	38.5
**26**	MGrape_BB (MW151566	76.8	72.9	74.2	75.1	74.8	76.1	75.7	75.5	32.0	33.8	34.0	34.0	34.6	35.5	35.1	35.5	33.6	33.2	34.1	36.0	36.6	36.5
**27**	BGrape_RL (MW1515649)	77.7	73.3	75.1	76.0	75.7	77.0	76.6	76.4	33.0	34.3	34.4	34.4	35.5	36.0	35.5	36.0	34.6	34.1	35.1	36.9	36.2	36.9
**28**	MGrape_JB (MW151564)	78.6	74.2	76.0	76.9	76.6	77.9	77.5	77.3	33.0	34.7	34.9	34.9	35.5	36.5	36.0	36.5	34.6	34.1	35.1	36.9	36.2	37.4
**29**	MGrape_F (MW151565)	78.6	74.2	76.0	76.9	76.6	77.9	77.5	77.3	33.0	34.7	34.9	34.9	35.5	36.5	36.0	36.5	34.6	34.1	35.1	36.9	36.2	37.4
**30**	MGrape_FS (MW151567)	78.6	74.2	76.0	76.9	76.6	77.9	77.5	77.3	33.0	34.7	34.9	34.9	35.5	36.5	36.0	36.5	34.6	34.1	35.1	36.9	36.2	37.4
**31**	BGrape_RS (MW151570)	78.2	74.2	75.6	76.5	76.1	77.5	77.0	76.8	32.5	33.8	34.0	34.0	35.1	35.5	35.1	35.5	34.1	33.6	34.6	36.5	36.6	36.9
**32**	HGrape_BDB (MW151568)	78.6	74.2	76.0	76.9	76.6	77.9	77.5	77.3	33.0	34.3	34.4	34.4	35.5	36.0	35.5	36.0	34.6	34.1	35.1	36.9	36.2	37.4
**33**	BGrape_CH (AKJ79177.1)	78.6	74.2	76.0	76.9	76.6	77.9	77.5	77.3	33.0	34.3	34.4	34.4	35.5	36.0	35.5	36.0	34.6	34.1	35.1	36.9	36.2	37.4
**34**	BGrape_ST (AKJ79179.1)	78.6	74.2	76.0	76.9	76.6	77.9	77.5	77.3	33.0	34.3	34.4	34.4	35.5	36.0	35.5	36.0	34.6	34.1	35.1	36.9	36.2	37.4
**35**	Wild_grape (XP_034677021.1)	78.6	73.9	76.0	75.2	74.6	75.9	75.4	77.3	33.0	34.3	34.4	34.4	35.5	36.0	35.5	36.0	34.6	34.1	35.1	36.9	36.2	37.4
**36**	Eucalyptus (BAH56659.1)	78.1	73.2	75.5	75.5	78.3	78.7	78.7	80.4	33.7	34.9	35.1	35.1	36.2	36.6	36.2	36.2	36.6	36.2	36.2	37.6	36.3	38.5
**37**	W_Myrtle (XP_030520885.1)	79.9	75.0	77.3	77.3	80.1	80.5	80.5	82.7	32.2	34.0	34.1	34.1	34.7	35.2	34.7	34.7	35.2	34.7	34.7	36.2	34.9	38.0
**38**	Gb_Cotton (KAB2031197.1)	76.5	73.0	73.4	74.3	75.8	77.1	76.7	78.7	31.6	34.1	34.3	34.3	35.5	35.5	35.1	35.5	34.6	34.1	34.1	35.1	34.3	37.4
**39**	Clementine (XP_024044079.1)	79.6	74.7	75.6	76.5	77.9	79.3	78.4	80.0	33.5	34.7	34.9	34.9	36.0	36.5	36.0	36.5	35.1	34.6	35.1	36.9	36.2	37.4
**40**	Orange (XP_024953929.1)	79.6	74.3	75.6	74.8	76.0	77.2	76.0	80.0	33.0	34.7	34.9	34.9	36.0	36.5	36.0	36.5	35.1	34.6	35.1	36.9	36.2	37.4
**41**	CastorBean (XP_015572387.1)	78.5	72.9	75.0	74.2	75.6	76.7	75.6	78.5	32.2	34.4	34.6	34.6	36.2	36.2	35.7	36.2	35.2	34.7	34.7	36.6	36.3	37.6
**42**	RubberTree (XP_021686554.1)	78.1	72.4	74.6	73.8	73.5	75.2	73.2	78.5	32.7	34.9	35.1	35.1	36.6	36.6	36.2	36.6	35.7	35.2	35.2	37.1	36.8	37.6
**43**	Cassava (XP_021614256.1)	78.5	73.2	75.0	75.9	77.8	78.7	78.3	79.0	32.2	34.4	34.6	34.6	36.2	36.2	35.7	36.2	35.2	34.7	34.7	36.2	35.9	37.6
**44**	Jatropha (XP_012073507.1)	78.5	72.7	74.6	75.5	77.8	78.7	78.3	79.0	32.2	34.9	35.1	35.1	35.7	36.6	36.2	36.6	34.7	34.3	34.3	36.2	35.9	37.6
**45**	Gh_Cotton (NP_001314025.1)	79.1	74.2	76.0	76.9	77.9	79.3	78.8	80.9	32.5	34.7	34.9	34.9	36.5	36.5	36.0	36.5	35.5	35.1	35.1	36.9	36.2	36.9
**46**	Durian (XP_022718367.1)	78.1	73.2	75.0	75.9	78.3	79.6	78.7	80.8	32.2	34.4	34.6	34.6	36.6	36.2	36.2	36.2	35.2	34.7	34.7	35.7	35.4	37.1
**47**	Cocoa (XP_007020164.1)	79.5	74.6	76.4	77.3	77.8	79.2	78.7	80.8	32.2	34.9	35.1	35.1	36.6	36.6	36.2	36.6	35.7	35.2	35.2	36.2	35.9	37.6
**48**	Radish (XP_018452529.1)	100.0	91.7	92.6	93.5	91.2	92.5	91.7	88.0	31.9	33.9	34.1	34.1	35.0	35.5	35.0	35.0	35.0	34.6	35.5	33.2	31.5	35.9

	1	2	3	4	5	6	7	8	9	10	11	12	13	14	15	16	17	18	19	20	21	22	23
**49** Cabbage (XP_013626799.1)	71.4	70.5	71.5	72.0	71.3	71.3	72.3	73.6	73.2	72.3	72.4	72.6	72.6	73.1	73.1	69.7	72.9	72.9	75.7	71.6	74.2	72.1	74.3
**50** Rapeseed (XP_022571869.1)	73.6	72.7	74.6	75.0	74.0	74.0	75.0	76.4	75.9	75.0	75.1	75.3	75.3	75.8	75.8	72.4	75.6	75.1	77.1	72.9	75.6	73.0	76.6
**51** Turnip (XP_033143784.1)	73.6	72.7	73.3	74.2	74.0	74.0	75.0	76.4	75.9	75.0	75.1	75.3	75.3	75.8	75.8	72.4	75.6	75.1	78.0	72.6	76.5	73.9	76.6
**52** C_rubella (XP_006286826.2)	74.2	73.8	75.3	72.5	77.7	77.7	77.8	78.3	78.7	77.8	77.5	76.8	77.3	77.3	77.3	73.9	78.4	77.0	76.7	72.3	77.5	73.5	78.0
**53** Camelina (XP_019084043.1)	75.1	74.2	76.2	75.2	78.6	78.6	78.7	79.2	79.6	78.7	78.4	77.7	78.2	78.2	78.2	74.8	79.3	77.9	77.2	75.1	78.9	74.4	78.1
**54** Arabidopsis (AT4G09960.3)	74.7	73.8	75.8	72.8	78.1	78.1	78.3	78.7	79.2	78.3	77.9	77.3	77.7	77.7	77.7	74.3	78.8	77.5	77.2	72.7	78.4	73.5	78.0
**55** T_Gourd (AXR70630.1)	75.9	74.6	80.0	80.5	81.5	81.5	81.8	81.8	82.7	80.9	80.5	78.5	79.0	79.0	79.0	75.5	78.2	76.8	78.4	75.9	78.6	76.9	80.1
**56** Rye (ADR51708.1)	33.2	32.7	33.2	33.7	32.0	32.0	31.4	32.4	31.9	32.4	32.4	34.8	34.3	34.3	34.3	32.5	32.5	33.5	33.2	32.5	34.2	33.0	32.4
**57** I_Rice (EAY87613.1)	34.4	34.4	34.4	34.7	34.1	33.0	34.6	35.1	34.6	35.1	34.9	36.3	35.9	35.9	35.9	34.6	34.1	34.6	35.2	34.1	35.7	34.7	34.9
**58** J_Rice (XP_015624915.1)	34.6	34.6	34.6	34.9	34.3	33.2	34.8	35.2	34.8	35.2	35.1	36.5	36.0	36.0	36.0	34.7	34.3	34.7	35.4	34.3	35.9	34.9	35.1
**59** Rice (ACY26068.1)	34.6	34.6	34.6	34.9	34.3	33.2	34.8	35.2	34.8	35.2	35.1	36.5	36.0	36.0	36.0	34.7	34.3	34.7	35.4	34.3	35.9	34.9	35.1
**60** Maize (ACG38901.1)	35.2	35.2	35.8	36.2	36.0	34.8	34.9	36.3	35.9	36.3	36.3	37.3	36.8	36.8	36.8	35.5	35.5	35.5	36.0	35.5	36.2	36.0	36.7
**61** F_Millet (XP_004953998.1)	36.2	36.2	36.3	36.6	36.0	34.8	35.9	36.3	35.9	36.3	36.3	38.2	37.7	37.7	37.7	36.0	36.0	36.5	36.5	36.0	37.6	36.5	36.7
**62** Sorghum (XP_021314301.1)	35.7	35.7	35.8	36.2	35.5	34.4	35.4	35.9	35.4	35.9	35.9	37.7	37.3	37.3	37.3	35.5	35.5	36.0	36.0	35.5	37.1	36.0	36.3
**63** P_Ryegrass (AAZ17549.1)	36.2	36.2	36.3	36.6	36.0	35.3	36.8	37.3	36.8	37.3	37.3	37.3	36.8	36.8	36.8	36.0	36.0	35.5	35.5	35.1	36.6	36.5	36.7
**64** B_Wheat (ABF57916.1)	34.3	34.3	34.9	35.7	35.1	33.9	34.9	36.3	35.9	36.3	36.3	36.3	35.9	35.9	35.9	34.1	34.1	34.6	35.5	35.1	35.7	35.1	35.8
**65** D_Wheat (VAI49577.1)	34.3	34.3	34.9	35.7	34.6	33.5	34.4	35.9	35.4	35.9	35.9	35.9	35.4	35.4	35.4	33.6	33.6	34.1	35.1	34.6	35.2	34.6	35.8
**66** Barley (KAE8787930.1)	34.7	34.7	35.1	35.2	35.1	34.4	34.9	36.3	35.9	36.3	36.3	36.3	35.9	35.9	35.9	34.1	34.1	34.6	35.6	35.5	36.2	35.5	36.3
**67** WM_Banana (XP_009391344.1)	35.2	35.7	35.8	36.2	36.0	35.3	35.9	36.3	35.9	36.3	36.3	36.8	36.3	36.3	36.3	36.0	36.0	35.5	36.5	34.6	36.2	36.5	36.7
**68** W_Banana (THU47278.1)	34.9	34.9	35.1	34.9	34.7	34.4	34.1	35.1	34.1	35.1	35.1	36.0	35.6	35.6	35.6	35.2	34.7	34.7	35.1	33.8	35.4	35.2	35.5
**69** AO_palm (XP_010942683.1)	38.0	38.5	36.7	37.6	38.3	37.6	37.7	38.2	37.7	37.3	37.3	40.1	39.6	39.6	39.6	38.3	37.9	38.3	38.3	35.5	36.6	39.3	39.1

	24	25	26	27	28	29	30	31	32	33	34	35	36	37	38	39	40	41	42	43	44	45	46	47
**49** Cabbage (XP_013626799.1)	72.6	73.8	72.9	73.3	74.2	74.2	74.2	74.2	74.2	74.2	74.2	73.9	73.2	75.0	73.0	74.7	74.3	72.9	72.4	73.2	72.7	74.2	73.2	74.6
**50** Rapeseed (XP_022571869.1)	74.9	76.0	74.2	75.1	76.0	76.0	76.0	75.6	76.0	76.0	76.0	76.0	75.5	77.3	73.4	75.6	75.6	75.0	74.6	75.0	74.6	76.0	75.0	76.4
**51** Turnip (XP_033143784.1)	74.9	76.0	75.1	76.0	76.9	76.9	76.9	76.5	76.9	76.9	76.9	75.2	75.5	77.3	74.3	76.5	74.8	74.2	73.8	75.9	75.5	76.9	75.9	77.3
**52** C_rubella (XP_006286826.2)	76.3	77.5	74.8	75.7	76.6	76.6	76.6	76.1	76.6	76.6	76.6	74.6	78.3	80.1	75.8	77.9	76.0	75.6	73.5	77.8	77.8	77.9	78.3	77.8
**53** Camelina (XP_019084043.1)	76.7	77.9	76.1	77.0	77.9	77.9	77.9	77.5	77.9	77.9	77.9	75.9	78.7	80.5	77.1	79.3	77.2	76.7	75.2	78.7	78.7	79.3	79.6	79.2
**54** Arabidopsis (AT4G09960.3)	76.3	77.5	75.7	76.6	77.5	77.5	77.5	77.0	77.5	77.5	77.5	75.4	78.7	80.5	76.7	78.4	76.0	75.6	73.2	78.3	78.3	78.8	78.7	78.7
**55** T_Gourd (AXR70630.1)	77.9	79.1	75.5	76.4	77.3	77.3	77.3	76.8	77.3	77.3	77.3	77.3	80.4	82.7	78.7	80.0	80.0	78.5	78.5	79.0	79.0	80.9	80.8	80.8
**56** Rye (ADR51708.1)	33.2	32.7	32.0	33.0	33.0	33.0	33.0	32.5	33.0	33.0	33.0	33.0	33.7	32.2	31.6	33.5	33.0	32.2	32.7	32.2	32.2	32.5	32.2	32.2
**57** I_Rice (EAY87613.1)	35.0	35.2	33.8	34.3	34.7	34.7	34.7	33.8	34.3	34.3	34.3	34.3	34.9	34.0	34.1	34.7	34.7	34.4	34.9	34.4	34.9	34.7	34.4	34.9
**58** J_Rice (XP_015624915.1)	35.1	35.4	34.0	34.4	34.9	34.9	34.9	34.0	34.4	34.4	34.4	34.4	35.1	34.1	34.3	34.9	34.9	34.6	35.1	34.6	35.1	34.9	34.6	35.1
**59** Rice (ACY26068.1)	35.1	35.4	34.0	34.4	34.9	34.9	34.9	34.0	34.4	34.4	34.4	34.4	35.1	34.1	34.3	34.9	34.9	34.6	35.1	34.6	35.1	34.9	34.6	35.1
**60** Maize (ACG38901.1)	36.9	37.1	34.6	35.5	35.5	35.5	35.5	35.1	35.5	35.5	35.5	35.5	36.2	34.7	35.5	36.0	36.0	36.2	36.6	36.2	35.7	36.5	36.6	36.6
**61** F_Millet (XP_004953998.1)	36.9	37.1	35.5	36.0	36.5	36.5	36.5	35.5	36.0	36.0	36.0	36.0	36.6	35.2	35.5	36.5	36.5	36.2	36.6	36.2	36.6	36.5	36.2	36.6
**62** Sorghum (XP_021314301.1)	36.4	36.6	35.1	35.5	36.0	36.0	36.0	35.1	35.5	35.5	35.5	35.5	36.2	34.7	35.1	36.0	36.0	35.7	36.2	35.7	36.2	36.0	36.2	36.2
**63** P_Ryegrass (AAZ17549.1)	36.9	37.1	35.5	36.0	36.5	36.5	36.5	35.5	36.0	36.0	36.0	36.0	36.2	34.7	35.5	36.5	36.5	36.2	36.6	36.2	36.6	36.5	36.2	36.6
**64** B_Wheat (ABF57916.1)	35.9	36.2	33.6	34.6	34.6	34.6	34.6	34.1	34.6	34.6	34.6	34.6	36.6	35.2	34.6	35.1	35.1	35.2	35.7	35.2	34.7	35.5	35.2	35.7
**65** D_Wheat (VAI49577.1)	35.9	36.2	33.2	34.1	34.1	34.1	34.1	33.6	34.1	34.1	34.1	34.1	36.2	34.7	34.1	34.6	34.6	34.7	35.2	34.7	34.3	35.1	34.7	35.2
**66** Barley (KAE8787930.1)	36.9	36.6	34.1	35.1	35.1	35.1	35.1	34.6	35.1	35.1	35.1	35.1	36.2	34.7	34.1	35.1	35.1	34.7	35.2	34.7	34.3	35.1	34.7	35.2
**67** WM_Banana (XP_009391344.1)	37.4	37.1	36.0	36.9	36.9	36.9	36.9	36.5	36.9	36.9	36.9	36.9	37.6	36.2	35.1	36.9	36.9	36.6	37.1	36.2	36.2	36.9	35.7	36.2
**68** W_Banana (THU47278.1)	36.6	36.3	36.6	36.2	36.2	36.2	36.2	36.6	36.2	36.2	36.2	36.2	36.3	34.9	34.3	36.2	36.2	36.3	36.8	35.9	35.9	36.2	35.4	35.9
**69** AO_palm (XP_010942683.1)	37.9	38.5	36.5	36.9	37.4	37.4	37.4	36.9	37.4	37.4	37.4	37.4	38.5	38.0	37.4	37.4	37.4	37.6	37.6	37.6	37.6	36.9	37.1	37.6

Figure S1

```
VvAGL11_CH_gDNA       TCGCCGGATTTTGGAAACAAGGTGTGTAGGTGATCTTTTAACAGAAACGTTTCACCACCA         60
VvAGL11_ST_gDNA       TCGCCGGATTTTGGAAACAAGGTGTGTAGGTGATCTTTTAACAGAAACGTTTCACCACCA         60
VroAGL11_JB_gDNA      TCGCCGGATTTTGGAA-CAAGGTGTGTAGGTGATCTTTTAACAGAAACNTTTCACCACCA         59
VroAGL11_JB_mRNA      ------------------------------------------------------------         0
VvAGL11_CH_mRNA       ------------------------------------------------------------         0
VvAGL11_ST_mRNA       ------------------------------------------------------------         0

VvAGL11_CH_gDNA       AAATTTCCACAATCAACCATTCTCTCTCTGTGAAAACGTTTCGTGCATAACTGGGTAATC         120
VvAGL11_ST_gDNA       AAATTTCCACAATCAACCATTCTCTCTCTGTGAAAACGTTTCGTGCATAACTGGGTAATC         120
VroAGL11_JB_gDNA      AAATTTCCACAATCAACCATTCTCTCTCTGTGAAAACGTTTCGTGGATAACTGGGTAATC         119
VroAGL11_JB_mRNA      ------------------------------------------------------------         0
VvAGL11_CH_mRNA       ------------------------------------------------------------         0
VvAGL11_ST_mRNA       ------------------------------------------------------------         0

VvAGL11_CH_gDNA       TTAGATCTGCTCCCCTCCACACCACAGAATCTACTTTTGCCTACATATGAACATCTGCTT         180
VvAGL11_ST_gDNA       TTAGATCTGCTCCCCTCCACACCACAGAATCTACTTTTGCCTACATATGAACATCTGCTT         180
VroAGL11_JB_gDNA      TTACATCTGCTCCGCTCCACAGCACAGAATCGACTTTTGCCTACATATGAACATCTCCTT         179
VroAGL11_JB_mRNA      ------------------------------------------------------------         0
VvAGL11_CH_mRNA       ------------------------------------------------------------         0
VvAGL11_ST_mRNA       ------------------------------------------------------------         0

VvAGL11_CH_gDNA       TCCATTTCTTCCTCTTTCTTTTGTCAGTGCCCATCTCTCCAAATTTACTTCCACCTCTTA         240
VvAGL11_ST_gDNA       TCCATTTCTTCCTCTTTCTTTTGTCAGTGCCCATCTCTCCAAATTTACTTCCACCTCTTA         240
VroAGL11_JB_gDNA      TCCATTTCTTCCCCTTTCTTTTGTCAGTGCCCATCTCTCCAAGTTTACTTCCACCTCTTA         239
VroAGL11_JB_mRNA      ------------------------------------------------------------         0
VvAGL11_CH_mRNA       ------------------------------------------------------------         0
VvAGL11_ST_mRNA       ------------------------------------------------------------         0

VvAGL11_CH_gDNA       CATTTTCTTACCATTCTTTTTAGATTTCTTGGCTTGATTTACTCTCTTCTTCCTGCAACA         300
VvAGL11_ST_gDNA       CATTTTCTTACCATTCTTTTTAGATTTCTTGGCTTGATTTACTCTCTTCTTCCTGCAACA         300
VroAGL11_JB_gDNA      CATTTTCTTACCATTCTTTTTAGATTTCTTGGCTTGATTTACTCTCGTCTACCTGCAACA         299
VroAGL11_JB_mRNA      ------------------------------------------------------------         0
VvAGL11_CH_mRNA       ------------------------------------------------------------         0
VvAGL11_ST_mRNA       ------------------------------------------------------------         0

VvAGL11_CH_gDNA       CTTTACTCTTCAGTTCTTGATTCTCTTTTGCCTTCTTCATGCATTTGTTTCCATTCTTTA         360
VvAGL11_ST_gDNA       CTTTACTCTTCAGTTCTTGATTCTCTTTTGCCTTCTTCATGCATTTGTTTCCATTCTTTA         360
VroAGL11_JB_gDNA      CTTTACTCTTCACTTCTTGATTCTCTTTTGCCTTCTTCATGCATTTGTTTACATTCTTTA         359
VroAGL11_JB_mRNA      ------------------------------------------------------------         0
VvAGL11_CH_mRNA       ------------------------------------------------------------         0
VvAGL11_ST_mRNA       ------------------------------------------------------------         0

VvAGL11_CH_gDNA       ATTAGTCATTTTCTTATTCTAAACTTTCTTTTCCCTTTTTTCGTTATTTTCAGAATGCAG         420
VvAGL11_ST_gDNA       ATTAGTCATTTTCTTATTCTAAACTTTCTTTTCCCTTTTTTCGTTATTTTCAGAATGCAG         420
VroAGL11_JB_gDNA      ATTAGTCATTTCTTTTNCTAAGCTTTCTTTTCCCTTTTTTCGTTATTTTCAGAAGGCAG         419
VroAGL11_JB_mRNA      ------------------------------------------------------------         0
VvAGL11_CH_mRNA       ------------------------------------------------------------         0
VvAGL11_ST_mRNA       ------------------------------------------------------------         0

VvAGL11_CH_gDNA       TTTGATTATTTTTCCTTTTTTTCATGTTAAAACAGTATTTGTTTTGGTAGTTTTCAGTTT         480
VvAGL11_ST_gDNA       TTTGATTATTTTTCCTTTTTTTCATGTTAAAACAGTATTTGTTTTGGTAGTTTTCAGTTT         480
VroAGL11_JB_gDNA      TGAGATTATTTTGCCTTGGGTTCATCTTAAAACAGTATTTGTTTTGGTAGTTTTCAGTTT         479
VroAGL11_JB_mRNA      ------------------------------------------------------------         0
VvAGL11_CH_mRNA       ------------------------------------------------------------         0
VvAGL11_ST_mRNA       ------------------------------------------------------------         0

VvAGL11_CH_gDNA       ATTTTTTCCATAAATTTCTGTTTCAAAAACCTCTTGAGGGGAAAAAGCCAGAATTTTTTT         540
VvAGL11_ST_gDNA       ATTTTTTCCATAAATTTCTGTTTCAAAAACCTCTTGAGGGGAAAAAGCCAGAATTTTTTT         540
VroAGL11_JB_gDNA      ATTTTTTCCATAAATTTCTGTTTCAAAAACCTCTTGAGGGGAAAAAGCCAGAATTTTTTT         539
VroAGL11_JB_mRNA      ------------------------------------------------------------         0
VvAGL11_CH_mRNA       ------------------------------------------------------------         0
VvAGL11_ST_mRNA       ------------------------------------------------------------         0

VvAGL11_CH_gDNA       TCCTGCGTTCTGGTCTTAAGCTTCTCATCCATATCCTGGGTTTATGGGAAATGTGTTAAG         600
VvAGL11_ST_gDNA       TCCTGCGTTCTGGTCTTAAGCTTCTCATCCATATCCTGGGTTTATGGGAAATGTGTTAAG         600
VroAGL11_JB_gDNA      TCCTGCGTTCTGGTCTTAAGCTTCTCATCCATATCCTGGGTTTATGGGAAATGTGTTAAG         599
VroAGL11_JB_mRNA      ------------------------------------------------------------         0
VvAGL11_CH_mRNA       ------------------------------------------------------------         0
VvAGL11_ST_mRNA       ------------------------------------------------------------         0

VvAGL11_CH_gDNA       GGTTATGGGGTTTTTGAGAAATGTGGAGAGAGAGAGAGAGAGATGTGTAGACATGCACCC         660
VvAGL11_ST_gDNA       GGTTATGGGGTTTTTGAGAAATGTGGAGAGAGAGAGAGAGAGATGTGTAGACATGCACCC         660
VroAGL11_JB_gDNA      GGTTATGGGGTTTTTGAGAAATGTGGAGAGAGAGAGAGAGAGATGTGTAGACATGCACCC         659
VroAGL11_JB_mRNA      ------------------------------------------------------------         0
VvAGL11_CH_mRNA       ------------------------------------------------------------         0
VvAGL11_ST_mRNA       ------------------------------------------------------------         0
```

```
VvAGL11_CH_gDNA       ACTTGTAGTAGTATTAGTAGTGGAGATAGATCCTGGGTTGATTTTTCAAATGGTAGATCA       720
VvAGL11_ST_gDNA       ACTTGTAGTAGTATTAGTAGTGGAGATAGATCCTGGGTTGATTTTTCAAATGGTAGATCA       720
VroAGL11_JB_gDNA      ACTTGTAGTAGTATTAGTAGTGGAGATAGATCCTGGGTTGATTTTTCAAATGGTAGATCA       719
VroAGL11_JB_mRNA      ------------------------------------------------------------       0
VvAGL11_CH_mRNA       ------------------------------------------------------------       0
VvAGL11_ST_mRNA       ------------------------------------------------------------       0

VvAGL11_CH_gDNA       TGTTCTCTTCTCTTTGTTCTCATCTCTTCATGTTCTTTTTTCCTCAGTGGCTTACAGATT       780
VvAGL11_ST_gDNA       TGTTCTCTTCTCTTTGTTCTCATCTCTTCATGTTCTTTTTTCCTCAGTGGCTTACAGATT       780
VroAGL11_JB_gDNA      TGTTCTCTTCTCTTTGTTCTCATCTCTTCATGTTCTTTTTTCCTCAGTGGCTTACAGATT       779
VroAGL11_JB_mRNA      ------------------------------------------------------------       0
VvAGL11_CH_mRNA       ------------------------------------------------------------       0
VvAGL11_ST_mRNA       ------------------------------------------------------------       0

VvAGL11_CH_gDNA       TTCTGTCTCCCAATCTTTTGGTTTCCATCTCTTTTCCAAGATCTCCTTATTTTCCTACAT       840
VvAGL11_ST_gDNA       TTCTGTCTCCCAATCTTTTGGTTTCCATCTCTTTTCCAAGATCTCCTTATTTTCCTACAT       840
VroAGL11_JB_gDNA      TTCTGTCTCCCAATCTTTTGGTTTCCATCTCTTTTCCAAGATCTCCTTATTTTCCTACAT       839
VroAGL11_JB_mRNA      ------------------------------------------------------------       0
VvAGL11_CH_mRNA       ------------------------------------------------------------       0
VvAGL11_ST_mRNA       ------------------------------------------------------------       0

VvAGL11_CH_gDNA       TATCATCATTATTTTATTATATACACAAGCCTCACCCTATTTTTCGAACAACATCTAACT       900
VvAGL11_ST_gDNA       TATCATCATTATTTTATTATATACACAAGCCTCACCCTATTTTTCGAACAACATCTAACT       900
VroAGL11_JB_gDNA      TATCATCATTATTTTATTATATACACAAGCCTCACCCTATTTTTCGAACAACATCTAACT       899
VroAGL11_JB_mRNA      ------------------------------------------------------------       0
VvAGL11_CH_mRNA       ------------------------------------------------------------       0
VvAGL11_ST_mRNA       ------------------------------------------------------------       0

VvAGL11_CH_gDNA       TTCTCATTTCTCAGTCTATCATCATTCAGATCTCTCCTCTCTCCTCTCTCCTCTTCACCC       960
VvAGL11_ST_gDNA       TTCTCATTTCTCAGTCTATCATCATTCAGATCTCTCCTCTCTCCTCTCTCCTCTTCACCC       960
VroAGL11_JB_gDNA      TTCTCATTTCTCAGTCTATCATCATTCAGATCTCTCCTCTCTCCTCTCTCCTCTTCACCC       959
VroAGL11_JB_mRNA      ------------------------------------------------------------       0
VvAGL11_CH_mRNA       ------------------------------------------------------------       0
VvAGL11_ST_mRNA       ------------------------------------------------------------       0

VvAGL11_CH_gDNA       TCTACCAAAAACACACATTTTTTTCGGTAGATCTTCTCTCACAAATTACATGCATTATTAC       1020
VvAGL11_ST_gDNA       TCTACCAAAAACACACATTTTTTTCGGTAGATCTTCTCTCACAAATTACATGCATTATTAC       1020
VroAGL11_JB_gDNA      TCTACCAAAAACACACATTTTTTTCGGTAGATCTTCTCTCACAAATTACATGCATTATTAC       1019
VroAGL11_JB_mRNA      ------------------------------------------------------------       0
VvAGL11_CH_mRNA       ------------------------------------------------------------       0
VvAGL11_ST_mRNA       ------------------------------------------------------------       0

VvAGL11_CH_gDNA       CCTTCATATGTCACTCATACTTCAATTTTTTTTTTTTTTTTAAAGCTTGAACAGATCTCC       1080
VvAGL11_ST_gDNA       CCTTCATATGTCACTCATACTTCAATTTTTTTTTTTTTTTTAAAGCTTGAACAGATCTCC       1080
VroAGL11_JB_gDNA      CCTTCATATGTCACTCATACTTCAATTTTTTTTTTTTTTTTAAAGCTTGAACAGATCTCC       1079
VroAGL11_JB_mRNA      ------------------------------------------------------------       0
VvAGL11_CH_mRNA       ------------------------------------------------------------       0
VvAGL11_ST_mRNA       ------------------------------------------------------------       0

VvAGL11_CH_gDNA       AACTCTTTCTCTAGTATTTTGTAATCTTTAGCAGTTTCATAAAGAGATGCTTTGGTAAAT       1140
VvAGL11_ST_gDNA       AACTCTTTCTCTAGTATTTTGTAATCTTTAGCAGTTTCATAAAGAGATGCTTTGGTAAAT       1140
VroAGL11_JB_gDNA      AACTCTTTCTCTAGTATTTTGTAATCTTTAGCAGTTTCATAAAGAGATGCTTTGGTAAAT       1139
VroAGL11_JB_mRNA      ------------------------------------------------------------       0
VvAGL11_CH_mRNA       ------------------------------------------------------------       0
VvAGL11_ST_mRNA       ------------------------------------------------------------       0

VvAGL11_CH_gDNA       TTTTCTAATATTTTCAGTTGGTGATATAATATAACAATGTATAGTACTACAAAAATTTTG       1200
VvAGL11_ST_gDNA       TTTTCTAATATTTTCAGTTGGTGATATAATATAACAATGTATAGTACTACAAAAATTTTG       1200
VroAGL11_JB_gDNA      TTTTCTAATATTTTCAGTTGGTGATATAATATAACAATGTATAGTACTACAAAAATTTTG       1199
VroAGL11_JB_mRNA      ------------------------------------------------------------       0
VvAGL11_CH_mRNA       ------------------------------------------------------------       0
VvAGL11_ST_mRNA       ------------------------------------------------------------       0

VvAGL11_CH_gDNA       TTCTTATATACCTAAGCCATTTTTTTCTTTTGGTGCAATAAACCTAACAGTGTAGTGAACA       1260
VvAGL11_ST_gDNA       TTCTTATATACCTAAGCCATTTTTTTCTTTTGGTGCAATAAACCTAACAGTGTAGTGAACA       1260
VroAGL11_JB_gDNA      TTCTTATATACCTAAGCCATTTTTTTCTTTTGGTGCAATAAACCTAACAGTGTAGTGAACA       1259
VroAGL11_JB_mRNA      -----------------------------------------------------------A       1
VvAGL11_CH_mRNA       -----------------------------------------------------------A       1
VvAGL11_ST_mRNA       -----------------------------------------------------------A       1
                                                                                 *

VvAGL11_CH_gDNA       TGGGGAGAGGAAAGATCGAGATCAAGAGGATCGAAAACACGACCAACCGTCAGGTCACAT       1320
VvAGL11_ST_gDNA       TGGGGAGAGGAAAGATCGAGATCAAGAGGATCGAAAACACGACCAACCGTCAGGTCACAT       1320
VroAGL11_JB_gDNA      TGGGGAGAGGAAAGATCGAGATCAAGAGGATCGAAAACACGACGAACCGTCAGGTCACAT       1319
VroAGL11_JB_mRNA      TGGGGAGAGGAAAGATCGAGATCAAGAGGATCGAAAACACGACGAACCGTCAGGTCACAT       61
VvAGL11_CH_mRNA       TGGGGAGAGGAAAGATCGAGATCAAGAGGATCGAAAACACGACCAACCGTCAGGTCACAT       61
VvAGL11_ST_mRNA       TGGGGAGAGGAAAGATCGAGATCAAGAGGATCGAAAACACGACCAACCGTCAGGTCACAT       61
                      ******************************************* ****************
```

```
VvAGL11_CH_gDNA      TCTGCAAGCGAAGGAATGGGCTTTTGAAGAAGGCTTATGAATTATCAGTGCTATGTGATG     1380
VvAGL11_ST_gDNA      TCTGCAAGCGAAGGAATGGGCTTTTGAAGAAGGCTTATGAATTATCAGTGCTATGTGATG     1380
VroAGL11_JB_gDNA     TCTGCAAGCGAAGGAATGGGCTTTTGAAGAAGGCTTATGAATTATCAGTGCTATGTGATG     1379
VroAGL11_JB_mRNA     TCTGCAAGCGAAGGAATGGGCTTTTGAAGAAGGCTTATGAATTATCAGTGCTATGTGATG     121
VvAGL11_CH_mRNA      TCTGCAAGCGAAGGAATGGGCTTTTGAAGAAGGCTTATGAATTATCAGTGCTATGTGATG     121
VvAGL11_ST_mRNA      TCTGCAAGCGAAGGAATGGGCTTTTGAAGAAGGCTTATGAATTATCAGTGCTATGTGATG     121
                     ************************************************************

VvAGL11_CH_gDNA      CAGAAGTTGCCCTCATCGTCTTCTCCAGCCGCGGTCGAGTCTATGAGTACTCAAACAACA     1440
VvAGL11_ST_gDNA      CAGAAGTTGCCCTCATCGTCTTCTCCAGCCGCGGTCGAGTCTATGAGTACTCAAACAACA     1440
VroAGL11_JB_gDNA     CAGAAGTTGCCCTCATTGTCTTCTCCAGCCGCGGTCGCGTCTATGAGTACTCAAACAACA     1439
VroAGL11_JB_mRNA     CAGAAGTTGCCCTCATTGTCTTCTCCAGCCGCGGTCGCGTCTATGAGTACTCAAACAACA     181
VvAGL11_CH_mRNA      CAGAAGTTGCCCTCATCGTCTTCTCCAGCCGCGGTCGAGTCTATGAGTACTCAAACAACA     181
VvAGL11_ST_mRNA      CAGAAGTTGCCCTCATCGTCTTCTCCAGCCGCGGTCGAGTCTATGAGTACTCAAACAACA     181
                     **************** ******************** **********************

VvAGL11_CH_gDNA      AGTAATAATTTTCTCCACCATTTCTTCAACCATCTGCTGAATTTTCTATGTTCATCTTTC     1500
VvAGL11_ST_gDNA      AGTAATAATTTTCTCCACCATTTCTTCAACCATCTGCTGAATTTTCTATGTTCATCTTTC     1500
VroAGL11_JB_gDNA     AGTAATAATTTTCTCCACCATTTCTTCAACCATCTGCTGAATTTTCTATGTTCATCTTTC     1499
VroAGL11_JB_mRNA     A-----------------------------------------------------------     182
VvAGL11_CH_mRNA      A-----------------------------------------------------------     182
VvAGL11_ST_mRNA      A-----------------------------------------------------------     182
                     *

VvAGL11_CH_gDNA      TCCATTTTGGTGCATCATACGGGCAATTAATGGTTTTATATTGATGAGATTAATATCATCA    1561
VvAGL11_ST_gDNA      TCCATTTTGGTGCATCATACGGGCAATTAATGGTTTTATATTGATGAGATTAATATCATCA    1561
VroAGL11_JB_gDNA     TCCATTTTGGTGCATCATACGGGCAATTAATGGTTTTATATTGATGAGATTAATATCATCA    1560
VroAGL11_JB_mRNA     ------------------------------------------------------------     182
VvAGL11_CH_mRNA      ------------------------------------------------------------     182
VvAGL11_ST_mRNA      ------------------------------------------------------------     182

VvAGL11_CH_gDNA      GAAGTTGCAGAACCCTAATGTTATTAAAGCAAAGAAAAAAAAAAAGAACAGTGGAAGGAG     1621
VvAGL11_ST_gDNA      GAAGTTGCAGAACCCTAATGTTATTAAAGCAAAGAAAAAAAAAAAGAACAGTGGAAGGAG     1621
VroAGL11_JB_gDNA     GAAGTTGCAGAACCCTAATGTTATTAAAGCAAAGAAAAAAAAAAAGAACAGTGGAAGGAG     1620
VroAGL11_JB_mRNA     ------------------------------------------------------------     182
VvAGL11_CH_mRNA      ------------------------------------------------------------     182
VvAGL11_ST_mRNA      ------------------------------------------------------------     182

VvAGL11_CH_gDNA      GAGATGCAATAAATTTGAAAATCTAACTCACTGCAGATTATGATTTTTTATGAGGGGAGT     1681
VvAGL11_ST_gDNA      GAGATGCAATAAATTTGAAAATCTAACTCACTGCAGATTATGATTTTTTATGAGGGGAGT     1681
VroAGL11_JB_gDNA     GAGATGCAATAAATTTGAAAATCTAACTCACTGCAGATTATGATTTTTTATGAGGGGAGT     1680
VroAGL11_JB_mRNA     ------------------------------------------------------------     182
VvAGL11_CH_mRNA      ------------------------------------------------------------     182
VvAGL11_ST_mRNA      ------------------------------------------------------------     182

VvAGL11_CH_gDNA      CAGATTCCTTTTGCTTTATTTAGGAAGGGATTACATGGGTACCTGCTTTGATATTTATGA     1741
VvAGL11_ST_gDNA      CAGATTCCTTTTGCTTTATTTAGGAAGGGATTACATGGGTACCTGCTTTGATATTTATGA     1741
VroAGL11_JB_gDNA     CAGATTCCTTTTGCTTTATTTAGGAAGGGATTACATGGGTACCTGCTTTGATATTTATGA     1740
VroAGL11_JB_mRNA     ------------------------------------------------------------     182
VvAGL11_CH_mRNA      ------------------------------------------------------------     182
VvAGL11_ST_mRNA      ------------------------------------------------------------     182

VvAGL11_CH_gDNA      TAATTTTCTTTTATCAAAATTTAAATGAAGGAAAATGCAGATAATATTCTGACATTTCAT     1801
VvAGL11_ST_gDNA      TAATTTTCTTTTATCAAAATTTAAATGAAGGAAAATGCAGATAATATTCTGACATTTCAT     1801
VroAGL11_JB_gDNA     TAATTTTCTTTTATCAAAATTTAAATGAAGGAAA-TGCAGATAATATTCTGACATTTCAT     1800
VroAGL11_JB_mRNA     ------------------------------------------------------------     182
VvAGL11_CH_mRNA      ------------------------------------------------------------     182
VvAGL11_ST_mRNA      ------------------------------------------------------------     182

VvAGL11_CH_gDNA      CTGGCATCTGCTGATGAATCTGAGAAAACAATTTCTTCTTCATGATTTTTCACTAATTAC     1861
VvAGL11_ST_gDNA      CTGGCATCTGCTGATGAATCTGAGAAAACAATTTCTTCTTCATGATTTTTCACTAATTAC     1861
VroAGL11_JB_gDNA     CTGGCATCTGCTGATGAAATCTGAGAAAAAATGTCTTCTTCATGATTTTTCACTAATTAC     1860
VroAGL11_JB_mRNA     ------------------------------------------------------------     182
VvAGL11_CH_mRNA      ------------------------------------------------------------     182
VvAGL11_ST_mRNA      ------------------------------------------------------------     182

VvAGL11_CH_gDNA      ATCTTTGACTGATATAAGGAAAATATCAGAAAGAACACTATACCTATAGTTAGAAACTTC     1921
VvAGL11_ST_gDNA      ATCTTTGACTGATATAAGGAAAATATCAGAAAGAACACTATACCTATAGTTAGAAACTTC     1921
VroAGL11_JB_gDNA     ATCTTTGACTGATATAAGGAAAATATCAGAAAGAACACTATACCTATAGTTAGAAACTTC     1920
VroAGL11_JB_mRNA     ------------------------------------------------------------     182
VvAGL11_CH_mRNA      ------------------------------------------------------------     182
VvAGL11_ST_mRNA      ------------------------------------------------------------     182

VvAGL11_CH_gDNA      TTCAAAGATTAACTATCAGAGGGTTCTTTTTATTGTATGTTTAGTGCATTAAAAAAAAATA     1981
VvAGL11_ST_gDNA      TTCAAAGATTAACTATCAGAGGGTTCTTTTTATTGTATGTTTAGTGCATTAAAAAAAAATA     1981
VroAGL11_JB_gDNA     TTCAAAGATTAACTATCAGAGGGTTCTTTTTATTGTATGTTTAGTGCATTAAAAAAAAATA     1980
VroAGL11_JB_mRNA     ------------------------------------------------------------     182
VvAGL11_CH_mRNA      ------------------------------------------------------------     182
VvAGL11_ST_mRNA      ------------------------------------------------------------     182
```

```
VvAGL11_CH_gDNA      AAAATTATAATGAAAAAATAAAAATAAAATAGGAAAAAAAATCGAAAAAGTTGAAATCCA      2041
VvAGL11_ST_gDNA      AAAATTATAATGAAAAAATAAAAATAAAATAGGAAAAAAAATCGAAAAAGTTGAAATCCA      2041
VroAGL11_JB_gDNA     AAAATTATAATGAAAAAATAAAAATAAAATAGGAAAAAAAATCGAAAAAGTTGAAATCCA      2040
VroAGL11_JB_mRNA     ------------------------------------------------------------      182
VvAGL11_CH_mRNA      ------------------------------------------------------------      182
VvAGL11_ST_mRNA      ------------------------------------------------------------      182

VvAGL11_CH_gDNA      ACTATCAGTTACACTGGTAGTGCTGATATGCATGCAAGGGAAGACAATCTAGGCAACAAC      2101
VvAGL11_ST_gDNA      ACTATCAGTTACACTGGTAGTGCTGATATGCATGCAAGGGAAGACAATCTAGGCAACAAC      2101
VroAGL11_JB_gDNA     ACTATCAGTTACACTGGTAGTGCTGATATGCATGCAAGGGAAGACAATCTAGGCAACAAC      2100
VroAGL11_JB_mRNA     ------------------------------------------------------------      182
VvAGL11_CH_mRNA      ------------------------------------------------------------      182
VvAGL11_ST_mRNA      ------------------------------------------------------------      182

VvAGL11_CH_gDNA      CAGTTAGGGTTTCTTGTCCTAGCTAGTTCTTCCCATCTCTCCTTACTGTTCCTTTCCAAT      2161
VvAGL11_ST_gDNA      CAGTTAGGGTTTCTTGTCCTAGCTAGTTCTTCCCATCTCTCCTTACTGTTCCTTTCCAAT      2161
VroAGL11_JB_gDNA     CAGTTAGGGTTTCTTGTCCTAGCTAGTTCTTCCCATCTCTCCTTACTGTTCCTTTCCAAT      2160
VroAGL11_JB_mRNA     ------------------------------------------------------------      182
VvAGL11_CH_mRNA      ------------------------------------------------------------      182
VvAGL11_ST_mRNA      ------------------------------------------------------------      182

VvAGL11_CH_gDNA      AATATTTAACATTGTAATCTGTGAGAATCTGTATTCCACTGTCCACAGTACAGTATTTAA      2221
VvAGL11_ST_gDNA      AATATTTAACATTGTAATCTGTGAGAATCTGTATTCCACTGTCCACAGTACAGTATTTAA      2221
VroAGL11_JB_gDNA     AATATTTAACATTGTAATCTGTGAGAATCTGTATTCCACTGTCCACAGTACAGTATTTAA      2220
VroAGL11_JB_mRNA     ------------------------------------------------------------      182
VvAGL11_CH_mRNA      ------------------------------------------------------------      182
VvAGL11_ST_mRNA      ------------------------------------------------------------      182

VvAGL11_CH_gDNA      GAAAAGGGTTATGGCCCTTTTCCTCTTCCACTCTTTGTTCAAGTCTCAGTTTCTCTGCTC      2281
VvAGL11_ST_gDNA      GAAAAGGGTTATGGCCCTTTTCCTCTTCCACTCTTTGTTCAAGTCTCAGTTTCTCTGCTC      2281
VroAGL11_JB_gDNA     GAAAAGGGTTATGGCCCTTTTCCTCTTCCACTCTTTGTTCAAGTCTCAGTTTCTCTGCTC      2280
VroAGL11_JB_mRNA     ------------------------------------------------------------      182
VvAGL11_CH_mRNA      ------------------------------------------------------------      182
VvAGL11_ST_mRNA      ------------------------------------------------------------      182

VvAGL11_CH_gDNA      TTCTCTTTCCATTTCCAGCTTTGGGAGGCTTGGAGCCCATGTATCATAAATCCTTCCCTT      2341
VvAGL11_ST_gDNA      TTCTCTTTCCATTTCCAGCTTTGGGAGGCTTGGAGCCCATGTATCATAAATCCTTCCCTT      2341
VroAGL11_JB_gDNA     TTCTCTTTCCATTTCCAGCTTTGGGAGGCTTGGAGCCCATGTATCATAAATCCTTCCCTT      2340
VroAGL11_JB_mRNA     ------------------------------------------------------------      182
VvAGL11_CH_mRNA      ------------------------------------------------------------      182
VvAGL11_ST_mRNA      ------------------------------------------------------------      182

VvAGL11_CH_gDNA      GTTTTTCTCCATCTTTTTGTTTTGGGTTTCTTCAGCCTAAAAGCTGTGAACCTTTCAATG      2401
VvAGL11_ST_gDNA      GTTTTTCTCCATCTTTTTGTTTTGGGTTTCTTCAGCCTAAAAGCTGTGAACCTTTCAATG      2401
VroAGL11_JB_gDNA     GTTTTTCTCCATCTTTTTGTTTTGGGTTTCTTCAGCCTAAAAGCTGTGAACCTTTCAATG      2400
VroAGL11_JB_mRNA     ------------------------------------------------------------      182
VvAGL11_CH_mRNA      ------------------------------------------------------------      182
VvAGL11_ST_mRNA      ------------------------------------------------------------      182

VvAGL11_CH_gDNA      GAGTCTTTTGGTCCTCTCTTCTCTTTCCCATTCTCATTTAAAGTGCAGTAAAGTGATCAG      2461
VvAGL11_ST_gDNA      GAGTCTTTTGGTCCTCTCTTCTCTTTCCCATTCTCATTTAAAGTGCAGTAAAGTGATCAG      2461
VroAGL11_JB_gDNA     GAGTCTTTTGGTCCTCTCTTCTCTTTCCCATTCTCATTTAAAGTGCAGTAAAGTGATCAG      2460
VroAGL11_JB_mRNA     ------------------------------------------------------------      182
VvAGL11_CH_mRNA      ------------------------------------------------------------      182
VvAGL11_ST_mRNA      ------------------------------------------------------------      182

VvAGL11_CH_gDNA      CTTTCACTGCTCTTTGTTTTTTTCACTTGGTGTGGGCAATTTGTGGATATCAAATCTCAG      2521
VvAGL11_ST_gDNA      CTTTCACTGCTCTTTGTTTTTTTCACTTGGTGTGGGCAATTTGTGGATATCAAATCTCAG      2521
VroAGL11_JB_gDNA     CTTTCACTGCTCTTTGTTTTTTTCACTTGGTGTGGGCAATTTGTGGATATCAAATCTCAG      2520
VroAGL11_JB_mRNA     ------------------------------------------------------------      182
VvAGL11_CH_mRNA      ------------------------------------------------------------      182
VvAGL11_ST_mRNA      ------------------------------------------------------------      182

VvAGL11_CH_gDNA      AAATGGGACGGTGTACATCAGCACCCATTACATCTTTGTGTGGGTTTGAATAAATATTTC      2581
VvAGL11_ST_gDNA      AAATGGGACGGTGTACATCAGCACCCATTACATCTTTGTGTGGGTTTGAATAAATATTTC      2581
VroAGL11_JB_gDNA     AAATGGGACGGTGTACATCAGCACCCATTACATCTTTGTGTGGGTTTGAATAAATATTTC      2580
VroAGL11_JB_mRNA     ------------------------------------------------------------      182
VvAGL11_CH_mRNA      ------------------------------------------------------------      182
VvAGL11_ST_mRNA      ------------------------------------------------------------      182

VvAGL11_CH_gDNA      ATATCAATCATTTCATGTTTGCTCAATGCCATCCAAGATTTTTTTTCTTCACTTCATGTC      2641
VvAGL11_ST_gDNA      ATATCAATCATTTCATGTTTGCTCAATGCCATCCAAGATTTTTTTTCTTCACTTCATGTC      2641
VroAGL11_JB_gDNA     ATATCAATCATTTCATGTTTGCTCAATGCCATCCAAGATTTTTTTTCTTCACTTCATGTC      2640
VroAGL11_JB_mRNA     ------------------------------------------------------------      182
VvAGL11_CH_mRNA      ------------------------------------------------------------      182
VvAGL11_ST_mRNA      ------------------------------------------------------------      182
```

```
VvAGL11_CH_gDNA      TCATTTTCCTCCTTGATTTCTCCCTTGTTCAAGGAAAATTGAAAATCTTCTCATAGTTGA      2701
VvAGL11_ST_gDNA      TCATTTTCCTCCTTGATTTCTCCCTTGTTCAAGGAAAATTGAAAATCTTCTCATAGTTGA      2701
VroAGL11_JB_gDNA     TCATTTTCCTCCTTGATTTCTCCCTTGTTCAAGGAAAATTGAAAATCTTCTCATAGTTGA      2700
VroAGL11_JB_mRNA     ------------------------------------------------------------      182
VvAGL11_CH_mRNA      ------------------------------------------------------------      182
VvAGL11_ST_mRNA      ------------------------------------------------------------      182

VvAGL11_CH_gDNA      GATAGTATTTAAAATAGGACATTGATCTTACATGAGATTTTACCTTTTTTTTTTCAAAAT      2761
VvAGL11_ST_gDNA      GATAGTATTTAAAATAGGACATTGATCTTACATGAGATTTTACCTTTTTTTTTTCAAAAT      2761
VroAGL11_JB_gDNA     GATAGTATTTAAAATAGGACATTGATCTTACATGAGATTTTACCTTTTTTTTTTCAAAAT      2760
VroAGL11_JB_mRNA     ------------------------------------------------------------      182
VvAGL11_CH_mRNA      ------------------------------------------------------------      182
VvAGL11_ST_mRNA      ------------------------------------------------------------      182

VvAGL11_CH_gDNA      TTTAAATTATTTTTTATACTTTTTAAAAATACTTGTTTTTACCCTTAATCAATTAACAAA      2821
VvAGL11_ST_gDNA      TTTAAATTATTTTTTATACTTTTTAAAAATACTTGTTTTTACCCTTAATCAATTAACAAA      2821
VroAGL11_JB_gDNA     TTTAAATTATTTTTTATACTTTTTAAAAATACTTGTTTTTACCCTTAATCAATTAACAAA      2820
VroAGL11_JB_mRNA     ------------------------------------------------------------      182
VvAGL11_CH_mRNA      ------------------------------------------------------------      182
VvAGL11_ST_mRNA      ------------------------------------------------------------      182

VvAGL11_CH_gDNA      AAAAATGAAAAATGAAAAAAAGACCAATTTGAATTTTTTTTTTAATGTTGAGGGTGCATG      2881
VvAGL11_ST_gDNA      AAAAATGAAAAATGAAAAAAAGACCAATTTGAATTTTTTTTTTAATGTTGAGGGTGCATG      2881
VroAGL11_JB_gDNA     AAAAATGAAAAATGAAAAAAAGACCAATTTGAATTTTTTTTTTAATGTTGAGGGTGCATG      2880
VroAGL11_JB_mRNA     ------------------------------------------------------------      182
VvAGL11_CH_mRNA      ------------------------------------------------------------      182
VvAGL11_ST_mRNA      ------------------------------------------------------------      182

VvAGL11_CH_gDNA      GGGGTGGGGGTGGAGGGGAGAAGTCTTCCTTATTATTTATTAATTTTAATGCATTATTTT      2941
VvAGL11_ST_gDNA      GGGGTGGGGGTGGAGGGGAGAAGTCTTCCTTATTATTTATTAATTTTAATGCATTATTTT      2941
VroAGL11_JB_gDNA     GGGGTGGGGGTGGAGGGGAGAAGTCTTCCTTATTATTTATTAATTTTAATGCATTATTTT      2940
VroAGL11_JB_mRNA     ------------------------------------------------------------      182
VvAGL11_CH_mRNA      ------------------------------------------------------------      182
VvAGL11_ST_mRNA      ------------------------------------------------------------      182

VvAGL11_CH_gDNA      TAGTCAAATCCAATTATATTGCATAAAATTAATTAACATCATCTTTGATCATCTTTAAAT      3001
VvAGL11_ST_gDNA      TAGTCAAATCCAATTATATTGCATAAAATTAATTAACATCATCTTTGATCATCTTTAAAT      3001
VroAGL11_JB_gDNA     TAGTCAAATCCAATTATATTGCATAAAATTAATTAACATCATCTTTGATCATCTTTAAAT      3000
VroAGL11_JB_mRNA     ------------------------------------------------------------      182
VvAGL11_CH_mRNA      ------------------------------------------------------------      182
VvAGL11_ST_mRNA      ------------------------------------------------------------      182

VvAGL11_CH_gDNA      CATATGGATTAAATCGTATTGTTGCTTATTTTCCTTATTCAAATAGAATAAAAATTATTT      3061
VvAGL11_ST_gDNA      CATATGGATTAAATCGTATTGTTGCTTATTTTCCTTATTCAAATAGAATAAAAATTATTT      3061
VroAGL11_JB_gDNA     CATATGGATTAAATCGTATTGTTGCTTATTTTCCTTATTCAAATAGAATAAAAATTATTT      3060
VroAGL11_JB_mRNA     ------------------------------------------------------------      182
VvAGL11_CH_mRNA      ------------------------------------------------------------      182
VvAGL11_ST_mRNA      ------------------------------------------------------------      182

VvAGL11_CH_gDNA      TAAGATTCTTTATTCTTTATATATGAGAGAAAAATAAAAGAAATAAATTTGATAAAATAT      3121
VvAGL11_ST_gDNA      TAAGATTCTTTATTCTTTATATATGAGAGAAAAATAAAAGAAATAAATTTGATAAAATAT      3121
VroAGL11_JB_gDNA     TAAGATTCTTTATTCTTTATATATGAGAGAAAAATAAAAGAAATAAATTTGATAAAATAT      3120
VroAGL11_JB_mRNA     ------------------------------------------------------------      182
VvAGL11_CH_mRNA      ------------------------------------------------------------      182
VvAGL11_ST_mRNA      ------------------------------------------------------------      182

VvAGL11_CH_gDNA      GTGAAAAAATTATTAAGTTCAAGCTTTTTTTATTTATTTTTTATTTTTTCATCATTTTTT      3181
VvAGL11_ST_gDNA      GTGAAAAAATTATTAAGTTCAAGCTTTTTTTATTTATTTTTTATTTTTTCATCATTTTTT      3181
VroAGL11_JB_gDNA     GTGAAAAAATTATTAAGTTCAAGCTTTTTTTATTTATTTTTTATTTTTTCATCATTTTTT      3180
VroAGL11_JB_mRNA     ------------------------------------------------------------      182
VvAGL11_CH_mRNA      ------------------------------------------------------------      182
VvAGL11_ST_mRNA      ------------------------------------------------------------      182

VvAGL11_CH_gDNA      TTTGTTTCTTTTTTTTCTCTCTCTTAAATTTTTGAAGATTTAAATGCAACCCCTACACTT      3241
VvAGL11_ST_gDNA      TTTGTTTCTTTTTTTTCTCTCTCTTAAATTTTTGAAGATTTAAATGCAACCCCTACACTT      3241
VroAGL11_JB_gDNA     TTTGTTTCTTTTTTTTCTCTCTCTTAAATTTTTGAAGATTTAAATGCAACCCCTACACTT      3240
VroAGL11_JB_mRNA     ------------------------------------------------------------      182
VvAGL11_CH_mRNA      ------------------------------------------------------------      182
VvAGL11_ST_mRNA      ------------------------------------------------------------      182

VvAGL11_CH_gDNA      TACACCTAAGAGAGTTTGAGGAAAAGTAGGAGAAAAAGAAAACAAAGAAGAAAAATGAAA      3301
VvAGL11_ST_gDNA      TACACCTAAGAGAGTTTGAGGAAAAGTAGGAGAAAAAGAAAACAAAGAAGAAAAATGAAA      3301
VroAGL11_JB_gDNA     TACACCTAAGAGAGTTTGAGGAAAAGTAGGAGAAAAAGAAAACAAAGAAGAAAAATGAAA      3300
VroAGL11_JB_mRNA     ------------------------------------------------------------      182
VvAGL11_CH_mRNA      ------------------------------------------------------------      182
VvAGL11_ST_mRNA      ------------------------------------------------------------      182
```

```
VvAGL11_CH_gDNA      GAAAGAAAAAGAAAAATAAAGAAATATGTTTAAATTCAATAAATTGTTATTACTTATTTT      3361
VvAGL11_ST_gDNA      GAAAGAAAAAGAAAAATAAAGAAATATGTTTAAATTCAATAAATTGTTATTACTTATTTT      3261
VroAGL11_JB_gDNA     GAAAGAAAAAGAAAAATAAAGAAATATGTTTAAATTCAATAAATTGTTATTACTTATTTT      3260
VroAGL11_JB_mRNA     ------------------------------------------------------------      182
VvAGL11_CH_mRNA      ------------------------------------------------------------      182
VvAGL11_ST_mRNA      ------------------------------------------------------------      182

VvAGL11_CH_gDNA      TCTAAAATTATTTTTATTCTTTTCTCTCATATACAACCAAACAAAATTTCAAAAATGTAA      3421
VvAGL11_ST_gDNA      TCTAAAATTATTTTTATTCTTTTCTCTCATATACAACCAAACAAAATTTCAAAAATGTAA      3421
VroAGL11_JB_gDNA     TCTAAAATTATTTTTATTCTTTTCTCTCATATACAACCAAACAAAATTTCAAAAATGTAA      3420
VroAGL11_JB_mRNA     ------------------------------------------------------------      182
VvAGL11_CH_mRNA      ------------------------------------------------------------      182
VvAGL11_ST_mRNA      ------------------------------------------------------------      182

VvAGL11_CH_gDNA      AATTTTTGATATAGTTTTCATTAGATTTGATTTTTCATACTTTCCATAATAATCCAAACA      3481
VvAGL11_ST_gDNA      AATTTTTGATATAGTTTTCATTAGATTTGATTTTTCATACTTTCCATAATAATCCAAACA      3481
VroAGL11_JB_gDNA     AATTTTTGATATAGTTTTCATTAGATTTGATTTTTCATACTTTCCATAATAATCCAAACA      3480
VroAGL11_JB_mRNA     ------------------------------------------------------------      182
VvAGL11_CH_mRNA      ------------------------------------------------------------      182
VvAGL11_ST_mRNA      ------------------------------------------------------------      182

VvAGL11_CH_gDNA      AGATCAATTAATAAACCGAAACAAAACCTACAATGACTTCATAATAACGGAAGTTTAAAG      3541
VvAGL11_ST_gDNA      AGATCAATTAATAAACCGAAACAAAACCTACAATGACTTCATAATAACGGAAGTTTAAAG      3541
VroAGL11_JB_gDNA     AGATCAATTAATAAACCGAAACAAAACCTACAATGACTTCATAATAACGGAAGTTTAAAG      3540
VroAGL11_JB_mRNA     ------------------------------------------------------------      182
VvAGL11_CH_mRNA      ------------------------------------------------------------      182
VvAGL11_ST_mRNA      ------------------------------------------------------------      182

VvAGL11_CH_gDNA      GAACTAAGGATGAATTGAGATAGATTCGAGGATGGTGTCATAATGATAGGAGTCCAAATT      3601
VvAGL11_ST_gDNA      GAACTAAGGATGAATTGAGATAGATTCGAGGATGGTGTCATAATGATAGGAGTCCAAATT      3601
VroAGL11_JB_gDNA     GAACTAAGGATGAATTGAGATAGATTCGAGGATGGTGTCATAATGATAGGAGTCCAAATT      3600
VroAGL11_JB_mRNA     ------------------------------------------------------------      182
VvAGL11_CH_mRNA      ------------------------------------------------------------      182
VvAGL11_ST_mRNA      ------------------------------------------------------------      182

VvAGL11_CH_gDNA      TGGAGGTAAGAATAAACTTAAATAAACTCTAAGACGATGCTCCAATGATGAATTTCTTTT      3661
VvAGL11_ST_gDNA      TGGAGGTAAGAATAAACTTAAATAAACTCTAAGACGATGCTCCAATGATGAATTTCTTTT      3661
VroAGL11_JB_gDNA     TGGAGGTAAGAATAAACTTAAATAAACTCTAAGACGATGCTCCAATGATGAATTTCTTTT      3660
VroAGL11_JB_mRNA     ------------------------------------------------------------      182
VvAGL11_CH_mRNA      ------------------------------------------------------------      182
VvAGL11_ST_mRNA      ------------------------------------------------------------      182

VvAGL11_CH_gDNA      TTAAAATATTTGAGTAGGCATTTGTCATTAATTTTTCTAACTACAAATAGATTACCAATA      3721
VvAGL11_ST_gDNA      TTAAAATATTTGAGTAGGCATTTGTCATTAATTTTTCTAACTACAAATAGATTACCAATA      3721
VroAGL11_JB_gDNA     TTAAAATATTTGAGTAGGCATTTGTCATTAATTTTTCTAACTACAAATAGATTACCAATA      3720
VroAGL11_JB_mRNA     ------------------------------------------------------------      182
VvAGL11_CH_mRNA      ------------------------------------------------------------      182
VvAGL11_ST_mRNA      ------------------------------------------------------------      182

VvAGL11_CH_gDNA      AAATGATCATAATCCCTTTCAACCGAACTTGTCGGTTGTTGTATTGTTTTTTATGATATT      3781
VvAGL11_ST_gDNA      AAATGATCATAATCCCTTTCAACCGAACTTGTCGGTTGTTGTATTGTTTTTTATGATATT      3781
VroAGL11_JB_gDNA     AAATGATCATAATCCCTTTCAACCGAACTTGTCGGTTGTTGTATTGTTTTTTATGATATT      3780
VroAGL11_JB_mRNA     ------------------------------------------------------------      182
VvAGL11_CH_mRNA      ------------------------------------------------------------      182
VvAGL11_ST_mRNA      ------------------------------------------------------------      182

VvAGL11_CH_gDNA      TTTATGTAACATGTTAAATTATAAAATAAATAAATAAGTTATAAGAAGAAAGAAAAAACC      3841
VvAGL11_ST_gDNA      TTTATGTAACATGTTAAATTATAAAATAAATAAATAAGTTATAAGAAGAAAGAAAAAACC      3841
VroAGL11_JB_gDNA     TTTATGTAACATGTTAAATTATAAAATAAATAAATAAGTTATAAGAAGAAAGAAAAAACC      3840
VroAGL11_JB_mRNA     ------------------------------------------------------------      182
VvAGL11_CH_mRNA      ------------------------------------------------------------      182
VvAGL11_ST_mRNA      ------------------------------------------------------------      182

VvAGL11_CH_gDNA      AATTCAATTCCAATAAAAACCTTTGAAATTAATAAATGGTGGGATATGTGATAGAGGTGC      3901
VvAGL11_ST_gDNA      AATTCAATTCCAATAAAAACCTTTGAAATTAATAAATGGTGGGATATGTGATAGAGGTGC      3901
VroAGL11_JB_gDNA     AATTCAATTCCAATAAAAACCTTTGAAATTAATAAATGGTGGGATATGTGATAGAGGTGC      3900
VroAGL11_JB_mRNA     ------------------------------------------------------------      182
VvAGL11_CH_mRNA      ------------------------------------------------------------      182
VvAGL11_ST_mRNA      ------------------------------------------------------------      182

VvAGL11_CH_gDNA      AAAGTCTTGGGTTCAATTACCATCATTAAGAATCCCTGGATTATTCGGAGTTGATTCTAA      3961
VvAGL11_ST_gDNA      AAAGTCTTGGGTTCAATTACCATCATTAAGAATCCCTGGATTATTCGGAGTTGATTCTAA      3961
VroAGL11_JB_gDNA     AAAGTCTTGGGTTCAATTACCATCATTAAGAATCCCTGGATTATTCGGAGTTGATTCTAA      3960
VroAGL11_JB_mRNA     ------------------------------------------------------------      182
VvAGL11_CH_mRNA      ------------------------------------------------------------      182
VvAGL11_ST_mRNA      ------------------------------------------------------------      182
```

```
VvAGL11_CH_gDNA      TGGGTGTCGTTGGAATCCCTAAGGTGTCGAAGCCATGGGTGGATTTGAAAGGCCCTATCA      4021
VvAGL11_ST_gDNA      TGGGTGTCGTTGGAATCCCTAAGGTGTCGAAGCCATGGGTGGATTTGAAAGGCCCTATCA      4021
VroAGL11_JB_gDNA     TGGGTGTCGTTGGAATCCCTAAGGTGTCGAAGCCATGGGTGGATTTGAAAGGCCCTATCA      4020
VroAGL11_JB_mRNA     ------------------------------------------------------------      182
VvAGL11_CH_mRNA      ------------------------------------------------------------      182
VvAGL11_ST_mRNA      ------------------------------------------------------------      182

VvAGL11_CH_gDNA      CAGTGGGGTTTCCGGTTTATAAAAAACACACACACACACACACAATTTTCATAATAGAAG      4081
VvAGL11_ST_gDNA      CAGTGGGGTTTCCGGTTTATAAAAAACACAC----ACACACACAATTTTCATAATAGAAG      4077
VroAGL11_JB_gDNA     CAGTGGGGTTTCCGGTTTATAAAAAACACACACACACACACACAATTTTCATAATAGAAG      4080
VroAGL11_JB_mRNA     ------------------------------------------------------------      182
VvAGL11_CH_mRNA      ------------------------------------------------------------      182
VvAGL11_ST_mRNA      ------------------------------------------------------------      182

VvAGL11_CH_gDNA      GTAGAAAAAAGTGAGAATAAGGTAGAGAAGATGTTTTTTCCTAGCTATAGGTACTCTAGG      4141
VvAGL11_ST_gDNA      GTAGAAAAAAGTGAGAATAAGGTAGAGAAGATGTTTTTTCCTAGCTATAGGTACTCTAGG      4137
VroAGL11_JB_gDNA     GTAGAAAAAAGTGAGAATAAGGTAGAGAAGATGTTTTTTCCTAGCTATAGGTACTCTAGG      4140
VroAGL11_JB_mRNA     ------------------------------------------------------------      182
VvAGL11_CH_mRNA      ------------------------------------------------------------      182
VvAGL11_ST_mRNA      ------------------------------------------------------------      182

VvAGL11_CH_gDNA      GTTTTGTGATAATAGATTTGGATTTTTTTCCCCAATAATTAAAATTATTAATTCAATTAT      4201
VvAGL11_ST_gDNA      GTTTTGTGATAATAGATTTGGATTTTTTTCCCCAATAATTAAAATTATTAATTCAATTAT      4197
VroAGL11_JB_gDNA     GTTTTGTGATAATAGATTTGGATTTTTTTCCCCAATAATTAAAATTATTAATTCAATTAT      4200
VroAGL11_JB_mRNA     ------------------------------------------------------------      182
VvAGL11_CH_mRNA      ------------------------------------------------------------      182
VvAGL11_ST_mRNA      ------------------------------------------------------------      182

VroAGL11_JB_gDNA     AATAAATATAAAAATCAATGAGAAATTCCATGGAAACCCAAATCAAATGTTGCAATATAG      4261
VvAGL11_ST_gDNA      AATAAATATAAAAATCAATGAGAAATTCCATGGAAACCCAAATCAAATGTTGCAATATAG      4257
VvAGL11_CH_gDNA      AATAAATATAAAAATCAATGAGAAATTCCATGGAAACCCAAATCAAATGTTGCAATATAG      4260
VroAGL11_JB_mRNA     ------------------------------------------------------------      182
VvAGL11_CH_mRNA      ------------------------------------------------------------      182
VvAGL11_ST_mRNA      ------------------------------------------------------------      182

VvAGL11_CH_gDNA      ATCAAGATTAGCATTTACTTATATGCATGCATGTTAATTAGTTTGACCTTAAAGATCTTG      4321
VvAGL11_ST_gDNA      TTCAAGATTAGCATTTACTTATATGCATGCATGTTAATTAGTTTGACCTTAAAGATCTTG      4317
VroAGL11_JB_gDNA     ATCAAGATTAGCATTTACTTATATGCATGCATGTTAATTATTTTGACCTCAAAGATCTTG      4320
VroAGL11_JB_mRNA     ------------------------------------------------------------      182
VvAGL11_CH_mRNA      ------------------------------------------------------------      182
VvAGL11_ST_mRNA      ------------------------------------------------------------      182

VvAGL11_CH_gDNA      TAGTACTGGTTCCCAATATAAAAATGAGAGCCTATTTGGG--ATAACTTTTTAACACCTG      4379
VvAGL11_ST_gDNA      TAGTACTGGTTCCCAATATAAAAATGAGAGCCTATTTGGG--ATAACTTTTTAACACCTG      4375
VroAGL11_JB_gDNA     TAGTACTGGTTCCCNGTATAAAAATAAGAGCCTATTTGGGAGATAACCTTTTAACTCCTG      4380
VroAGL11_JB_mRNA     ------------------------------------------------------------      182
VvAGL11_CH_mRNA      ------------------------------------------------------------      182
VvAGL11_ST_mRNA      ------------------------------------------------------------      182

VvAGL11_CH_gDNA      CATCTAGTAATTAGAGATGAAACATACATTATTTGTATTATGTAATCTATTTTATGAACA      4439
VvAGL11_ST_gDNA      CATCTAGTAATTAGAGATGAAACATACATTATTTGTATTATGTAATCTATTTTATGAACA      4435
VroAGL11_JB_gDNA     CATCTGGTAATTAGAGATGAAACAGACATTATTTGTATTATGTAATTCATTTTATGAACA      4440
VroAGL11_JB_mRNA     ------------------------------------------------------------      182
VvAGL11_CH_mRNA      ------------------------------------------------------------      182
VvAGL11_ST_mRNA      ------------------------------------------------------------      182

VvAGL11_CH_gDNA      AAATTTTAAAAATGCTTTAATAAATAGTGAGGATGACTATGATATTTCAAAAAAATTTTA      4499
VvAGL11_ST_gDNA      AAATTTTAAAAATGCTTTAATAAATAGTGAGGATGACTATGATATTTCAAAAAAATTTTA      4495
VroAGL11_JB_gDNA     AAATTTTAAAAATGCTTTAATAAATAGTGAGGATGACTATGATATTTCAAAAAAATTTTA      4500
VroAGL11_JB_mRNA     ------------------------------------------------------------      182
VvAGL11_CH_mRNA      ------------------------------------------------------------      182
VvAGL11_ST_mRNA      ------------------------------------------------------------      182

VvAGL11_CH_gDNA      CTAACAAAAAATGTCGATAAATCATTTATAAGTGTTATCTAAATGCCCTTAAAATCACTTA      4559
VvAGL11_ST_gDNA      CTAACAAAAAATGTCGATAAATCATTTATAAGTGTTATCTAAATGCCCTTAAAATCACTTA      4555
VroAGL11_JB_gDNA     CTAACAAAAAATGTCGATAAATCATTTATAAGTGTTATCTAAATGCCCTTAAAATCACTTA      4560
VroAGL11_JB_mRNA     ------------------------------------------------------------      182
VvAGL11_CH_mRNA      ------------------------------------------------------------      182
VvAGL11_ST_mRNA      ------------------------------------------------------------      182

VvAGL11_CH_gDNA      TTTAAATAATAGAAATTAAGCTAGATTAATAATGAATGAAAAGAAAAAAAAATAAAATTTG      4619
VvAGL11_ST_gDNA      TTTAAATAATAGAAATTAAGCTAGATTAATAATGAATGAAAAGAAAAAAAAATAAAATTTG      4615
VroAGL11_JB_gDNA     TTTAAATAATAGAAATTAAGCTAGATTAATAATGAATGAAAAGAAAAAAAAATAAAATTTG      4620
VroAGL11_JB_mRNA     ------------------------------------------------------------      182
VvAGL11_CH_mRNA      ------------------------------------------------------------      182
VvAGL11_ST_mRNA      ------------------------------------------------------------      182
```

```
VvAGL11_CH_gDNA      TACAAATCATCCAGTTTAGTACTGGTTCGAACCCCCTTCAAGCTCAAAAGGTCAATCGGA     4679
VvAGL11_ST_gDNA      TACAAATCATCCAGTTTAGTACTGGTTCGAACCCCCTTCAAGCTCAAAAGGTCAATCGGA     4675
VroAGL11_JB_gDNA     TACAAATCATCCAGTTTAGTACTGGTTCGAACCCCCTTCAAGCTCAAAAGGTCAATCGGA     4680
VroAGL11_JB_mRNA     ------------------------------------------------------------     182
VvAGL11_CH_mRNA      ------------------------------------------------------------     182
VvAGL11_ST_mRNA      ------------------------------------------------------------     182

VvAGL11_CH_gDNA      TTGGACTAGGATCTGGTCAACAGTTCGATTGATTGATCAATTCGGTCCGATCTTTAAAAC     4739
VvAGL11_ST_gDNA      TTGGACTAGGATCTGGTCAACAGTTCGATTGATTGATCAATTCGGTCCGATCTTTAAAAC     4735
VroAGL11_JB_gDNA     TTGGACTAGGATCTGGTCAACAGTTCGATTGATTGATCAATTCGGTCCGATCTTTAAAAC     4740
VroAGL11_JB_mRNA     ------------------------------------------------------------     182
VvAGL11_CH_mRNA      ------------------------------------------------------------     182
VvAGL11_ST_mRNA      ------------------------------------------------------------     182

VvAGL11_CH_gDNA      ATTACAAATAACTTATCTTATGTGAGTTTTGGTGCATCTCATTCATGCAAAGCAGTTATT     4799
VvAGL11_ST_gDNA      ATTACAAATAACTTATCTTATGTGAGTTTTGGTGCATCTCATACATGCAAAGCAGTTATT     4795
VroAGL11_JB_gDNA     ATTACAAATAACTTATCTTATGTGAGTTTTGGTGCATCTCATTCATGCAAAGCAGTTATT     4800
VroAGL11_JB_mRNA     ------------------------------------------------------------     182
VvAGL11_CH_mRNA      ------------------------------------------------------------     182
VvAGL11_ST_mRNA      ------------------------------------------------------------     182

VvAGL11_CH_gDNA      GAAAGCCAATACATATTTCATACAAAGTCCACACAAAGAAACTCAAATAAAAAAATAAAA     4859
VvAGL11_ST_gDNA      GAAAGCCAATACATATTTCATACAAAGTCCACACAAAGAAACTCAAATAAAAAAATAAAA     4855
VroAGL11_JB_gDNA     GAAAGCCAATACATATTTCATACAAAGTCCACACAAAGAAACTCAAATAAAAAAATAAAA     4860
VroAGL11_JB_mRNA     ------------------------------------------------------------     182
VvAGL11_CH_mRNA      ------------------------------------------------------------     182
VvAGL11_ST_mRNA      ------------------------------------------------------------     0182

VvAGL11_CH_gDNA      AAATCAGAGAATAAAATCCTCATGGTAAAGTTGCACCAATAAGACCTATACTGCATACAG     4919
VvAGL11_ST_gDNA      AAATCAGAGAATAAAATCCTCATGGTAAAGTTGCACCAATAAGACCTATACTGCATACAG     4915
VroAGL11_JB_gDNA     AAATCAGAGAATAAAATCCTCATGGTAAAGTTGCACCAATAAGACCTATACTGCATACAG     4920
VroAGL11_JB_mRNA     ------------------------------------------------------------     182
VvAGL11_CH_mRNA      ------------------------------------------------------------     182
VvAGL11_ST_mRNA      ------------------------------------------------------------     182

VvAGL11_CH_gDNA      TTGCAGAGTGGCAGTGCTCTTCATTGTTCTTTCTCTTGCTTTTCAGTGGTGTAATCCATG     4979
VvAGL11_ST_gDNA      TTGCAGAGTGGCAGTGCTCTTCATTGTTCTTTCTCTTGCTTTTCAGTGGTGTAATCCATG     4975
VroAGL11_JB_gDNA     TTGCAGAGTGGCAGTGCTCTTCATTGTTCTTTCTCTTGCTTTTCAGTGGTGTAATCCATG     4980
VroAGL11_JB_mRNA     ------------------------------------------------------------     182
VvAGL11_CH_mRNA      ------------------------------------------------------------     182
VvAGL11_ST_mRNA     -------------------------------------------------------------     182

VvAGL11_CH_gDNA      AGAATCTGAACCATCTGGCAGTGTCTGAAAAAGGGAGGTCATAGCAGTACAACCAACCAC     5039
VvAGL11_ST_gDNA      AGAATCTGAACCATCTGGCAGTGTCTGAAAAAGGGAGGTCATAGCAGTACAACCAACCAC     5035
VroAGL11_JB_gDNA     AGAATCTGAACCATCTGGCAGTGTCTGAAAAAGGGAGGTCATAGCAGTACAACCAACCAC     5040
VroAGL11_JB_mRNA     ------------------------------------------------------------     182
VvAGL11_CH_mRNA      ------------------------------------------------------------     182
VvAGL11_ST_mRNA      ------------------------------------------------------------     182

VvAGL11_CH_gDNA      TCATTTTTCATCTTTCCTTGTATCTTCCCTTGTTTGCAAAATCTCAGTTTTTCTGGTTGT     5099
VvAGL11_ST_gDNA      TCATTTTTCATCTTTCCTTGTATCTTCCCTTGTTTGCAAAATCTCAGTTTTTCTGGTTGT     5095
VroAGL11_JB_gDNA     TCATTTTTCATCTTTCCTTGTATCTTCCCTTGTTTGCAAAATCTCAGTTTTTCTGGTTGT     5100
VroAGL11_JB_mRNA     ------------------------------------------------------------     182
VvAGL11_CH_mRNA      ------------------------------------------------------------     182
VvAGL11_ST_mRNA      ------------------------------------------------------------     182

VvAGL11_CH_gDNA      TTCTTTCCACTTTTGGCTTTCCGCAAACTTTGAATGGAGTCTTGGGTCTTTTCTCTTTGC     5159
VvAGL11_ST_gDNA      TTCTTTCCACTTTTGGCTTTCCGCAAACTTTGAATGGAGTCTTGGGTCTTTTCTCTTTGC     5155
VroAGL11_JB_gDNA     TTCTTTCCACTTTTGGCTTTTGGCAAACTTTGAATGGAGTCTTGGGTCTTTTCTCTTTGC     5160
VroAGL11_JB_mRNA     ------------------------------------------------------------     182
VvAGL11_CH_mRNA      ------------------------------------------------------------     182
VvAGL11_ST_mRNA      ------------------------------------------------------------     182

VvAGL11_CH_gDNA      CTCTCAAAGCTGCAATCACTGCTCTTTTTTAGTGCTGGAGAGGAGAAACCAGGACAAGAC     5219
VvAGL11_ST_gDNA      CTCTCAAAGCTGCAATCACTGCTCTTTTTTAGTGCTGGAGAGGAGAAACCAGGACAAGAC     5215
VroAGL11_JB_gDNA     CTCTCAAAGCTGCAATAACTGCTCTCTTTTAGTGCTGGAGAGTAGAAACCAAGACAAGAC     5220
VroAGL11_JB_mRNA     ------------------------------------------------------------     182
VvAGL11_CH_mRNA      ------------------------------------------------------------     182
VvAGL11_ST_mRNA      ------------------------------------------------------------     182

VvAGL11_CH_gDNA      TTTTCAACTGCTATTCTCCATACAAACTCATGAAACTGATGAACAATTGAGCAGTAGGGT     5279
VvAGL11_ST_gDNA      TTTTCAACTGCTATTCTCCATACAAACTCATGAAACTGATGAACAATTGAGCAGTAGGGT     5275
VroAGL11_JB_gDNA     TTTTCAACTGCTATTCTCCATACAAGCTCATGAAACTGATGAAGAATTGAGTAGTAGTGT     5280
VroAGL11_JB_mRNA     ------------------------------------------------------------     182
VvAGL11_CH_mRNA      ------------------------------------------------------------     182
VvAGL11_ST_mRNA      ------------------------------------------------------------     182
```

```
VvAGL11_CH_gDNA      CACTGTGTATACTGTATAGTGATTTTAATCATTTTTATGTCTCATAACTTGTGGGTGTTT      5339
VvAGL11_ST_gDNA      CACTGTGTATACTGTATAGTGATTTTAATCATTTTTATGTCTCATAACTTGTGGGTGTTT      5335
VroAGL11_JB_gDNA     CACTGTGTATACTGTATAGTGATTTTAATCATTTTTATGTCTCATAACTTGTGGGTGTTT      5340
VvAGL11_CH_mRNA      ------------------------------------------------------------      182
VroAGL11_JB_mRNA     ------------------------------------------------------------      182
VvAGL11_ST_mRNA      ------------------------------------------------------------      182

VvAGL11_CH_gDNA      GTACTGTTACTTCATCTTTCTCATATCAAGTCATAGTCCTTTCACAAACTTGTGCTGTCA      5399
VvAGL11_ST_gDNA      GTACTGTTACTTCATCTTTCTCATATCAAGTCATAGTCCTTTCACAAACTTGTGCTGTCA      5395
VroAGL11_JB_gDNA     GTACTGTTGCTTCATCTTTCTCATATCAAGTCATAGACCTTTCACAAACTTGTGCTGTCA      5400
VroAGL11_JB_mRNA     ------------------------------------------------------------      182
VvAGL11_CH_mRNA      ------------------------------------------------------------      182
VvAGL11_ST_mRNA      ------------------------------------------------------------      182

VvAGL11_CH_gDNA      TCTCCCATCATATATGGTTTCCTCCTTTAGGGTTTTGCTTTCCGCCTTCACTTTTGACTA      5459
VvAGL11_ST_gDNA      TCTCCCATCATATATGGTTTCCTCCTTTAGGGTTTTGCTTTCCGCCTTCACTTTTGACTA      5455
VroAGL11_JB_gDNA     TCTCCCATCATATAAGGTTTCCTCCTTCAGGGTTTTGCTTTCCCCCCCCACTTTTGACTA      5460
VroAGL11_JB_mRNA     ------------------------------------------------------------      182
VvAGL11_CH_mRNA      ------------------------------------------------------------      182
VvAGL11_ST_mRNA      ------------------------------------------------------------      182

VvAGL11_CH_gDNA      CTTTTGGAGCAATTTTCTCTCTTGAATTCTATGGTATGTAATATTTCAATCCATTTTGCA      5519
VvAGL11_ST_gDNA      CTTTTGGAGCAATTTTCTCTCTTGAATTCTATGGTATGTAATATTTCAATCCATTTTGCA      5515
VroAGL11_JB_gDNA     CTTTTGGAGCAATTTTCTCTCTTGAATTCTATGGTATGTAATATTTCAATCCATTTTGCA      5520
VroAGL11_JB_mRNA     ------------------------------------------------------------      182
VvAGL11_CH_mRNA      ------------------------------------------------------------      182
VvAGL11_ST_mRNA      ------------------------------------------------------------      182

VvAGL11_CH_gDNA      ACAATTTTTTTAATTCATTCTATTTTTATATATTGGAAAACAGCATAAAATCAACCATAG      5579
VvAGL11_ST_gDNA      ACAATTTTTTTAATTCATTCTATTTTTATATATTGGAAAACAGCATAAAATCAACCATAG      5575
VroAGL11_JB_gDNA     ACAATTTTTTTAATTCATTCTATTTTTATATATTGGAAAACAGCATAAAATCAACCATAG      5580
VroAGL11_JB_mRNA     -------------------------------------------CATAAAATCAACCATAG      199
VvAGL11_CH_mRNA      -------------------------------------------CATAAAATCAACCATAG      199
VvAGL11_ST_mRNA      -------------------------------------------CATAAAATCAACCATAG      199
                                                                *****************

VvAGL11_CH_gDNA      ATAGGTACAAGAAGGCCAGCTCAGATAGTACAAATGGAGGCTCTACCATGGAGATCAATG      5639
VvAGL11_ST_gDNA      ATAGGTACAAGAAGGCCAGCTCAGATAGTACAAATGGAGGCTCTACCATGGAGATCAATG      5635
VroAGL11_JB_gDNA     ATAGGTACAAGAAGGCCAGCTCAGATAGTACAAATGCAGGCTCTACCATGGAGATCAATG      5640
VroAGL11_JB_mRNA     ATAGGTACAAGAAGGCCAGCTCAGATAGTACAAATGCAGGCTCTACCATGGAGATCAATG      259
VvAGL11_CH_mRNA      ATAGGTACAAGAAGGCCAGCTCAGATAGTACAAATGGAGGCTCTACCATGGAGATCAATG      259
VvAGL11_ST_mRNA      ATAGGTACAAGAAGGCCAGCTCAGATAGTACAAATGGAGGCTCTACCATGGAGATCAATG      259
                     ************************************ ***********************

VvAGL11_CH_gDNA      CCCAAGTAAGAAAACTTCCATTTTATGATAGTTAATGAATCAATCCAAATCCTATATGGT      5699
VvAGL11_ST_gDNA      CCCAAGTAAGAAAACTTCCATTTTATGATAGTTAATGAATCAATCCAAATCCTATATGGT      5695
VroAGL11_JB_gDNA     CCCAAGTAAGAAAACTTCCATTTTATGATAGTTAATGAATCAATCCAAATCCTATATGGT      5700
VroAGL11_JB_mRNA     CCCAA-------------------------------------------------------      264
VvAGL11_CH_mRNA      CCCAA-------------------------------------------------------      264
VvAGL11_ST_mRNA      CCCAA-------------------------------------------------------      264
                     *****

VvAGL11_CH_gDNA      TTACTGATCAGATGTCTTATCCACCATGGCTGTTAGAACATCTCAATCCATTCTGTGGAT      5759
VvAGL11_ST_gDNA      TTACTGATCAGATGTCTTATCCACCATGGCTGTTAGAACATCTCAATCCATTCTGTGGAT      5755
VroAGL11_JB_gDNA     TTACTGATCAGATGTCTTATCCACCATGGCTGTTAGAACATCTCAATCCATTCTGTGGAT      5760
VroAGL11_JB_mRNA     ------------------------------------------------------------      264
VvAGL11_CH_mRNA      ------------------------------------------------------------      264
VvAGL11_ST_mRNA      ------------------------------------------------------------      264

VvAGL11_CH_gDNA      TGAAAAGTGCACATTTTTCATTAGGCTGGCCACAAATTAAAACCCTCATCATGCTTAGCC      5819
VvAGL11_ST_gDNA      TGAAAAGTGCACATTTTTCATTAGGCTGGCCACAAATTAAAACCCTCATCATGCTTAGCC      5815
VroAGL11_JB_gDNA     TGAAAAGTGCACATTTTTCATTAGGCTGGCCACAAATTAAAACCCTCATCATGCTTAGCC      5820
VroAGL11_JB_mRNA     ------------------------------------------------------------      264
VvAGL11_CH_mRNA      ------------------------------------------------------------      264
VvAGL11_ST_mRNA      ------------------------------------------------------------      264

VvAGL11_CH_gDNA      ATAAACAAATATGTTAAAATCATTATCCTACTTTCTTTTTTCTTTTTGGATTATCCCATT      5879
VvAGL11_ST_gDNA      ATAAACAAATATGTTAAAATCATTATCCTACTTTCTTTTTTCTTTTTGGATTATCCCATT      5875
VroAGL11_JB_gDNA     ATAAACAAATATGTTAAAATCATTATCCTACTTTCTTTTTTCTTTTTGGATTATCCCATT      5880
VroAGL11_JB_mRNA     ------------------------------------------------------------      264
VvAGL11_CH_mRNA      ------------------------------------------------------------      264
VvAGL11_ST_mRNA      ------------------------------------------------------------      264

VvAGL11_CH_gDNA      GAACTTTCTCGAATTCTGTTGTTGAACATAGTACTACCAGCAAGAATCAGCAAAGCTGCG      5939
VvAGL11_ST_gDNA      GAACTTTCTCGAATTCTGTTGTTGAACATAGTACTACCAGCAAGAATCAGCAAAGCTGCG      5935
VroAGL11_JB_gDNA     GAACTTTCTCGAATTCTGTTGTTGAACATAGTATTACCAGCAAGAATCAGCAAAGCTGCG      5940
VroAGL11_JB_mRNA     -------------------------------TATTACCAGCAAGAATCAGCAAAGCTGCG      293
VvAGL11_CH_mRNA      -------------------------------TACTACCAGCAAGAATCAGCAAAGCTGCG      293
VvAGL11_ST_mRNA      -------------------------------TACTACCAGCAAGAATCAGCAAAGCTGCG      293
                                                    ** **************************
```

```
VvAGL11_CH_gDNA      CCAGCAAATACAGATGCTGCAGAATTCTAACAGGTACCCTTGATTCATATTTCATTTTTC      5999
VvAGL11_ST_gDNA      CCAGCAAATACAGATGCTGCAGAATTCTAACAGGTACCCTTGATTCATATTTCATTTTTC      5995
VroAGL11_JB_gDNA     CCAGCAAATACAGATGCTGCAGAATTCTAACAGGTACCCTTGATTCATATTTCATTTTTC      6000
VroAGL11_JB_mRNA     CCAGCAAATACAGATGCTGCAGAATTCTAACAG---------------------------      326
VvAGL11_CH_mRNA      CCAGCAAATACAGATGCTGCAGAATTCTAACAG---------------------------      326
VvAGL11_ST_mRNA      CCAGCAAATACAGATGCTGCAGAATTCTAACAG---------------------------      326
                     *********************************

VvAGL11_CH_gDNA      TCACATGCAGTTGAGTGTATAT----ATATATGTCAATTTGCATTTTCCTTCTGCTTGTG      6055
VvAGL11_ST_gDNA      TCACATGCAGTTGAGTGTATAT----ATATATGTCAATTTGCATTTTCCTTCTGCTTGTG      6051
VroAGL11_JB_gDNA     TCAAATGTACTTGAGTATATATATATATATATATCAATTTGCATTTTCCTTCTGCTTGTG      6060
VroAGL11_JB_mRNA     ------------------------------------------------------------      326
VvAGL11_CH_mRNA      ------------------------------------------------------------      326
VvAGL11_ST_mRNA      ------------------------------------------------------------      326

VvAGL11_CH_gDNA      TCTTTAACTAGGTTTTTGACATCAGTTCCCATGCAAAAATTTCAAATATTACTTAACGAT      6115
VvAGL11_ST_gDNA      TCTTTAACTAGGTTTTTGACATCAGTTCCCATGCAAAAATTTCAAATATTACTTAACGAT      6111
VroAGL11_JB_gDNA     TCTTTAACTATGTTTTTGACATCAGTTCCCATGCAAAAATAAAAAATATAACTTAAAGAT      6120
VroAGL11_JB_mRNA     ------------------------------------------------------------      326
VvAGL11_CH_mRNA      ------------------------------------------------------------      326
VvAGL11_ST_mRNA      ------------------------------------------------------------      326

VvAGL11_CH_gDNA      CTTAATTAGATATGTTAAGACTTAAGATCAGGTTGTACTAAGACATTTTGTCTGTGTTTA      6175
VvAGL11_ST_gDNA      CTTAATTAGATATGTTAAGACTTAAGATCAGGTTGTACTAAGACATTTTGTCTGTGTTTA      6171
VroAGL11_JB_gDNA     CTAAATTANGTATGTGTAGACTTAAAAGCAGGTTGTACTAAGACATTTTGTCTCTGTTTA      6180
VroAGL11_JB_mRNA     ------------------------------------------------------------      326
VvAGL11_CH_mRNA      ------------------------------------------------------------      326
VvAGL11_ST_mRNA      ------------------------------------------------------------      326

VvAGL11_CH_gDNA      TAGGCACTTAATGGGTGATTCCTTGGCTTCCTTGACTGTGAAGGAGCTAAAGCAGCTCGA      6235
VvAGL11_ST_gDNA      TAGGCACTTAATGGGTGATTCCTTGGCTTCCTTGACTGTGAAGGAGCTAAAGCAGCTCGA      6231
VroAGL11_JB_gDNA     TATGCACTTAATGGGTGATTCCTTGGCTTCCTTGACTGTGAAGGAGCTCAAGCAGCTCGA      6240
VroAGL11_JB_mRNA     ---GCACTTAATGGGTGATTCCTTGGCTTCCTTGACTGTGAAGGAGCTCAAGCAGCTCGA      383
VvAGL11_CH_mRNA      ---GCACTTAATGGGTGATTCCTTGGCTTCCTTGACTGTGAAGGAGCTAAAGCAGCTCGA      383
VvAGL11_ST_mRNA      ---GCACTTAATGGGTGATTCCTTGGCTTCCTTGACTGTGAAGGAGCTAAAGCAGCTCGA      383
                        ********************************************* ***********

VvAGL11_CH_gDNA      GAACAGGCTTGAACGAGGCATCACAAGAATCAGGTCGAAGAAGGTAACGACACCTAGACA      6295
VvAGL11_ST_gDNA      GAACAGGCTTGAACGAGGCATCACAAGAATCAGGTCGAAGAAGGTAACGACACCTAGACA      6291
VroAGL11_JB_gDNA     GAACAGGCTTGAACGAGGCATCACAAGAATCAGGTCGAAGAAGGTAACGACACCTAGACA      6300
VroAGL11_JB_mRNA     GAACAGGCTTGAACGAGGCATCACAAGAATCAGGTCGAAGAAG-----------------      426
VvAGL11_CH_mRNA      GAACAGGCTTGAACGAGGCATCACAAGAATCAGGTCGAAGAAG-----------------      426
VvAGL11_ST_mRNA      GAACAGGCTTGAACGAGGCATCACAAGAATCAGGTCGAAGAAG-----------------      426
                     *******************************************

VvAGL11_CH_gDNA      CTAAATCTAATCTGGCTGAGCATTTGATTTTGAGACTTAAATGAAATGGAAAAAAAAATG      6355
VvAGL11_ST_gDNA      CTAAATCTAATCTGGCTGAGCATTTGATTTTGAGACTTAAATGAAATGGAAAAAAAAATG      6351
VroAGL11_JB_gDNA     CTAAATCTAATCTGGCTGAGCATTTGATTTTGAGACTTAAATGAAATGGAAAAAAAAATG      6360
VroAGL11_JB_mRNA     ------------------------------------------------------------      426
VvAGL11_CH_mRNA      ------------------------------------------------------------      426
VvAGL11_ST_mRNA      ------------------------------------------------------------      426

VvAGL11_CH_gDNA      GTATCATTCACTGATTAAATAATAATACTTGAAGGTTGAATTAATTTAAGATACCGCTTT      6415
VvAGL11_ST_gDNA      GTATCATTCACTGATTAAATAATAATACTTGAAGGTTGAATTAATTTAAGATACCGCTTT      6411
VroAGL11_JB_gDNA     GTATCATTCACTGATTAAATAATAATACTTGAAGGTTGAATTAATTTAAGATACCGCTTT      6420
VroAGL11_JB_mRNA     ------------------------------------------------------------      426
VvAGL11_CH_mRNA      ------------------------------------------------------------      426
VvAGL11_ST_mRNA      ------------------------------------------------------------      426

VvAGL11_CH_gDNA      CACTTGATAACTAAAGCCTGATGGTAGGAACTTCCACCCATCTAGCTTAGTTGGTGATTG      6475
VvAGL11_ST_gDNA      CACTTGATAACTAAAGCCTGATGGTAGGAACTTCCACCCATCTAGCTTAGTTGGTGATTG      6411
VroAGL11_JB_gDNA     CACTTGATAACTAAAGCCTGATGGTAGGAACTTCCACCCATCTAGCTTAGTTGGTGATTG      6480
VroAGL11_JB_mRNA     ------------------------------------------------------------      426
VvAGL11_CH_mRNA      ------------------------------------------------------------      426
VvAGL11_ST_mRNA      ------------------------------------------------------------      426

VvAGL11_CH_gDNA      AGCTGAATTTTGTCTTGGAAACTTTGTGAAAAATAGACAAGTGATCACTAGATCAGCAAA      6535
VvAGL11_ST_gDNA      AGCTGAATTTTGTCTTGGAAACTTTGTGAAAAATAGACAAGTGATCACTAGATCAGCAAA      6531
VroAGL11_JB_gDNA     AGCTGAATTTTGTCTTGGAAACTTTGTGAAAAATAGACAAGTGATCACTAGATCAGCAAA      6540
VroAGL11_JB_mRNA     ------------------------------------------------------------      426
VvAGL11_CH_mRNA      ------------------------------------------------------------      426
VvAGL11_ST_mRNA      ------------------------------------------------------------      426

VvAGL11_CH_gDNA      GCCTATCTTTTTTCCTTTGTTCTAATGTCGTGCTTGTTCTTATGCAATAGCATGAGTTGC      6595
VvAGL11_ST_gDNA      GCCTATCTTTTTTCCTTTGTTCTAATGTCGTGCTTGTTCTTATGCAATAGCATGAGTTGC      6591
VroAGL11_JB_gDNA     GCCTATCTTTTTTCCTTTGTTCTAATGTCGTGCTTGTTCTTATGCAATAGCATGAGTTGC      6600
VroAGL11_JB_mRNA     --------------------------------------------------CATGAGTTGC      436
VvAGL11_CH_mRNA      --------------------------------------------------CATGAGTTGC      436
VvAGL11_ST_mRNA      --------------------------------------------------CATGAGTTGC      436
                                                                       **********
```

```
VvAGL11_CH_gDNA       TGTTGGCTGAGATTGAGTACTTGCAGAAAAGGGTAATCTCTTGTTCTAAACATCAATTTC         6655
VvAGL11_ST_gDNA       TGTTGGCTGAGATTGAGTACTTGCAGAAAAGGGTAATCTCTTGTTCTAAACATCAATTTC         6651
VroAGL11_JB_gDNA      TGTTGGCTGAGATTGAATACTTGCAGAAAAGGGTAATCTCTTGTTCTAAACATCAATTTC         6660
VroAGL11_JB_mRNA      TGTTGGCTGAGATTGAATACTTGCAGAAAAGG----------------------------         468
VvAGL11_CH_mRNA       TGTTGGCTGAGATTGAGTACTTGCAGAAAAGG----------------------------         468
VvAGL11_ST_mRNA       TGTTGGCTGAGATTGAGTACTTGCAGAAAAGG----------------------------         468
                      **************** ***************

VvAGL11_CH_gDNA       CATGTGGGTTCCCTTCTTTTATGATTTTTCCAGAATTCTTTATCGAGCACCTTTTAATTT         6715
VvAGL11_ST_gDNA       CATGTGGGTTCCCTTCTTTTATGATTTTTCCAGAATTCTTTATCGAGCACCTTTTAATTT         6711
VroAGL11_JB_gDNA      CATGTGGGTTCCCTTCTTTTATGATTTTGCCAGAATTCTTTATCGACCACGTGTTAATTT         6720
VroAGL11_JB_mRNA      ------------------------------------------------------------         468
VvAGL11_CH_mRNA       ------------------------------------------------------------         468
VvAGL11_ST_mRNA       ------------------------------------------------------------         468

VvAGL11_CH_gDNA       TCTTAGGAAATTGAGCTGGAAAATGAAAGCGTATATCTCCGAACCAAGGTATGAATTAAA         6775
VvAGL11_ST_gDNA       TCTTAGGAAATTGAGCTGGAAAATGAAAGCGTATATCTCCGAACCAAGGTATGAATTAAA         6771
VroAGL11_JB_gDNA      CCTTAGGAAATTGAGCTGGAAAATGAAAGCGTATACCTCCGAACCAAGGTAGGAATTAAA         6780
VroAGL11_JB_mRNA      ------GAAATTGAGCTGGAAAATGAAAGCGTATACCTCCGAACCAAG------------         510
VvAGL11_CH_mRNA       ------GAAATTGAGCTGGAAAATGAAAGCGTATATCTCCGAACCAAG------------         510
VvAGL11_ST_mRNA       ------GAAATTGAGCTGGAAAATGAAAGCGTATATCTCCGAACCAAG------------         510
                            ***************************** ************

VvAGL11_CH_gDNA       TTAGTATAGGAATTGAATGTGATAAAATTGAAGGCACAACAGTTGATACCGATCTAGCAG         6835
VvAGL11_ST_gDNA       TTAGTATAGGAATTGAATGTGATAAAATTGAAGGCACAACAGTTGATACCGATCTAGCAG         6831
VroAGL11_JB_gDNA      TTACTATAGGAATTGAATGTGATAATATTGAAGGCACAACAGTTGATACCGATCTAGCAG         6840
VroAGL11_JB_mRNA      ------------------------------------------------------------         510
VvAGL11_CH_mRNA       ------------------------------------------------------------         510
VvAGL11_ST_mRNA       ------------------------------------------------------------         510

VvAGL11_CH_gDNA       TTCAGTTGAACTTGTTAGTGTATATATTTAACAATTGATTGAGCTATCATTACTTAATTA         6895
VvAGL11_ST_gDNA       TTCAGTTGAACTTGTTAGTGTATATATTTAACAATTGATTGAGCTATCATTACTTAATTA         6891
VroAGL11_JB_gDNA      TTCAGTTGAACTTGTTAGTGTATATATTTAACAATTGATTGAGCTATCATTACTTAATTA         6900
VroAGL11_JB_mRNA      ------------------------------------------------------------         510
VvAGL11_CH_mRNA       ------------------------------------------------------------         510
VvAGL11_ST_mRNA       ------------------------------------------------------------         510

VvAGL11_CH_gDNA       TTTTGATGTATGAAAACTATCCATATCCATACTACTACTCTTCATTGAGCTCTGCTGCAC         6955
VvAGL11_ST_gDNA       TTTTGATGTATGAAAACTATCCATATCCATACTACTACTCTTCATTGAGCTCTGCTGCAC         6951
VroAGL11_JB_gDNA      TTTTGATGTATGAAAACTATCCATATCCATACTACTACTCTTCTTG-AGCTCTGCTGCAC         6960
VroAGL11_JB_mRNA      ------------------------------------------------------------         510
VvAGL11_CH_mRNA       ------------------------------------------------------------         510
VvAGL11_ST_mRNA       ------------------------------------------------------------         510

VvAGL11_CH_gDNA       TAAACTTTCAGATTGCAGAAGTGGAGAGGCTTCAGCAAGCAAACATGGTATCAACACATG         7015
VvAGL11_ST_gDNA       TAAACTTTCAGATTGCAGAAGTGGAGAGGCTTCAGCAAGCAAACATGGTATCAACACATG         7011
VroAGL11_JB_gDNA      TAAACTTTCAGATTGCAGAAGTGGAGAGGCTTCAGCAAGCAAACATGGTATCAACACATG         7020
VroAGL11_JB_mRNA      -----------ATTGCAGAAGTGGAGAGGCTTCAGCAAGCAAACATGGTATCAACACATG         559
VvAGL11_CH_mRNA       -----------ATTGCAGAAGTGGAGAGGCTTCAGCAAGCAAACATGGTATCAACACATG         559
VvAGL11_ST_mRNA       -----------ATTGCAGAAGTGGAGAGGCTTCAGCAAGCAAACATGGTATCAACACATG         559
                                 *************************************************

VvAGL11_CH_gDNA       AGTTCAATGCCATCCAGGCATTAGTTTCTCGCAATTTCTTTCAGCCCAATATGATTGAGG         7075
VvAGL11_ST_gDNA       AGTTCAATGCCATCCAGGCATTAGTTTCTCGCAATTTCTTTCAGCCCAATATGATTGAGG         7071
VroAGL11_JB_gDNA      AGTTCAATGCCATCCAGGCATTAGTTTCTCGCAATTTCTTTCAGCCCAATATGATTGAGG         7080
VroAGL11_JB_mRNA      AGTTCAATGCCATCCAGGCATTAGTTTCTCGCAATTTCTTTCAGCCCAATATGATTGAGG         619
VvAGL11_CH_mRNA       AGTTCAATGCCATCCAGGCATTAGTTTCTCGCAATTTCTTTCAGCCCAATATGATTGAGG         619
VvAGL11_ST_mRNA       AGTTCAATGCCATCCAGGCATTAGTTTCTCGCAATTTCTTTCAGCCCAATATGATTGAGG         619
                      ************************************************************

VvAGL11_CH_gDNA       GTGGATCCACAGGCTACCCACTTCCTGATAAGAAGGTCCTCCATCTCGGGTACACTCATC         7135
VvAGL11_ST_gDNA       GTGGATCCACAGGCTACCCACTTCCTGATAAGAAGGTCCTCCATCTCGGGTACACTCATC         7131
VroAGL11_JB_gDNA      GTGGATCCACAGGCTACCCACTTCCTGATAAGAAGGTCCTTCATCTCGGGTACACTCATC         7140
VroAGL11_JB_mRNA      GTGGATCCACAGGCTACCCACTTCCTGATAAGAAGGTCCTTCATCTCGG-----------         668
VvAGL11_CH_mRNA       GTGGATCCACAGGCTACCCACTTCCTGATAAGAAGGTCCTCCATCTCGG-----------         668
VvAGL11_ST_mRNA       GTGGATCCACAGGCTACCCACTTCCTGATAAGAAGGTCCTCCATCTCGG-----------         668
                      **************************************** ********

VvAGL11_CH_gDNA       GTACACTCATCTTTCGCCATGTTTATTCATTTGCATTCTTTCTCGGTCTAATATTGAAGA         7195
VvAGL11_ST_gDNA       GTACACTCATCTTTCGCCATGTTTATTCATTTGCATTCTTTCTCGGTCTAATATTGAAGA         7191
VroAGL11_JB_gDNA      GTACACTCATCTTTCGCCATGTTTATTCATTTGCATTCTTTCTCGGTCTAATATTGAAGA         7200
VroAGL11_JB_mRNA      ------------------------------------------------------------         668
VvAGL11_CH_mRNA       ------------------------------------------------------------         668
VvAGL11_ST_mRNA       ------------------------------------------------------------         668

VvAGL11_CH_gDNA       TTAAAAGAGCACATAAATCACCAAGTAGCCTTAGGTCTGCTAAATTCATCACTACAAATG         7255
VvAGL11_ST_gDNA       TTAAAAGAGCACATAAATCACCAAGTAGCCTTAGGTCTGCTAAATTCATCACTACAAATG         7251
VroAGL11_JB_gDNA      TTAAAAGAGCACATNNNNNNNNNNNNNNNNNNNNNNNNN---------------------         7214
VroAGL11_JB_mRNA      ------------------------------------------------------------         668
VvAGL11_CH_mRNA       ------------------------------------------------------------         668
VvAGL11_ST_mRNA       ------------------------------------------------------------         668
```

```
VvAGL11_CH_gDNA       GTATATGCATGAACAGCTTAATGAGACATTTCAAGCCATGCATTGGGTAGAAAAAAGTTG         7255
VvAGL11_ST_gDNA       GTATATGCATGAACAGCTTAATGAGACATTTCAAGCCATGCATTGGGTAGAAAAAAGTTG         7251
VroAGL11_JB_gDNA      ------------------------------------------------------------         7214
VroAGL11_JB_mRNA      ------------------------------------------------------------         668
VvAGL11_CH_mRNA       ------------------------------------------------------------         668
VvAGL11_ST_mRNA       ------------------------------------------------------------         668

VvAGL11_CH_gDNA       TGAGATTTCTTCGTCTTTTAAAATACTGCATAGTCATTGACTACCCTATATAATCTTGCT         7315
VvAGL11_ST_gDNA       TGAGATTTCTTCGTCTTTTAAAATACTGCATAGTCATTGACTACCCTATATAATCTTGCT         7351
VroAGL11_JB_gDNA      ------------------------------------------------------------         7214
VroAGL11_JB_mRNA      ------------------------------------------------------------         668
VvAGL11_CH_mRNA       ------------------------------------------------------------         668
VvAGL11_ST_mRNA       ------------------------------------------------------------         668

VvAGL11_CH_gDNA       GTGAATTATATACTAATTATGATGGGCAAAACAAAAATTTGTTCTCCAAATCCTCCTCAA         7375
VvAGL11_ST_gDNA       GTGAATTATATACTAATTATGATGGGCAAAACAAAAATTTGTTCTCCAAATCCTCCTCAA         7371
VroAGL11_JB_gDNA      ------------------------------------------------------------         7214
VroAGL11_JB_mRNA      ------------------------------------------------------------         668
VvAGL11_CH_mRNA       ------------------------------------------------------------         668
VvAGL11_ST_mRNA       ------------------------------------------------------------         668

VvAGL11_CH_gDNA       GTTCCTGTGATGTGTATGAGGGCCTAAAGATGATTGTTCATCTGGGCATTTCGATTTTTG         7375
VvAGL11_ST_gDNA       GTTCCTGTGATGTGTATGAGGGCCTAAAGATGATTGTTCATCTGGGCATTTCGATTTTTG         7371
VroAGL11_JB_gDNA      ------------------------------------------------------------         7214
VroAGL11_JB_mRNA      ------------------------------------------------------------         668
VvAGL11_CH_mRNA       ------------------------------------------------------------         668
VvAGL11_ST_mRNA       ------------------------------------------------------------         668

VvAGL11_CH_gDNA       CTCATCCTTAATAATCAGTACTTCTCCAAAGTAAGTAGATTTCCATGTTTTCTTTTGGCT         7435
VvAGL11_ST_gDNA       CTCATCCTTAATAATCAGTACTTCTCCAAAGTAAGTAGATTTCCATGTTTTCTTTTGGCT         7431
VroAGL11_JB_gDNA      ------------------------------------------------------------         7214
VroAGL11_JB_mRNA      ------------------------------------------------------------         668
VvAGL11_CH_mRNA       ------------------------------------------------------------         668
VvAGL11_ST_mRNA       ------------------------------------------------------------         668

VvAGL11_CH_gDNA       TACAATAAGATTCAAGTTCGACCTAATTCCTGGGGTTTTTTGCTTTTTGTTTTATGTAGG         7495
VvAGL11_ST_gDNA       TACAATAAGATTCAAGTTCGACCTAATTCCTGGGGTTTTTTGCTTTTTGTTTTATGTAGG         7491
VroAGL11_JB_gDNA      --------------------------------------------------------TAGG         7218
VroAGL11_JB_mRNA      -----------------------------------------------------------G         669
VvAGL11_CH_mRNA       -----------------------------------------------------------G         669
VvAGL11_ST_mRNA       -----------------------------------------------------------G         669
                                                                                 *

VvAGL11_CH_gDNA       TAAATGATGGGAGAAAATATCCGCCAACTTC         7526
VvAGL11_ST_gDNA       TAAATGATGGGAGAAAATATCCGCCAACTTC         7522
VroAGL11_JB_gDNA      TAAATGATGGGAGAAAATATCCGCCAAGTTC         7249
VroAGL11_JB_mRNA      TAA----------------------------         672
VvAGL11_CH_mRNA       TAA----------------------------         672
VvAGL11_ST_mRNA       TAA----------------------------         672
                      ***
```

Figure S1: Multiple alignment of the genomic sequences of AGL11 gene of muscadine (cv. Jane Bell) and Bunch grape (cvs. Chardonnay, Sultanina) cultivars along with their corresponding mRNAs. The red color sequences are the 'exons' of the AGL11 gene. JB=Jane Bell, CH=Chardonnay, and ST=Sultanina.

Figure S2

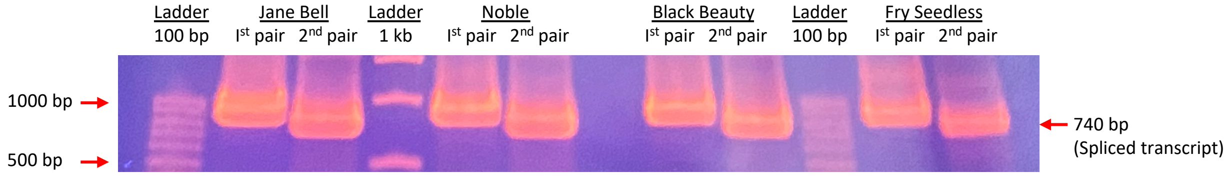

Figure S2: Gel picture showing the size of conservatively spliced transcripts (740 bp) of *AGL11* gene in muscadine cultivars Jane Bell, Noble, Black Beauty and Fry Seedless. Two pairs of gene-specific primers were used for the nested PCR (Table S1).

Figure S3

```
CLUSTAL O(1.2.4) multiple sequence alignment

Radish          ----------------------------------------MGRGKIEIKRIENSTNRQVT     20
Cabbage         ---------------------------------------MIGRGKIEIKRIENSTNRQVT     21
Rapeseed        ----------------------------------------MGRGKIEIKRIENSTNRQVT     20
Turnip          -----------------------------------MMLKEMGRGKIEIKRIENSTNRQVT     25
C_rubella       -----------MCTIITSVS-------CASVPSSSSISIWMGRGKIEIKRIENSTNRQVT     42
Camelina        MKYFPFFFNTFFCLMLFPHERNRERKKDH-----KGLRIRMGRGKIEIKRIENSTNRQVT     55
Arabidopsis     --------------MLFPHERKKEKERSQGFYLVTRLRIRMGRGKIEIKRIENSTNRQVT     46
T_Gourd         ----------------------------------------MGRGKIEIKRIENSTNRQVT     20
W_Strawberry    ----------------------------------------MGRGKIEIKRIENTTNRQVT     20
C_Rose          ----------------------------------------MGRGKIEIKRIENTTNRQVT     20
W_Lupine        ----------------------------------MEWNEEMGRGKIEIKRIENTTNRQVT     26
B_Lupine        --------------MLFTHSISN----LLLSFSFKMETQEMGRGKIEIKRIENTTNRQVT     42
WildPeanut      ----------------------------------------MGRGKIEIKRIENTTNRQVT     20
Peanut          ----------------------------------------MGRGKIEIKRIENTTNRQVT     20
Soybean         ----------------------------------------MGRGKIEIKRIENTTNRQVT     20
PigeonPea       ----------------------------------------MGRGKIEIKRIENTTNRQVT     20
CommonBean      ----------------------------------------MGRGKIEIKRIENTTNRQVT     20
Chickpea        ----------------------------------------MGRGKIEIKRIENTTNRQVT     20
Medicago        ----------------------------------------MGRGKIEIKRIENTTNRQVT     20
Peach           ----------------------------------------MGRGKIEIKRIENTTNRQVT     20
J_Apricot       ----------------------------------------MGRGKIEIKRIENTTNRQVT     20
Almond          ----------------------------------------MGRGKIEIKRIENTTNRQVT     20
S_Cherry        ----------------------------------------MGRGKIEIKRIENTTNRQVT     20
A_Pear          ----------------------------------------MGRGKIEIKRIENTTNRQVT     20
Apple           ----------------------------------------MGRGKIEIKRIENTTNRQVT     20
C_Pear          ----------------------------------------MGRGKIEIKRIENTTNRQVT     20
Pomegranate     ----------------------------------------MGRGKIEIKRIENTTNRQVT     20
Pt_Poplar       -------------------------------MRGKIQEKDMGRGKIEIKRIENTTNRQVT     29
Pe_Poplar       ----------------------------------------MGRGKIEIKRIENTTNRQVT     20
Pistachio       ----------------------------------------MGRGKIEIKRIENTTNRQVT     20
Cucumber        ----------------------------------------MGRGKIEIKRIENTTNRQVT     20
Pumpkin         ----------------------------------------MGRGKIEIKRIENTTNRQVT     20
B_Melon         ----------------------------------------MGRGKIEIKRIENTTNRQVT     20
MGrape_BB       ----------------------------------------MGRGKIEIKRIENTTNRQVT     20
BGrape_RL       ----------------------------------------MGRGKIEIKRIENTTNRQVT     20
MGrape_JB       ----------------------------------------MGRGKIEIKRIENTTNRQVT     20
MGrape_F        ----------------------------------------MGRGKIEIKRIENTTNRQVT     20
MGrape_FS       ----------------------------------------MGRGKIEIKRIENTTNRQVT     20
BGrape_RS       ----------------------------------------MGRGKIEIKRIENTTNRQVT     20
HGrape_BDB      ----------------------------------------MGRGKIEIKRIENTTNRQVT     20
BGrape_CH       ----------------------------------------MGRGKIEIKRIENTTNRQVT     20
BGrape_ST       ----------------------------------------MGRGKIEIKRIENTTNRQVT     20
Wild_grape      ----------------------------------MLCVVNMGRGKIEIKRIENTTNRQVT     26
Eucalyptus      ----------------------------------------MGRGKIEIKRIENTTNRQVT     20
W_Myrtle        ----------------------------------------MGRGKIEIKRIENTTNRQVT     20
Gb_Cotton       ----------------------------------------MGRGKIEIKRIENTTNRQVT     20
Clementine      ----------------------------------------MGRGKIEIKRIENTTNRQVT     20
Orange          ---------------------------------MSVFISPMGRGKIEIKRIENTTNRQVT     27
CastorBean      --------------------------------MIFSSEVAMGRGKIEIKRIENTTNRQVT     28
RubberTree      ----------------------M----AYIHLFYVSFEVGMGRGKIEIKRIENTTNRQVT     34
Cassava         ----------------------------------------MGRGKIEIKRIENTTNRQVT     20
Jatropha        ----------------------------------------MGRGKIEIKRIENTTNRQVT     20
Gh_Cotton       ----------------------------------------MGRGKIEIKRIENTTNRQVT     20
Durian          ----------------------------------------MGRGKIEIKRIENTTNRQVT     20
Cocoa           ----------------------------------------MGRGKIEIKRIENTTNRQVT     20
Rye             ---------------------------------------------RAIRRIESAAARQVT     15
I_Rice          ----------------------------------------MARERREIKRIESAAARQVT     20
J_Rice          ----------------------------------------MARERREIKRIESAAARQVT     20
Rice            ----------------------------------------MARERREIKRIESAAARQVT     20
Maize           ----------------------------------------MARERREIKRIESAAARQVT     20
F_Millet        ----------------------------------------MARERREIKRIESAAARQVT     20
Sorghum         ----------------------------------------MARERREIKRIESAAARQVT     20
P_Ryegrass      ----------------------------------------MARERREIKRIESAAARQVT     20
B_Wheat         ----------------------------------------MARERREIKRIESAAARQVT     20
D_Wheat         ----------------------------------------MARERREIKRIESAAARQVT     20
Barley          ----------------------------------------MARERREIKRIESAAARQVT     20
AO_palm         ----------------------------------------MVREKIQIRKIDNATARQVT     20
WM_Banana       ----------------------------------------MVREKIQIKKIDNTAARQVT     20
W_Banana        ----------------------------------------MVREKIQIKKIDNTAARQVT     20
                                                               *::*:.:: ****
```

```
Radish            FCKRRNGLLKKAYELSVLCDAEVALIVFSTRGRLYEYANN-NIRSTIERYKKA-SDNTNT     78
Cabbage           FCKRRNGLLKKAYELAVLCDAEVALIVFSTRGRLYEYAND-NIRATFERYKNSSSGNANT     80
Rapeseed          FCKRRNGLLKKAYELAVLCDAEVALIVFSTRGRLYEYGNN-NIRATIERYKKASSDNANT     79
Turnip            FCKRRNGLLKKAYELAVLCDAEVALIVFSTRGRLYEYGNN-NIRATIERYKKASSDNANT     84
C_rubella         FCKRRNGLLKKAYELSVLCDAEVALIVFSTRGRLYEYANN-NIRSTIERYKKACSDSTNT     101
Camelina          FCKRRNGLLKKAYELSVLCDAEVALIVFSTRGRLYEYANN-NIRSTIERYKKACSDSTNT     114
Arabidopsis       FCKRRNGLLKKAYELSVLCDAEVALIVFSTRGRLYEYANN-NIRSTIERYKKACSDSTNT     105
T_Gourd           FCKRRNGLLKKAYELSVLCDAEVALIVFSTRGRLYEYANN-NIRSTIERYKKACSDSTNT     79
W_Strawberry      FCKRRNGLLKKAYELSILCEAEVALIVFSSRGRLYEYSNN-NIRNTIERYKKASSDNSGA     79
C_Rose            FCKRRNGLLKKAYELSILCEAEVALIVFSSRGRLYEYSNN-NIRNTIERYKKASSDNSGA     79
W_Lupine          FCKRRNGLLKKAYELSVLCDAEVALIVFSSRGRLYEYSNN-NIRSTIDRYRKACSDNSSM     85
B_Lupine          FCKRRNGLLKKAYELSVLCDAEVALIVFSSRGRLYEYSNN-NIRSTIDRYRKACSDNSSM     101
WildPeanut        FCKRRNGLLKKAYELSVLCDAEVALIVFSSRGRLYEYSNN-NIRSTIERYKKACSDHSCT     79
Peanut            FCKRRNGLLKKAYELSVLCDAEVALIVFSSRGRLYEYSNN-NIRSTIERYKKACSDHSCT     79
Soybean           FCKRRNGLLKKAYELSVLCDAEVALIVFSSRGRLYEYSNN-NIRSTIERYKKACSDHSSA     79
PigeonPea         FCKRRNGLLKKAYELSVLCDAEVALIVFSSRGRLYEYSNN-NIRSTIERYKKASSDHSST     79
CommonBean        FCKRRNGLLKKAYELSVLCDAEVALIVFSSRGRLYEYSNN-NIRSTIERYKKACSDHSST     79
Chickpea          FCKRRNGLLKKAYELSVLCDAEVALIVFSSRGRLYEYSNN-NIRSTIDRYKKACSDHSST     79
Medicago          FCKRRNGLLKKAYELSVLCDAEVALIVFSSRGRLYEYSNN-NIRSTIDRYKKACSDHSST     79
Peach             FCKRRNGLLKKAYELSILCDAEVALIVFSSRGRLYEYSNNNSIRNTIERYKKACSDSSGS     80
J_Apricot         FCKRRNGLLKKAYELSILCDAEVALIVFSSRGRLYEYSNN-NIRNTIERYKKACSDSSGS     79
Almond            FCKRRNGLLKKAYELSILCDAEVALIVFSSRGRLYEYSNN-NIRNTIERYKKACSDSSGS     79
S_Cherry          FCKRRNGLLKKAYELSILCDAEVALIVFSSRGRLYEYSNN-NIRNTIERYKKACSDSSGS     79
A_Pear            FCKRRNGLLKKAYELSILCDAEIALIVFSSRGRLYEYSNN-NIRNTIERYKKACSDSTGP     79
Apple             FCKRRNGLLKKAYELSILCDAEVALIVFSTRGRLYEYSNNNSIRNTIERYKKACSDSTGS     80
C_Pear            FCKRRNGLLKKAYELSILCDAEVALIVFSTRGRLYEYSNNNSIRNIIERYKKACSESTGS     80
Pomegranate       FCKRRNGLLKKAYELSVLCDAEVALIVFSSRGRLYEYSNN-NIKTTIERYKKASSDSANT     79
Pt_Poplar         FCKRRNGLLKKAYELSVLCDAEVSLIVFSSRGRLYEYANN-NIRSTIDRYKKVSSDSSNT     88
Pe_Poplar         FCKRRNGLLKKAYELSVLCDAEVALIVFSSRGRLYEYANN-NIRSTIDRYKKASSDSSNA     79
Pistachio         FCKRRNGLLKKAYELSILCEAEVALIVFSSRGRLYEYSNS-NIRSSIERYKKASSDNLNS     79
Cucumber          FCKRRNGLLKKAYELSVLCDAEVALIVFSSRGRLYEYSNN-SIKTTIERYKKACSDSSAT     79
Pumpkin           FCKRRNGLLKKAYELSVLCDAEVALIVFSSRGRLYEYSNN-SIKTTIDRYKKACSDSSAT     79
B_Melon           FCKRRNGLLKKAYELSVLCDAEVALIVFSSRGRLYEYSNN-SIKTTIERYKKACSDSSAT     79
MGrape_BB         FCKRRKGLLKKAYELSVLCDAEVALIVFSSRGRVYEYSNN-NIKSTLDRYKKASSDSTNA     79
BGrape_RL         FCKRRNGLLKKAYELSVLCDAQVALIVFSSRGRVYEYSNN-NIKSTIDRYKKASSDSTNG     79
MGrape_JB         FCKRRNGLLKKAYELSVLCDAEVALIVFSSRGRVYEYSNN-NIKSTIDRYKKASSDSTNA     79
MGrape_F          FCKRRNGLLKKAYELSVLCDAEVALIVFSSRGRVYEYSNN-NIKSTIDRYKKASSDSTNA     79
MGrape_FS         FCKRRNGLLKKAYELSVLCDAEVALIVFSSRGRVYEYSNN-NIKSTIDRYKKASSDSTNA     79
BGrape_RS         FCKRRNGLLKKAYELSVLCDAEVALIVFSSRGRVYEYSNN-NIKSTLDRYKKASSDSTNG     79
HGrape_BDB        FCKRRNGLLKKAYELSVLCDAEVALIVFSSRGRVYEYSNN-NIKSTIDRYKKASSDSTNG     79
BGrape_CH         FCKRRNGLLKKAYELSVLCDAEVALIVFSSRGRVYEYSNN-NIKSTIDRYKKASSDSTNG     79
BGrape_ST         FCKRRNGLLKKAYELSVLCDAEVALIVFSSRGRVYEYSNN-NIKSTIDRYKKASSDSTNG     79
Wild_grape        FCKRRNGLLKKAYELSVLCDAEVALIVFSSRGRVYEYSNN-NIKSTIDRYKKASSDSTNG     85
Eucalyptus        FCKRRNGLLKKAYELSVLCDAEVALIVFSSRGRLYEYSNN-SIRSTIERYKKANSDSSNT     79
W_Myrtle          FCKRRNGLLKKAYELSVLCDAEVALIVFSSRGRLYEYSNN-NIRSTIERYKKANSDSSNT     79
Gb_Cotton         FCKRRNGLLKKAYELSVLCDAEVALIVFSTRGRLYEYSNN-NIRSTIERYKKACSGTSNT     79
Clementine        FCKRRNGLLKKAYELSVLCDAEVALIVFSSRGRLYEYSNS-NIRSTIDRYKKACSDNSNS     79
Orange            FCKRRNGLLKKAYELSVLCDAEVALIVFSSRGRLYEYSNS-NIRSTIDRYKKACSDNSNS     86
CastorBean        FCKRRNGLLKKAYELSVLCDAEVALIVFSSRGRLYEYSNN-NIKSTIERYKKACSDSSNT     87
RubberTree        FCKRRNGLLKKAYELSVLCDAEVALIVFSSRGRLYEYCNNNSIKSTIERYKKACSDSSNT     94
Cassava           FCKRRNGLLKKAYELSVLCDAEVALIVFSSRGRLYEYSNN-NIKSTIERYKKACSDSSNT     79
Jatropha          FCKRRNGLLKKAYELSVLCDAEVALIVFSSRGRLYEYSNN-NIKSTIERYKKACSDSTNA     79
Gh_Cotton         FCKRRNGLLKKAYELSVLCDAEVALIVFSSRGRLYEYSNN-NIRSTIDRYKKACSDTSNT     79
Durian            FCKRRNGLLKKAYELSVLCDAEVALIVFSSRGRLYEYSNN-NIRSTIERYKKACSDSSNT     79
Cocoa             FCKRRNGLLKKAYELSVLCDAEVALIVFSSRGRLYEYSNN-NIRSTIERYKKACSDSSNT     79
Rye               FSKRRRGLFKKAEELAVLCDADVALVVFSSTGKLSQFASS-SMNEIIDKYSTHSKNLGKS     74
I_Rice            FSKRRRGLFKKAEELSVLCDADVALIVFSSTGKLSHFASS-SMNEIIDKYNTHSNNLGKA     79
J_Rice            FSKRRRGLFKKAEELSVLCDADVALIVFSSTGKLSHFASS-SMNEIIDKYNTHSNNLGKA     79
Rice              FSKRRRGLFKKAEELSVLCDADVALIVFSSTGKLSHFASS-SMNEIIDKYNTHSNNLGKA     79
Maize             FSKRRRGLFKKAQELSVLCDADVALIVFSSTGKLSQFASS-SMNEIIDKYNTHSKNLGKT     79
F_Millet          FSKRRRGLFKKAEELSVLCDADVALIVFSSTGKLSQFASS-SMNEIIDKYNTHSKNLGKA     79
Sorghum           FSKRRRGLFKKAEELSVLCDADVALIVFSSTGKLSQFASS-SMNEIIDKYNTHSKNLGKA     79
P_Ryegrass        FSKRRRGLFKKAEELSVLCDADVALIVFSSTGKLSQFASS-SMNEIIDKYSTHSKNLGKA     79
B_Wheat           FSKRRRGLFKKAEELSVLCDADVALIVFSSTGKLSQFASS-STNEIIDKYSTHSKNLGKT     79
D_Wheat           FSKRRRGLFKKAEELSVLCDADVALIVFSSTGKLSQFASS-SMNEIIDKYSTHSKNLGKT     79
Barley            FSKRRRGLFKKAEELSVLCDADVALIVFSSTGKLSQFASS-SMNEIIDKYSTHSKNLGKT     79
AO_palm           FSKRRRGLLKKAEELSILCDAEVALIVFSSTGKLYEYSSS-SMKEIIEKHSMHSKNLQKP     79
WM_Banana         FSKRRRGLFKKAAELSVLCDADVALIVFSSTGKLFEFCNS-SMKKIIDKHSTHSKNLEKH     79
W_Banana          FSKRRRGLFKKAAELSVLCDADVALIVFSSTGKLFEFCSS-SMKKILDKHSTHSKNLEKQ     79
                  *.***.**:*** **::**:*:::*:***: *:: .: .. . .  ::::    .

Radish            HSVQEINAAYYQQESAKLRQQIQTIQNSNRHLMGDSLSALSVKELKQVENRLEKAISRIR     138
Cabbage           HSVQEINAAYYQQESAKLRQQIQTIQNSNRNLMGDSLSALNVKELKQVENRLEKAISRIR     140
Rapeseed          HSVQEINAAYYQQESAKLRQQIQTIQNSNRNLMGDSLSALNVKELKQVENRLEKAISRIR     139
```

```
Turnip            HSVQEINAAYYQQESAKLRQQIQTIQNSNRNLMGDSLSALNVKELKQVENRLEKAISRIR    144
C_rubella         STVQEINAAYYQQESAKLRQQIQTIQNSNRNLMGDSLSALSVKELKQVENRLEKAISRIR    161
Camelina          STVQEINAAYYQQESAKLRQQIQTIQNSNRNLMGDSLSSLSVKELKQVENRLEKAISRIR    174
Arabidopsis       STVQEINAAYYQQESAKLRQQIQTIQNSNRNLMGDSLSSLSVKELKQVENRLEKAISRIR    165
T_Gourd           STVQEINAAYYQQESAKLRQQIQTIQNSNRNLMGDSLSSLSVKELKQVENRLEKAISRIR    139
W_Strawberry      TTITEINAQYYQQESTKLRHQIQMLQNSNRHLMGDSLSNLTVKELKQLENRLERGLTRIR    139
C_Rose            TTITEINAQYYQQESAKLRHQIQMLQNSNRHLMGDSLTNLTVKELKQLENRLERGLTRIR    139
W_Lupine          STATEINAQYYQQESAKLRQQIQMLQNSNRHLMGDALSTLTVKELKQLENRLERGITRIR    145
B_Lupine          STATEINAQYYQQESAKLRQQIQMLQNSNRHLMGDALSTLTVKELKQLENRLERGITRIR    161
WildPeanut        STATEINAQYYQQESAKLRQQIQMLQNSNRHLMGDALSTLTVKELKQLENRLERGITRIR    139
Peanut            STATEINAQYYQQESAKLRQQIQMLQNSNRHLMGDALSTLTVKELKQLENRLERGITRIR    139
Soybean           STTTEINAQYYQQESAKLRQQIQMLQNSNRHLMGDALSTLTVKELKQLENRLERGITRIR    139
PigeonPea         STTTEINAQYYQQESAKLRQQIQMLQNSNRHLMGDALSTLTVKELKQLENRLERGITRIR    139
CommonBean        STTTEINAQYYQQESAKLRQQIQMLQNSNRHLMGDALSTLTVKELKQLENRLERGITRIR    139
Chickpea          STTTEINAQYYQQESAKLRQQIQMLQNSNRHLMGDALSTLTVKELKQLENRLERGITRIR    139
Medicago          TTTTEINAQYYQQESAKLRQQIQMLQNSNRHLMGDALSTLTVKELKQLENRLERGITRIR    139
Peach             TSITEINAQYYQQESAKLRQQIQMLQNSNRHLMGDALSTLSVKELKQLENRLERGINRIR    140
J_Apricot         TSITEINAQYYQQESAKLRQQIQMLQNSNRHLMGDALSTLSVKELKQLENRLERGINRIR    139
Almond            TSITEINAQYYQQESAKLRQQIQMLQNSNRHLMGDALSTLSVKELKQLENRLERGINRIR    139
S_Cherry          TSITEINAQYYQQESAKLRQQIQMLQNSNRHLMGDALSTLSVKELKQLENRLERGINRIR    139
A_Pear            SSITEINAQYYQQESAKLRQQIQMLQNSNRHLMGDALSNLTVKELKQLENKLERGMTRIR    139
Apple             SSVTEINAQYYQQESAKLRQQIQMLQNSNRHLMGDALSTLTVKELKQVENRLERGITRIR    140
C_Pear            TSVTEINAQYYQQESAKLRQQIQMLQNSNRHLMGDALSTLSVKELKQVENRLERGITRIR    140
Pomegranate       TSVIEINAQYYQQESAKLRQQIQML-----HLMGDSLSALSVKELKQLENRLERGITRIR    134
Pt_Poplar         ASITEINAQYYQQESAKMRQQIQLLQNSNRHLMGEAVSNLSVKELKQLENRLERGMTRIR    148
Pe_Poplar         SSITEINAQYYQQESAKLRQQIQMLQNSNRHLMGDAVSNLSVKELKQLENRLERGITRIR    139
Pistachio         GSVIEINAQYYQQESAKLRQQIQMIQNSNRHLMGESLNTLTVKELKQLENRLERGLTRIR    139
Cucumber          SSVTELNTQYYQQESAKLRQQIQMLQNSNRHLMGDSLSALTVKELKQLENRLERGITRIR    139
Pumpkin           SSVTELNTQYYQQESAKLRQQIQMLQNSNRHLMGDSLSALTVKELKQLENRLERGITRIR    139
B_Melon           SSVTELNTQYYQQESAKLRQQIQMLQNSNRHLMGDSLSALTVKELKQLENRLERGITRIR    139
MGrape_BB         GSTLEINAQYYQQESAKXRQQIQMLQNXNRHLMGDSLASLTVKELKQLENRLERGITRIR    139
BGrape_RL         GSTMEIHAQYYQQESAKLRQQIQMLQNSNRHLMGDSLASLTVKELKQLENRLERGITRIR    139
MGrape_JB         GSTMEINAQYYQQESAKLRQQIQMLQNSNRHLMGDSLASLTVKELKQLENRLERGITRIR    139
MGrape_F          GSTMEINAQYYQQESAKLRQQIQMLQNSNRHLMGDSLASLTVKELKQLENRLERGITRIR    139
MGrape_FS         GSTMEINAQYYQQESAKLRQQIQMLQNSNRHLMGDSLASLTVKELKQLENRLERGITRIR    139
BGrape_RS         GSTMEINAQYYQQESAKLRQQIQMLQNSNRHLMGDSLASLTVKELKQLENRLERGITRIR    139
HGrape_BDB        GSTMEINAQYYQQESAKLRQQIQMLQNSNRHLMGDSLASLTVKELKQLENRLERGITRIR    139
BGrape_CH         GSTMEINAQYYQQESAKLRQQIQMLQNSNRHLMGDSLASLTVKELKQLENRLERGITRIR    139
BGrape_ST         GSTMEINAQYYQQESAKLRQQIQMLQNSNRHLMGDSLASLTVKELKQLENRLERGITRIR    139
Wild_grape        GSTMEINAQYYQQESAKLRQQIQMLQNSNRHLMGDSLASLTVKELKQLENRLERGITRIR    145
Eucalyptus        STVTEINAQYYQQESAKLRQQIQMLQNSNRHLMGDSLSSLSVKELKQLENRLERGITRIR    139
W_Myrtle          STVTEINAQYYQQESAKLRQQIQMLQNSNRHLMGDSLSSLSVKELKQLENRLERGITRIR    139
Gb_Cotton         NTVTEINAQYYQQESAKLRQQIQMLQNSSRHLMGDSLSSLTVKELKQLENRLERGITRIR    139
Clementine        GTVTEINAQYYQQESAKLRQQIQMLQNSNRHLMGDSLSSLTVKELKQLENRLERGITRIR    139
Orange            GTVTEINAQYYQQESAKLRQQIQMLQNSNRHLMGDSLSSLTVKELKQLENRLERGITRIR    146
CastorBean        SSITEINAQYYQQESAKLRQQIQMLQNSNRHLMGDSLSSLTVKELKQLENRLERGITRIR    147
RubberTree        SSITEINAQYYQQESAKLRQQIQMLQNSNRHLMGDSLSSLTVKELKQLENRLERGITRIR    154
Cassava           SSITEINAQYYQQESAKLRQQIQMLQNSNRHLMGDSLSSLTVKELKQLENRLERGITRIR    139
Jatropha          SSITEINAQYYQQESAKLRQQIQMLQNSNRHLMGDSLSSLTVKELKQLENRLERGITRIR    139
Gh_Cotton         NTVTEINAQYYQQESAKLRQQIQMLQNSNRHLMGDSLSSLTVKELKQVENRLERGITRIR    139
Durian            NTVTEINAQYYQQESAKLRQQIQMLQNSNRHLMGDSLSSLTVKELKQLENRLERGITRIR    139
Cocoa             NSVIEINAQYYQQESAKLRQQIQMLQNSNRHLMGDSLSSLTVKELKQLENRLERGITRIR    139
Rye               DQQPAIDLNLEHCKYDSLNEQLAEASLRLRHMRGEELDGLSVGELQQMEKNLETGLQRVL    134
I_Rice            E-QPSLDLNLEHSKYAHLNEQLAEASLRLRQMRGEELEGLSIDELQQLEKNLEAGLHRVM    138
J_Rice            E-QPSLDLNLEHSKYAHLNEQLAEASLRLRQMRGEELEGLSIDELQQLEKNLEAGLHRVM    138
Rice              E-QPSLDLNLEHSKYAHLNEQLAEASLRLRQMRGEELEGLSIDELQQLEKNLEAGLHRVM    138
Maize             E-QPSLDLNLEHSKYANLNEQLAEASLRLRQMRGEELEGLNVEELQQLEKNLESGLHRVL    138
F_Millet          E-QPSLDLNLEHSKYANLNEQLTEASLRLRQMRGEELEGLSVDELQQLEKNLEAGLHRVL    138
Sorghum           E-EPSLDLNLEHSKYANLNEQLVEASLRLRQMRGEELEGLSVEELQQLEKNLETGLHRVL    138
P_Ryegrass        D-QPSLDLNLEHSKYANLNDQLAEASLRLRQMRGEGLEGLTVDELQQLEKNLETGLHRVL    138
B_Wheat           D-QPALDLNLEHSKYANLNDQLAEASLRLRQMRGEELEGLSVDELQQLEKNLETGLHRVL    138
D_Wheat           D-QPALDLNLEHSKYANLNDQLAEASLRLRQMRGEELEGLSVDELQQLEKNLETGLHRVL    138
Barley            D-QPTLDLNLEHSKYANLNDQLAEASLRLRQMRGEELEGLSVDELQQLEKNLETGLHKVL    138
AO_palm           D-QPPLDLNLENSKHASLSKQVADMSLQLRQMRGEELEGLSIEELQQLEKALEAGLSRVI    138
WM_Banana         D-QPFLDLNLDNNNYASLKKQVAEASLQLRQMRGEALEKLTVEELQQLEKTLEAGLGRVM    138
W_Banana          D-QLSLDLNLDSKH-VSLKKQVAEASLQLRQMRGEALESLTLEELQQLEKTLETGLDRVV    137
                       :.      .     .*:        :: *: :  *.: **:*:*: ** .: ::

Radish            SKKHELLLAEIENLQKREIELDNESIYLRTKIAEVERFQQHHHQMVSGTEMAAIEVLA-S    197
Cabbage           SKKHELLLAEIENLHKREIKLDNESIYLRTKIAEVERFQQHHHQMVSGTEMTAIEALA-S    199
Rapeseed          SKKHELLLAEIENLHKREIKLDNENIYLRTKIAEVERFQQHHHQMVSGTEMTAMEALA-S    198
Turnip            SKKHELLLAEIENLHKREIKLDNESIYLRTKIAEVERFQQHHHQMVSGTEMTAIEALA-S    203
C_rubella         SKKHELLLAEIENMQKREIELDNENIYLRTKVAEVERFQQQHHQMVSGSEINAMEALA-A    220
Camelina          SKKHELLLAEIENMQKREIELDNENIYLRTKVAEVERFQQHHHQMVSGSEINAIEALAAS    234
```

```
Arabidopsis       SKKHELLLVEIENAQKREIELDNENIYLRTKVAEVERYQQHHHQMVSGSEINAIEALA-S    224
T_Gourd           SKKHEMLLVEIENAQKREIELENENLYLRTKVAEVERIQ-HHHQMVSGPELNAIEALA-S    197
W_Strawberry      SKKHEMLLAEIEYLQKREIELENENVLIRAKIAEVERLQQA--DLVSGAELNAIQALA-S    196
C_Rose            SKKHEMLLAEIEYLQKREVELENENVLIRAKIAEVERLQQA--DLVSGAEFNAIQALA-S    196
W_Lupine          SKKHEMLLAEIEYLQKREIELENENLCIRTKIAEGERLQQA--NMVSGQELNAIQALA-S    202
B_Lupine          SKKHEMLLAEIEYLQKREIELENENLCIRTKIGEVERVQQA--NMVSGQELNAIQALA-S    218
WildPeanut        SKKHEMLLAEIEYFQKREIELENENLCLRTKITDVERIQQA--NMVSGPELNAIQALA-S    196
Peanut            SKKHEMLLAEIEYFQKREIELENENLCLRTKITDVERIQQA--NMVSGPELNAIQALA-S    196
Soybean           SKKHEMLLAEIEYFQKREIELENENLCLRTKITDVERIQQV--NMVSGPELNAIQALA-S    196
PigeonPea         SKKHEMLLAEIEYFQKREIELENENLCLRTKITDVERLQQV--NMVSGPELNAIQALA-S    196
CommonBean        SKKHEMLLAEIEYFQKREIELENENLCLRTKITDVERIQQV--NMVSGPELNAIQALA-S    196
Chickpea          SKKHEMLLAEIEYFQKREIELENENLCLRSKINDVERLPQV--NMVSGQELNAIQALA-S    196
Medicago          SKKHEMLLAEIEYFQKREIELENENLCLRTKINDVERLPQV--NMVSGQELNAIQALA-S    196
Peach             SKKHEMLLAEIEYLQKKEIELENENVCLRTKISEVERLQQA--NMV-GPELNAIQALA-S    196
J_Apricot         SKKHEMLLAEIEYLQKKEIELENENVCLRTKISEVERLQQA--NMV-GPELNAIQALA-S    195
Almond            SKKHEMLLAEIEYLQKKEIELENENVCLRTKISEVERLQQA--NMV-GPELNAIQALA-S    195
S_Cherry          SKKHEMLLAEIEYLQKKEIELENENVCLRTKISEVERLQQA--NMV-GPELNAIQALA-S    195
A_Pear            SKKDEMLIAEIEYLQKKEIELENENVYLRTKISEVERLHQA--NMVSVPEMNAIQALA-S    196
Apple             SKKHELLLAEIEYFQKKEIELENENVYLRTKVSEVERLQQA--NMVSGSEMNAIQALA-S    197
C_Pear            SKKHELLLAEIEYFQKKEIELENENVYLRTKISEVERLQQA--NMVPGSEMNAIQALA-S    197
Pomegranate       SKKHEMLLCEIEYLQKKEIEMENESVYLRTKIAEIERMEQA--NMVPGQEMNAIQVLA-S    191
Pt_Poplar         SKKHELLLAEIEYMQKREIELENESACLRTKIAEVERLQQA--NMVTGEELNAIQALAAS    206
Pe_Poplar         SKKHELLLAEIEYLQKREIELENESVCLRTKIAEVERLQQA--NMVTGAELNAIQALAAS    197
Pistachio         SKKHEMLLAEIEYFQKREIELENESVFLRSKIAEVERFQKA-NNIVSGPELNAIHALA-S    197
Cucumber          SKKHEMLLAEIEYLQKREIELENENVCIRTKIAEVERVQQA--NMVSGQELNAIQALANS    197
Pumpkin           SKKHEMLLAEIEYLQKREIELENENVCIRTKIAEVERLHQA--NMESGQELNAIQALA-S    196
B_Melon           SKKHEMLLAEIEYLQKREIELENENVCIRTKIAEVERLQQA--NMVSGQELNAIQALA-S    196
MGrape_BB         SKKHELLLAEIEYLQKREIELENESVYLRTKIAEVERLQQA--NMVSTHEFNAIQALV-S    196
BGrape_RL         SKKHELLLAEIEYLQKREIELENESVYLRTKIAEVERLQQA--NMVSTHEFNAIQALV-S    196
MGrape_JB         SKKHELLLAEIEYLQKREIELENESVYLRTKIAEVERLQQA--NMVSTHEFNAIQALV-S    196
MGrape_F          SKKHELLLAEIEYLQKREIELENESVYLRTKIAEVERLQQA--NMVSTHEFNAIQALV-S    196
MGrape_FS         SKKHELLLAEIEYLQKREIELENESVYLRTKIAEVERLQQA--NMVSTHEFNAIQALV-S    196
BGrape_RS         SKKHELLLAEIEYLQKREIELENESVYLRTKIAEVERLQQA--NMVSTHEFNAIQALV-S    196
HGrape_BDB        SKKHELLLAEIEYLQKREIELENESVYLRTKIAEVERLQQA--NMVSTHEFNAIQALV-S    196
BGrape_CH         SKKHELLLAEIEYLQKREIELENESVYLRTKIAEVERLQQA--NMVSTHEFNAIQALV-S    196
BGrape_ST         SKKHELLLAEIEYLQKREIELENESVYLRTKIAEVERLQQA--NMVSTHEFNAIQALV-S    196
Wild_grape        SKKHELLLAEIEYLQKREIELENESVYLRTKIAEVERLQQA--NMVSTHEFNAIQALV-S    202
Eucalyptus        SKKHEMLLTEIEYLQKKEIELENESVFLRTKIAEVDRIQQG--NMVAGPQVNVMEALA-S    196
W_Myrtle          SKKHEMLLTEIEYLQKREIELENESVFLRTKIAEVERIQQG--NMVAGPQLNAMEALA-S    196
Gb_Cotton         SKKHEMLLAEIEYFQKREVGLENESVCLRAKIAEIERVEEA--NMVTGAELNAIQALA-S    196
Clementine        SKKHEMLLAEIEFLQKREIELENESVCLRSKIAEMERFQQA--NMVTGQELNAIHALA-S    196
Orange            SKKHEMLLAEIEFLQKREIELENESVCLRSKIAEMERFQQA--NMVTGQELNAIHALA-S    203
CastorBean        SKKH-LLLAEIEYLQKREIELENESVCLRTKIAEIERLQQA--NMVTGAELNAIQALT-S    203
RubberTree        SKKHELLLAEIEYLQKREIELENESVCLRTKIAEIERLQQA--NMVTGAELNAIQALT-S    211
Cassava           SKKHELLLAEIEYLQKREIELENESVCLRTKIAEIERLQQA--NMVTGAELNAIQALT-S    196
Jatropha          SKKHELLLAEIEYLQKREIELENESVCLRTKIAEIERLQQA--NMVTGAELNAIQALT-S    196
Gh_Cotton         SKKHEMLLAEIEFLQKREIELENESVCLRTKIAEIERLQQA--NMVTGPELNAIQALA-S    196
Durian            SKKHEMLLAEIEYMQKRETELENESVCLRTKIAEIERLQQA--NMVSGPELNAIQALT-S    196
Cocoa             SKKHEMLLAEIEYLQKREIELENESVCLRTKIAEIERLQQA--NMVTGPELNAIQALA-S    196
Rye               CTKDRQFMQQISDLQQKGTQLAEENMRLKNQMHEVPTASMV---AVADAE-NVVPDDVHS    190
I_Rice            LTKDQQFMEQISELQRKSSQLAEENMQLRNQVSQISPAEKQ----VVDTE-NFVTEEGQS    193
J_Rice            LTKDQQFMEQISELQRKSSQLAEENMQLRNQVSQISPAEKQ----VVDTE-NFVT-EGQS    192
Rice              LTKDQQFMEQISELQRKSSQLAEENMQLRNQVSQISPAEKQ----VVDTE-NFVT-EGQS    192
Maize             QTKDQQFLEQINDLERKSTQLAEENMQLRNQVSQIPPAGKQ---AVADTE-NVIAEEGQS    194
F_Millet          QTKDQQFLEQISELQRKSSQLAEENMQLRNQVSQIPPAGKQ---AVAETE-NVIAEDGQS    194
Sorghum           QTKDQQFLEQISDLERKSTQLAEENMQLRNQVSQIPPAGKQ---AVADTE-NVIAEDGQS    194
P_Ryegrass        QTKDQQFLEQINELQRKSSQLAEENMQLRNQVSQIPIAGKP---VVADTE-NVIAEDGQS    194
B_Wheat           QTKDQQFLEQINELHRKSSQLAEENMKLRNQVGQIPTAGKL---VVADTE-NVVAEDGQS    194
D_Wheat           QTKDQQFLEQINELHRKSSQLAEENMKPRNQVGQIPTAGKL---VVADTE-NVVAEDGQS    194
Barley            QTKDQQFLEQINELHRKSSQLAEENKKLRNQVAQVPTAGKL---VVVDTE-NVIAEDGQS    194
AO_palm           DRKGEQIMEQINGLQQKGLKLMEENTRLREQVLKLSRVGKQ---MMNDKE-NALNEDGQS    194
WM_Banana         DRKGAQFTQQINSLQQKAAKLAEENVRLRRRVLEMPNMGKQ---VMADKD-NVVNEDGQS    194
W_Banana          DRKGTQIMQQISTLQQKALQLAEENVRLRQRAMEMPNLGKH---IMAEKE-NVVNEDGQS    193
                    *   :  :*.  .::   : :*.   : :  .               :   :     :

Radish            RNYFAHGIMATGSGSGAGHGCSYSDP-DKKI-HLG----------    230
Cabbage           RNYFAHNIMTIGSGSGAGHGCSYSDP-DKKT-HLG----------    232
Rapeseed          RNYFAHNIMTIGSGSGAGHGCSYFDP-DKKT-HLG----------    231
Turnip            RNYFAHNIMTIGSGSGAGHGCSYFDP-DKKT-HLG----------    236
C_rubella         RNYFGHSIMTAGSGSG--NGGSYSDP-DKKILHLG----------    252
Camelina          RNYFAHSIMTAGSGSG--NGGSYSDP-DKKILHLG----------    266
Arabidopsis       RNYFAHSIMTAGSGSG--NGGSYSDP-DKKILHLG----------    256
T_Gourd           RNYFNPSIMTCS-GS---GNGSYSDS-DKKILHLG----------    227
W_Strawberry      RNFFESTMMEGE--------TSYSQP-EKKLLHLG----------    222
```

```
C_Rose           RNFFGSTMVEGE--------ASYSQP-EKKLLHLG----------    222
W_Lupine         RNFFNPSMMET--------GAAYPQP-DKKILHIGYL--------    230
B_Lupine         RNFFNPSIMET--------GAAYPQP-DKKILHIG----------    244
WildPeanut       RNFFNQSMIDQTA------GTAYPQSSDKKILHLG----------    225
Peanut           RNFFNQSMIDQTA------GTAYPQSSDKKILHLGAGESCSFEE-    234
Soybean          RNFFNPNMLEGG--------TVYP-HSDKKILHLG----------    222
PigeonPea        RNFFNPNMMEGG--------SVYP-QSDKKILHLG----------    222
CommonBean       RNFFNPNMMEGG--------SVYP-QSDKKILHLG----------    222
Chickpea         RNFFNPNMMEDG--------TSYP-QSDKKILHLG----------    222
Medicago         RNFFNPNMMEDG-------ETSYH-QSDKKILHLG----------    223
Peach            RNFFSQNMMEG--------GATYPQQ-DKKILHLG----------    222
J_Apricot        RNFFSQTMMEG--------GATYPQQ-DKKILHLG----------    221
Almond           RNFFSQNMMEG--------GATYTQQ-DKKILHLG----------    221
S_Cherry         RNFFSQNMMEG--------GATYPQQ-DKKILHLG----------    221
A_Pear           RNFFSQNIIEG-G------GATFPQQ-NKKILHLG----------    223
Apple            RHFFSQNMIEG-G------GATFPQQ-DKKNLHLG----------    224
C_Pear           RNFFSQNMIEG-G------GATFPQQ-DKKNLHLG----------    224
Pomegranate      RNFFPPNMLEGGN--------SYSHP-DKK-LHLGYESIILCILV    226
Pt_Poplar        RNFFAPHFLEGGT------A-YPHTY-NKKILHLG----------    233
Pe_Poplar        RNFFAPHLLEGGT------A-YPHN--DKKILHLG----------    223
Pistachio        RNFFNPTMIENTE-------TAYSHP-DKKMLHLG----------    224
Cucumber         RNFFSPNIMEPAG------PVSYSHQ-DKKMLHLG----------    225
Pumpkin          RNFFSPNIME-GG------AVTFSHQ-D-----------------    216
B_Melon          RNFFTPNMME-GG------AVTFSHQ-DKKMLHLG----------    223
MGrape_BB        RNFFQPNMIEGGS-------TGYPLP-DKKVLHLG----------    223
BGrape_RL        RNFFQPNMIEGGS-------TGYPLP-DKKVLHLG----------    223
MGrape_JB        RNFFQPNMIEGGS-------TGYPLP-DKKVLHLG----------    223
MGrape_F         RNFFQPNMIEGGS-------TGYPLP-DKKVLHLG----------    223
MGrape_FS        RNFFQPNMIEGGS-------TGYPLP-DKKVLHLG----------    223
BGrape_RS        RNFFQPNMIEGGS-------TGYPLP-DKKVLHLG----------    223
HGrape_BDB       RNFFQPNMIEGGS-------TGYPLP-DKKVLHLG----------    223
BGrape_CH        RNFFQPNMIEGGS-------TGYPLP-DKKVLHLG----------    223
BGrape_ST        RNFFQPNMIEGGS-------TGYPLP-DKKVLHLG----------    223
Wild_grape       RNFFQPNMIEGGS-------TGYPLP-DKKVLHLG----------    229
Eucalyptus       RNFFPSNMVEGGT--------AYSHS-DKKVLHLG----------    222
W_Myrtle         RNFFPANMVEGGA--------AYSHS-DKKVLHLG----------    222
Gb_Cotton        RNFFTPNVIERGT------PTPYSHH-DKKILHLG----------    224
Clementine       RNFFSPAIIEGGV-------TAYSHP-DKKILHLG----------    223
Orange           RNFFSPAIIEGGG-------TAYSHP-DKKILHLG----------    230
CastorBean       RNFFGSHMIEGGA-------AYSHPS-DKKILHLG----------    230
RubberTree       RNFFGPHMIEGGT-------AYSHPD--KKILHLG----------    237
Cassava          RNFFGPHMIEGGT-------AYSHPD--KKILHLG----------    222
Jatropha         RNFFGPHMIEDGT-------AYSHPD--KKILHLG----------    222
Gh_Cotton        RNFFSPNVIEHPS-------AYSHPS-DKKILHLG----------    223
Durian           RNFFSPNVIEGGI-------AYSHPD--KKILHLG----------    222
Cocoa            RNFFSPNVIEGGT-------AYSHPD--KKILHLG----------    222
Rye              SDSVMTAVHSASS-------QDNDDG-SDIS--------------    213
I_Rice           SESVMTALHSGSSQ-----SQDNDDG-SDVSLKLGLPCGAWK---    229
J_Rice           SESVMTALHSGSSQ-----SQDNDDG-SDVSLKLGLPCGAWK---    228
Rice             SESVMTALHSGSSQ-----SQDNDDG-SDVSLKLGLPCGAWK---    228
Maize            SESVMTALHSGSS-------QDNDDG-SDVSLKLGLPCVAWK---    228
F_Millet         SESVMTALHSGSS-------QDNDDG-SDVSLKLGLPCVAWK---    228
Sorghum          SESVMTALHSGSS-------QDNDDG-SDVSLKLGLPCVAWK---    228
P_Ryegrass       SESVMTALHSGSS-------QDNDDG-SDVSLKLGLPCSAWK---    228
B_Wheat          SESVMTALHSGSS-------QDNDDG-SDVSLKLGLPCLPWK---    228
D_Wheat          SESVMTALHSGSS-------QDNDDG-SDVSLKLGLPCLPWK---    228
Barley           SESVMTALHSGSS-------QDNDDG-SDVSLKLAL---PWK---    225
AO_palm          SESVTNVLNPGGS-------QDYDDS-SDTSLKLGLPGSNWK---    228
WM_Banana        SESVTNASHSGGP-------QECDDS-SVTSLKLGLPYC------    225
W_Banana         SESVTNALHPGGP-------QECDDS-SVTSLKLGQN--------    222
                   . .
```

Figure S3: Multiple alignment of the amino acid sequences of all AGL11 or AGL11-like proteins of monocot and Eudicot plants. The deduced amino acid sequences of AGL11 protein of all muscadine grape (cvs. Black Beauty, BB; Jane Bell, JB; Fry, F; and Fry Seedless, FS), hybrid Bunch grape (cv. Blanc du Bois, BDB) and Bunch grape (Riesling, RL and Reliance Seedless, RS) sequenced and reported in this study were included along with that of published Bunch grape cultivars Chardonay (CH) and Sultanina (ST). The accession numbers of the amino acid sequences of AGL11 or AGL11-like protein of monocots and eudicots are provided in Table S3.

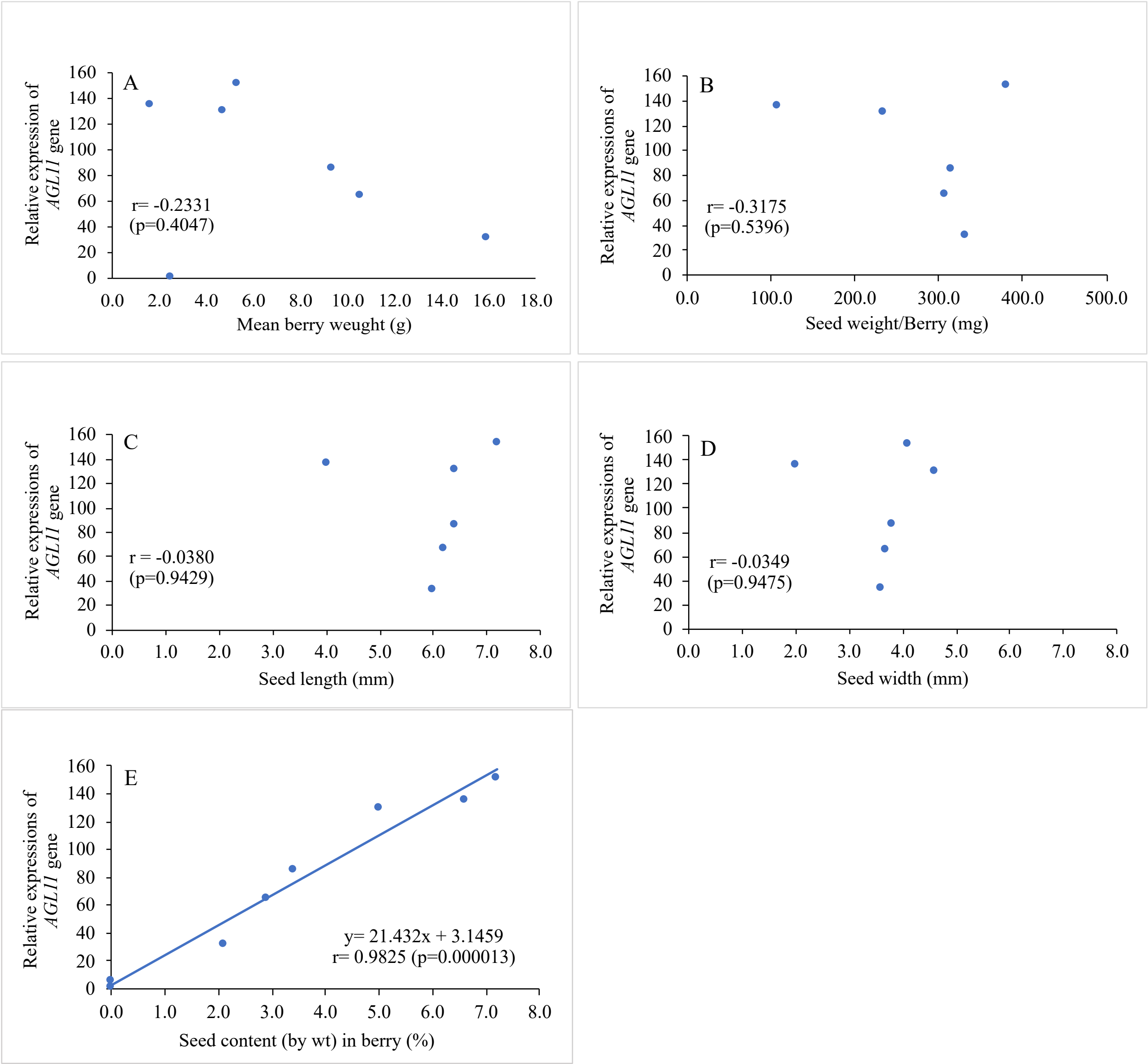

Figure S4. Scatter plots showing relationship between relative expression of *AGL11* gene in pea-size berries of selected muscadine and Bunch grape cultivars and A) mean berry weight; B) seed weight/Berry; C) seed length; D) seed width; and E) seed content (by weight) in berry (%). The seeded and seedless muscadine grape cultivars, Jane Bell, Noble, Dixie Red, Black Fry, Black Beauty and Fry Seedless; and Bunch grape cultivars, Riesling and Reliance seedless were included in this study. r= coefficient of correlation and p= significance level.
